# Atropisomerism in Diarylamines: Structural Requirements and Mechanisms of Conformational Interconversion

**DOI:** 10.1002/anie.202007595

**Published:** 2020-08-20

**Authors:** Romain Costil, Alistair J. Sterling, Fernanda Duarte, Jonathan Clayden

**Affiliations:** ^1^ School of Chemistry University of Bristol Cantock's Close BS8 1TS Bristol UK; ^2^ Chemistry Research Laboratory Oxford University Mansfield Road OX1 3TA Oxford UK

**Keywords:** atropisomers, diarylamines, dynamic stereochemistry, free energy surface, molecular dynamics

## Abstract

In common with other hindered structures containing two aromatic rings linked by a short tether, diarylamines may exhibit atropisomerism (chirality due to restricted rotation). Previous examples have principally been tertiary amines, especially those with cyclic scaffolds. Little is known of the structural requirement for atropisomerism in structurally simpler secondary and acyclic diarylamines. In this paper we describe a systematic study of a series of acyclic secondary diarylamines, and we quantify the degree of steric hindrance in the *ortho* positions that is required for atropisomerism to result. Through a detailed experimental and computational analysis, the role of each *ortho*‐substituent on the mechanism and rate of conformational interconversion is rationalised. We also present a simple predictive model for the design of configurationally stable secondary diarylamines.

## Graphical abstract

Hindered secondary diarylamines Ar^1^NHAr^2^ can show chirality due to restricted rotation about their C−N bonds. Using a combination of dynamic analytical methods and computational models, we describe the requirements for atropisomerism in these diarylamines and provide a model for estimating their barrier to interconversion.

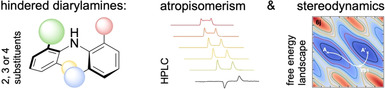

## Introduction

The most established classes of atropisomers, biphenyls and binaphthyls, have found application as chiral ligands,^1^ chiral auxiliaries,^2^ and sensors.^3^ Yet beyond these archetypical structures, atropisomers whose chirality does not arise from a C_aryl_−C_aryl_ bond also play an increasing role in chemistry.^4^, ^5^, ^6^ This is especially the case in the field of medicinal chemistry, where molecules frequently contain multiply functionalised aromatic rings and hindered amide groups, the very features that also give rise to atropisomerism.^7^, ^8^

**Figure 1:**
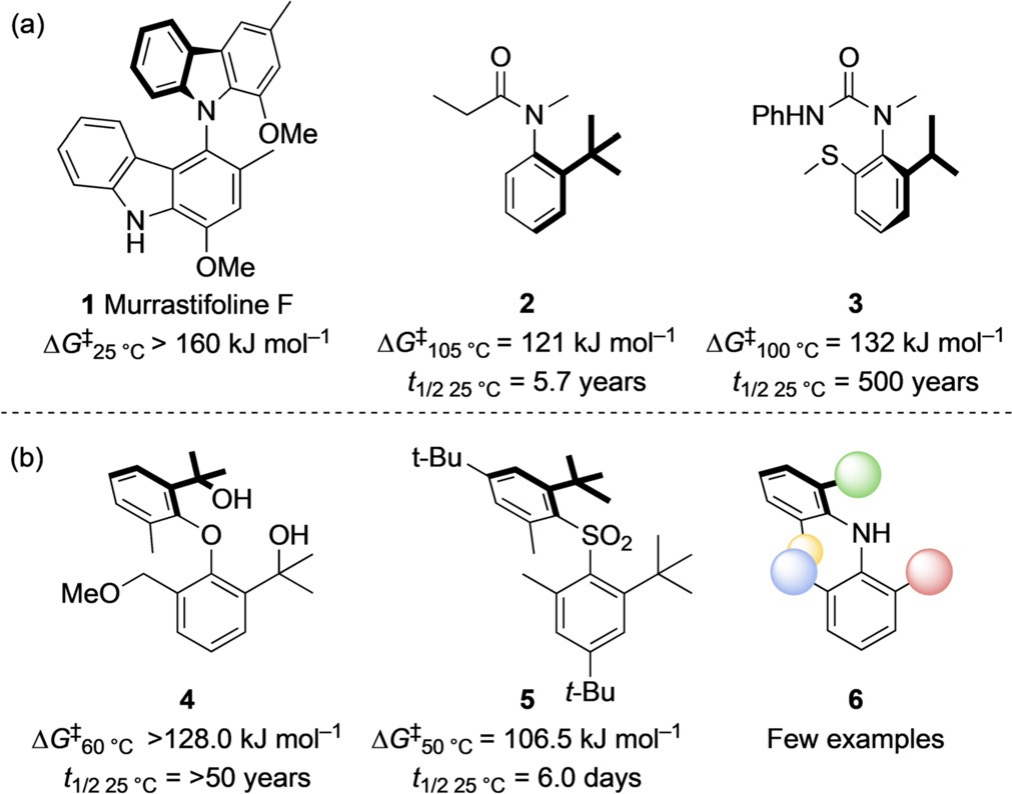
a) Natural and synthetic C−N atropisomers. b) Diaryl ether and diaryl sulfone atropisomers. Barriers to interconversion of enantiomers are shown, along with estimated half‐lives for racemisation at ambient temperature.

The range of methods for the construction of C−N bonds, coupled with the ability of the nitrogen atom to adopt planar geometry by delocalization of its lone pair, has made atropisomerism about a C−N bond a fruitful area of investigation. C−N atropisomerism has also been noted in natural products such as the biscarbazole murrastifoline F,^9^ the naphthylisoquinoline alkaloid ancisheynine,^10^ and marinopyrrole A^11^ (Figure 1).

C−N atropisomers in which the nitrogen atom is not part of a ring include the important families of anilides **2** 12 and ureas **3**.^13^ However, examples of atropisomeric, acyclic *N*‐aryl anilines are scarce. Recently, we found that a remarkably versatile conformationally‐accelerated variant of the Smiles rearrangement allows the synthesis of tertiary diarylamines with sufficient steric hindrance around the two C−N bonds to permit resolution by HPLC on a chiral stationary phase.^14^ Kitagawa and co‐workers studied the influence of electronic factors on the kinetic of racemisation around one^15^ or two^16^ C−N bonds in tertiary diarylamines.

Secondary diarylamines, in which steric hindrance is less prominent than in their tertiary analogues, may also exhibit atropisomerism. The Kawabata group reported a series of sterically hindered diarylamines with a single, slowly rotating C−N bond,^17^ and demonstrated the importance of an internal hydrogen bond on its barrier to rotation.^18^, ^19^ This strategy of stabilizing configuration through intramolecular hydrogen bonding was elegantly used by Gustafson et al. for the enantioselective synthesis of *N*‐aryl quinoid derivatives.^20^ However, no secondary diarylamine in which atropisomerism arises solely from steric hindrance has so far been reported.

Diarylamines are closely related in architecture to the conformationally stable diaryl ethers such as **4** 21, 22 and sulfones such as **5**,^23^ whose configurational stability was found to derive rather subtly from both the steric hindrance and symmetry of the substituted aryl rings. Given the potential utility of atropisomeric amines as chiral ligands,^24^, ^25^ and the importance of exerting fine control over the rate of conformer interconversion in potentially atropisomeric drug targets,^7^, ^8^ we have investigated in detail the factors that determine the rates of C−N bond rotation, and hence the structural requirements for atropisomerism, in this family of hindered *N*‐aryl anilines.

Computational protocols, including molecular mechanics (MM)^21^, ^26^ and quantum mechanics (QM)^15^, ^16^, ^26^, ^27^, ^28^ approaches, have been employed to quantify the stability of various atropisomers, providing insights into the electronic and steric factors determining their barriers to conformational interconversion. For example, in the case of aromatic amides whose conformational interconversion involves a single rotating bond, an inexpensive QM‐based scan protocol provided a good correlation (*r* ^2^=0.8) between experimental and calculated energy barriers.^27^, ^29^ For systems involving two rotatable bonds, as is the case for diarylamines,^15^, ^16^ diaryl ethers **4** 21 and sulfones **5**,^23^ transition state structures have also been postulated. However, mechanistic analysis of these systems has been scarce, perhaps due to the more complex free energy surface. Indeed, employing MM approaches, we have previously shown that the inclusion of even small substituents in diaryl ethers can substantially alter the mechanism, and consequently the rates of racemisation.^21^

Here we explore how different *ortho*‐substituents affect the conformational flexibility of secondary diarylamines and the rate and mechanism by which their conformers interconvert. To do so, we employ classical molecular dynamics (MD) in explicit solvent to allow the efficient exploration of free energy surfaces (FESs), and QM techniques to characterise the transition states (TS) and calculate the barriers to interconversion of the conformers or atropisomers.

## Results and Discussion

**Scheme 1:**
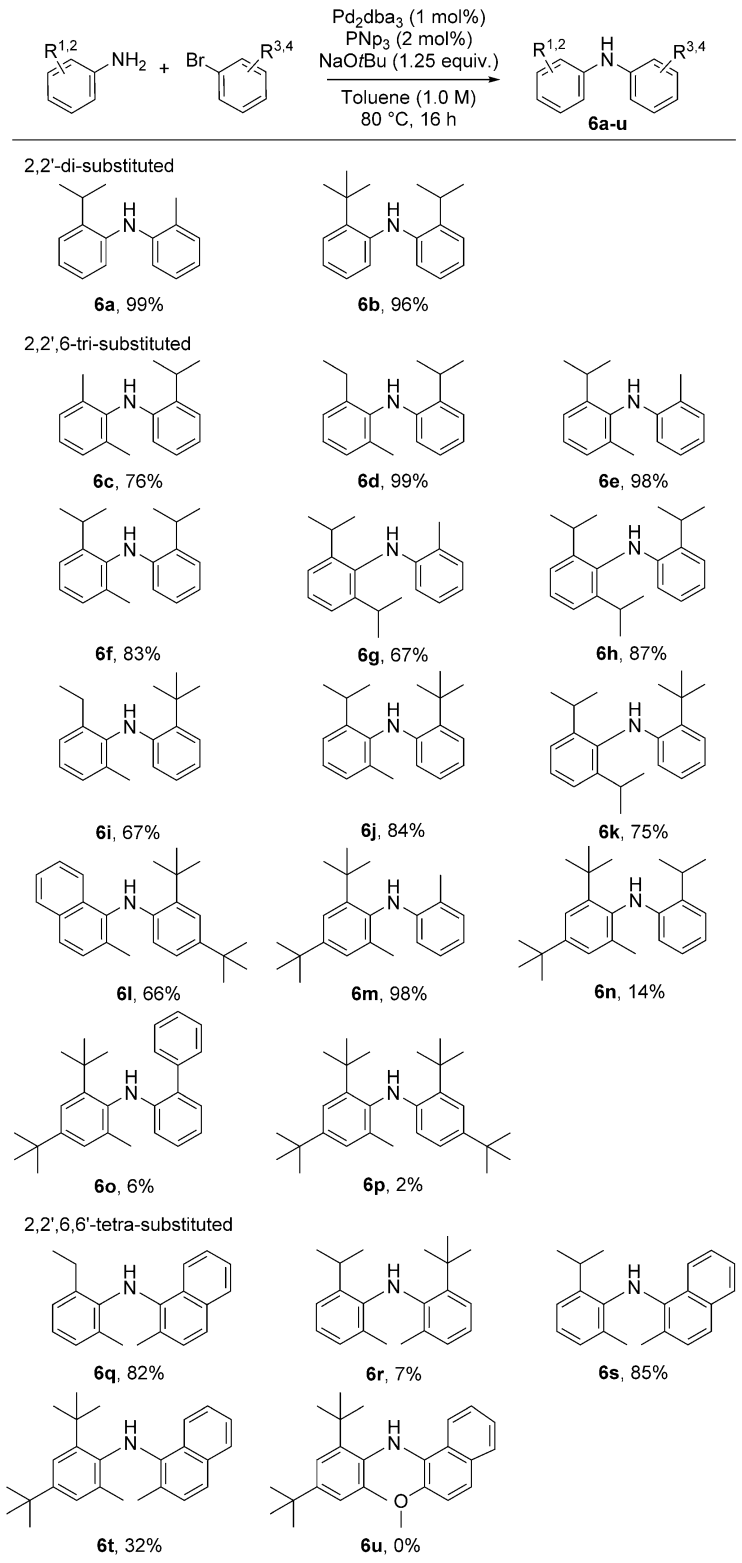
Synthesis of sterically hindered secondary diarylamines.

An initial series of diarylamines carrying two or more *ortho*‐alkyl groups was synthesized by Buchwald–Hartwig coupling of corresponding anilines and aryl bromides (Scheme 1). These particularly hindered products could be made using Shaughnessy's trineopentylphosphine (PNp_3_) ligand.^30^ While di‐ and tri‐*ortho‐*substituted compounds were formed in good to excellent yields, the synthesis of tetra‐*ortho* substituted diarylamines, even with PNp_3_, was sluggish and low‐yielding.

Evidence that the diarylamines adopt a non‐planar ground state was provided by the appearance of signals of inequivalent chemical shifts in the ^1^H NMR spectra at room temperature for **6 d**, **6 f**, **6 g** and at 10 °C for **6 e** (see Supporting Information, Figures S1–6) due to the presence of diastereotopic groups in their ethyl or isopropyl groups. 2,2′‐Di‐*ortho*‐substituted diarylamines **6 a**–**c** were insufficiently hindered for slow rotation of the C−N bond to be detectable by ^1^H NMR. Fast exchange was evident even at −40 °C, suggesting that if the ground state is chiral, the enantiomeric conformers interconvert rapidly on the NMR timescale. A single *tert*‐butyl group at the *ortho* position is enough to impart atropisomerism in anilides:^12^ the lack of conformational restriction in **6 b** demonstrates how much more flexible diarylamines are than the related anilides.

**Figure 2:**
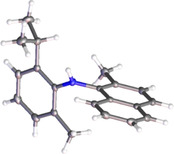
Crystal structure of **6 s**.

Information on the ground state conformers was also obtained by X‐ray crystallography. Crystals of diarylamines **6 c** and **6 s** were obtained by slow evaporation of a saturated solution in CH_2_Cl_2_.^31^ The structure of **6 s** (Figure 2) shows an apparently planar geometry at nitrogen, with a bond angle of 125° between the aromatic rings. The C−N−C plane is twisted by 47.6° with respect to the phenyl ring, and by 45.4° with respect to the naphthyl ring. The two rings are thus close to perpendicularity, with the orientation of the nitrogen's lone pair bisecting the dihedral angle between the two rings. The C−N bond lengths of 1.40 and 1.41 Å are similar to those of aniline,^32^ and comparable with those of atropisomeric diaryl ethers.^21^

**Figure 3:**
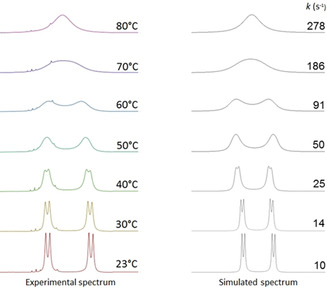
Variable‐temperature ^1^H NMR spectra of **6 g** in [D_6_]DMSO and the corresponding lineshape analysis.

Variable temperature ^1^H NMR was used with 2,2′,6‐tri‐substituted compounds **6 d**–**h** in order to determine the effect of temperature on lineshape close to the coalescence temperature (Table 1, Figures 3 and S1–7). The kinetic parameters for bond rotation (and consequently, interconversion of enantiomeric conformers) were then extracted by Eyring analysis.

**Table 1:** Barrier to rotation of the synthesised diarylamines.

Cpd	R^1^	R^2^	R^3^	R^4^	Δ*H* ^≠^ [kJ mol^−1^]	Δ*S* ^≠^ [J mol^−1^ K^−1^]	Δ*G* ^≠[a]^ [kJ mol^−1^]	*k* ^[a]^ [s^−1^]	*t*_1/2_^[a,b]^
**6 a**	2‐*i*Pr	H	2‐Me	H	–	–	–	–	–
**6 b**	2‐*t*Bu	H	2‐*i*Pr	H	–	–	–	–	–
**6 c**	2‐*i*Pr	H	2‐Me	6‐Me	–	–	–	–	–
**6 d**	2‐Et	6‐Me	2‐*i*Pr	H	58.3	−21.4	65.7^[c]^	28.5	12 ms
**6 e**	2‐*i*Pr	6‐Me	2‐Me	H	65.0	7.5	62.8^[c]^	62.2	5.6 ms
**6 f**	2‐*i*Pr	6‐Me	2‐*i*Pr	H	49.6	−53.7	65.8^[c]^	19.2	18 ms
**6 g**	2‐*i*Pr	2‐*i*Pr	2‐Me	H	51.0	−54.8	67.3^[d]^	9.9	35 ms
**6 h**	2‐*i*Pr	2‐*i*Pr	2‐*i*Pr	H	64.5	−21.0	70.7^[c]^	2.5	140 ms
**6 i**	2‐Et	6‐Me	2‐*t*Bu	H	53.2	−109.6	85.8^[e]^	5.6×10^−3^	1.0 min
**6 j**	2‐*i*Pr	6‐Me	2‐*t*Bu	H	51.8	−121.5	88.0^[f]^	2.3×10^−3^	2.5 min
**6 k**	2‐*i*Pr	2‐*i*Pr	2‐*t*Bu	H	–	–	–	–	–
**6 l**	2,4‐*t*Bu	H	2,3‐benzo^[g]^	6‐Me	–	–	–	–	–
**6 m**	2,4‐*t*Bu	6‐Me	2‐Me	H	37.9	−166.7	87.6^[f]^	2.8×10^−3^	2.1 min
**6 n**	2,4‐*t*Bu	6‐Me	2‐*i*Pr	H	40.8	−165.9	89.9^[f]^	1.1×10^−3^	5.3 min
**6 o**	2,4‐*t*Bu	6‐Me	2‐Ph	H	45.0	−151.6	90.2^[f]^	9.8×10^−4^	5.9 min
**6 p**	2,4‐*t*Bu	6‐Me	2,4‐*t*Bu	H	–	–	–	–	–
**6 q**	2‐Et	6‐Me	2,3‐benzo^[g]^	6‐Me	–	–	–	–	–
**6 r**	2‐*i*Pr	6‐Me	2,4‐*t*Bu	6‐Me	–	–	–	–	–
**6 s**	2‐*i*Pr	6‐Me	2,3‐benzo^[g]^	6‐Me	n/a	n/a	93.9^[h]^	2.1×10^−4^	27.3 min
**6 t**	2,4‐*t*Bu	6‐Me	2,3‐benzo^[g]^	6‐Me	n/a	n/a	130.1^[h,i]^	4.7×10^−6[i]^	20.3 h^[i]^ (122 years^[j]^)

[a] Values at 25 °C unless stated otherwise. [b] Half‐life for racemisation. [c] Calculated by lineshape analysis in [D_8_]toluene. [d] Calculated by lineshape analysis in [D_6_]DMSO. [e] Calculated by VT‐HPLC in hexane/ethyl acetate. [f] Calculated by VT‐HPLC in hexane/*iso*‐propanol. [g] 2,3‐benzo=1‐naphthyl. [h] Calculated by decay of enantiomeric excess in toluene. [i] At 100 °C. [j] At 25 °C assuming Δ*S* ^≠^=0.

Unless one of these three *ortho* substituents is a *tert*‐butyl group, half‐lives for racemisation of the enantiomeric conformers are typically of the order of milliseconds (Table 1). Changes in the size of the substituents from methyl to ethyl to isopropyl have a small but measurable effect on the barrier to rotation. Replacement of an ethyl group by an isopropyl group has little effect (**6 d** vs. **6 f**, **6 i** vs. **6 j**), but replacing a methyl group by an isopropyl group results in a three‐fold increase in the half‐life for racemisation (**6 e** vs. **6 f**).

Symmetrical compounds **6 g** and **6 h** likewise exhibit a similar difference. We previously observed that diaryl ethers with a ring symmetrically substituted at the 2,6‐position racemise much more rapidly owing to geared rotation.^21^ However, this was not the case for secondary diarylamines. In fact, regioisomers **6 e** and **6 g**, as well as **6 f** and **6 h**, undergo a ten‐fold decrease in their rate of interconversion upon introduction of symmetry in one of the rings.

**Figure 4:**
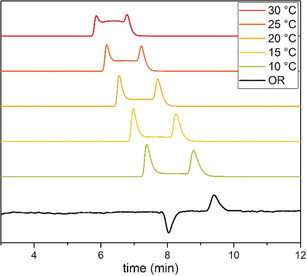
VT‐HPLC of **6 m**. OR=Optical rotation (at 10 °C).

A single *tert*‐butyl substituent on a tri‐*ortho*‐substituted compound dramatically increases the barrier to interconversion of the conformers, allowing the two enantiomers to be at least partially resolved at room temperature by HPLC on a chiral stationary phase. These compounds (such as **6 i**, **6 j**, and **6 n**) did not exhibit coalescence of NMR signals even at elevated temperature (up to 100 °C), so variable‐temperature HPLC (VT‐HPLC) on a chiral stationary phase (CSP) was used to provide plateaued traces characteristic of molecules whose conformers interconvert on the timescale of elution (Figures 4 and S8–12).^33^ The standard definition of an atropisomer^34^ requires a half‐life for racemisation of 1000 s (17 min) at a given temperature, so these compounds are not quite atropisomers at room temperature, but become so at around 0 °C. The rate of interconversion of the enantiomeric atropisomers was calculated using the Trapp equation at various temperature and used in an Eyring analysis to obtain barrier to enantiomerisation at room temperature.^33^

Tri‐*ortho*‐substituted diarylamines with a *tert*‐butyl group on their most functionalized ring were hardly affected by the substitution pattern of their second ring. The relatively constant rate of interconversion of compounds **6 m**, **6 n** and **6 o** suggests that the *tert*‐butyl group is the main influence on the half‐life of the compounds. Indeed, replacing a methyl by an isopropyl group changes the half‐life for racemisation (**6 m** vs. **6 n**) only by a factor of two, and no significant change in the rate of interconversion is observed with a planar (hence less bulky) phenyl ring substituent in compound **6 o**.

**Figure 5:**
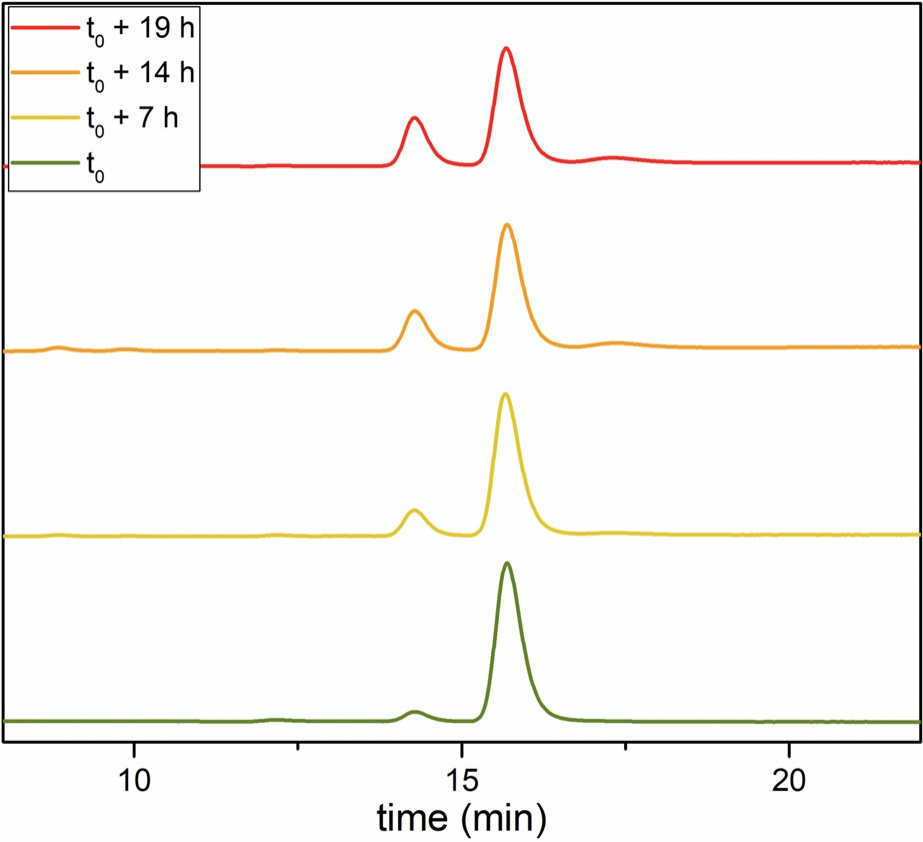
Decay of the enantiomeric ratio of **6 t** at 100 °C in toluene.

Introducing the most sterically hindered group on the less substituted ring makes the barrier to enantiomerisation more sensitive to substituent modification: an alteration as small as replacing an ethyl group for an isopropyl group, for example, doubles the half‐life for racemisation (compounds **6 i** vs. **6 j**). More importantly, a measurable difference in conformational stability between regioisomers **6 j** and **6 n** shows the importance of the relative position of the substituents in the design of atropisomeric diarylamines. CSP‐HPLC indicated that tetra‐*ortho*‐substituted diarylamines exist as stable, atropisomeric enantiomers that interconverted too slowly for analysis by VT‐HPLC. Instead, small samples of resolved and configurationally stable enantiomers were obtained by semi‐preparative CSP‐HPLC. The decay of their enantiomeric excess was followed over time (Figures 5 and S13,14) at a given temperature. While enantiomers of compounds **6 l** and **6 q** could not be separated, compounds **6 r** and **6 p**, with a *tert*‐butyl group on each ring, were well resolved, even at 40 °C. However, their poor solubility in the eluent mixture, as well as the reduced peak separation of their enantiomers, made isolation of an enantioenriched sample impossible. The absence of a plateaued chromatograph at 40 °C, though, suggests that the barriers to conformational interconversion in these compounds are high enough for compounds **6 r** and **6 p** to be conformationally stable at room temperature, and hence constitute atropisomers. These results indicate that a single *tert*‐butyl group is sufficient to allow a tetra‐*ortho*‐substituted diarylamine to exhibit atropisomerism, and that even tri‐*ortho*‐substituted diarylamines can exist as configurationally stable enantiomers (“near‐atropisomers”^29^) if each ring possesses an *ortho tert*‐butyl group.

Enantiomers of compounds **6 s** and **6 t** were resolved, and their barrier to rotation is high enough to observe atropisomerism at room temperature. While the barrier to interconversion of **6 s** was just high enough for it to be atropisomeric,^34^ it racemised within hours at room temperature. Compound **6 t**, however, was configurationally stable indefinitely at room temperature. Its racemisation was followed at 100 °C in toluene (Figure 5), at which it still displayed a half‐life for racemisation of around 20 hours.

The combination of a sterically hindered *tert*‐butyl group, together with the rigid *ortho*‐methyl‐1‐naphthalene, leads to a dramatic increase of the barrier to rotation, demonstrating that secondary diarylamines can be atropisomers even without using intramolecular non‐covalent bonding interactions to slow bond rotation.

Comparison with the barriers to rotation of the analogous diaryl ethers^21^ suggests that a similar substitution pattern incurs a higher barrier to rotation in an ether than it does in an amine, with the degree of difference depending significantly on the location of the steric bulk (see Supporting Information).

**Figure 6:**
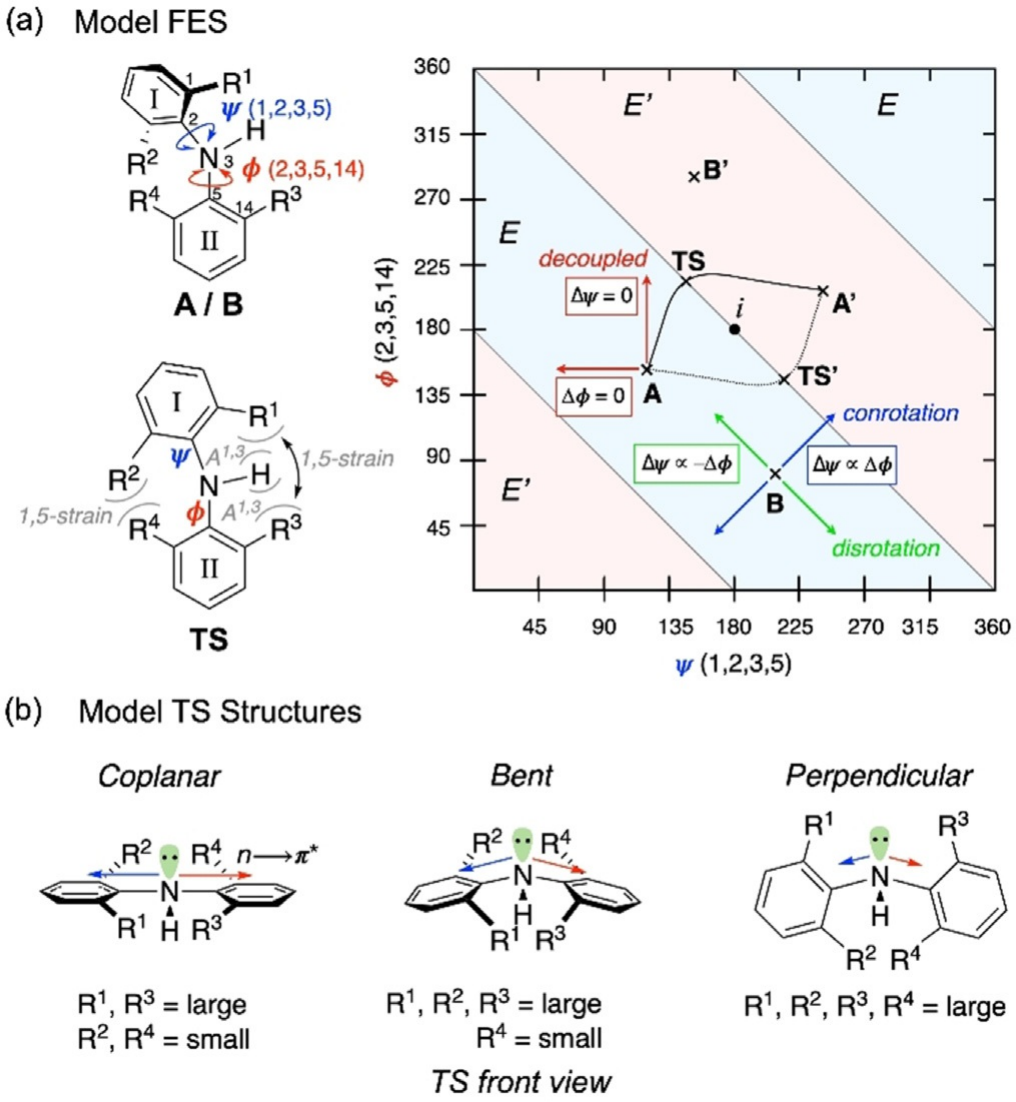
a) Model free energy surface (FES) as a function of the dihedral angles *ψ* and *φ*. Red arrows show decoupled rotation (Δ*ψ* or Δ*φ*=0), blue arrows show conrotatory motion (*ψ*∝*φ*), and green arrows show disrotatory motion (*ψ*∝−*φ*) of the two aryl groups. b) Three transition state models in operation depending on the size of the *ortho*‐substituents on each aryl group.

To gain further insight into the mechanism of racemisation, and the detailed influence of steric demand on the barriers to conformational interconversion, we turned to computation, using a combination of classical MD metadynamics, density‐functional theory (DFT), and coupled cluster calculations (see the Supporting Information for details). Free energy surfaces (FESs) in explicit toluene solvent were generated for three diarylamines with two, three or four *ortho*‐substituents (**6 a**, **6 j**, and **6 t**, respectively). The torsional angles ψ (atoms 1, 2, 3 and 5) and φ (atoms 2, 3, 5 and 14), defining the chiral axis, were selected as collective variables (CVs, Figure 6 a).^35^ Subsequently, different isomerization paths identified in the FES were further analysed, and the minima and transition states (TSs) calculated at the DLPNO‐CCSD(T)/def2‐TZVPP//M06‐2X/6–31+G* level of theory in implicit toluene solvent.^36^, ^37^, ^38^ A full characterisation of competing pathways is shown in the Supporting Information (Figure S17–S20). The delocalisation energy of the nitrogen lone pair was calculated using second‐order perturbation analysis (Δ*E* ^(2)^) of the Fock matrix obtained using NBO.

While the barrier to enantiomerisation was found to depend on the steric requirements set by the substituents (see below), the mechanism through which this process occurs can be explained by a general FES, as shown in Figure 6 a for a representative simplified diarylamine (for a detailed discussion of the general isomerisation model using FES, see Figure S16). In this model, the pale blue and red bands represent “valleys” in the FES associated with the enantiomers *E* and *E*′, with the local minima **A** and **B** being conformers of enantiomer *E*, and local minima **A′** and **B′** conformers of enantiomer *E*′. The paired enantiomers **A**/**A′**, **B**/**B′** and **TS**/**TS′** are related by inversion through the point *i*. Interconversion of the enantiomers requires that the two aryl groups move past one another at the TS, accompanied by inversion of configuration at the nitrogen centre. This can be achieved either through an *achiral* (at the point *i*; *ψ*=180°, *φ*=180°) or a *chiral* TS (solid and dashed black line). The trajectory taken to and from the TS can be further classified depending on the concertedness of the rotation of the two aryl rings: conrotatory rotation (Δ*ψ*∝Δ*φ*, blue arrows), disrotatory rotation (Δ*ψ*∝−Δ*φ*, green arrows); or decoupled rotation of a single aryl group (Δ*ψ* or Δ*φ*=0, red arrows). It is worth noting that disrotatory rotation, representing geared motion of the two aromatic rings, cannot alone lead to interconversion of enantiomers (for more details see Figure S16).^39^

The preference for a given mechanism is determined by five key interactions at the TS (Figure 6 a): two destabilising pseudo‐*syn*‐pentane interactions (1,5‐strain) between pairs **R^2^** and **R^4^**, and **R^1^** and **R^3^**; two 1,3‐allylic strain interactions (A^1,3^) between **R^1^** and N−H, and **R^3^** and N−H; and finally, a favourable electronic effect arising from delocalisation of nitrogen lone pair into the two aryl groups (*n*→π*). Bulkier substituents are expected to increase the barrier to rotation due to increase in strain and reduction of the nitrogen lone pair delocalisation.

With these elements in hand, three different scenarios can be postulated (Figure 6 b). When **R^2^** and **R^4^** are small, the preferred mechanism is that going through a *coplanar* achiral TS (Figure 6 b, left), with **R^2^** and **R^4^** placed in proximity to minimise 1,5‐steric repulsion while maximising *n*→π* delocalisation into both aryl rings. Increasing the size of **R^2^** increases 1,5‐steric repulsion between **R^2^** and **R^4^**, forcing a reduction in the distance between **R^1^**, **R^3^** and N−H, which increases 1,5‐steric repulsion and A^1,3^ strain. Relief of these interactions can occur by bending the two aryl rings out of the plane of the N−H bond. In this *bent* TS (Figure 6 b, centre), the extent of the bending is a balance between minimisation of steric repulsion and the loss of *n*→π* delocalisation. When all four aryl groups are large, a perpendicular TS is favoured, in which a combination of significant 1,5‐steric clashing between both the **R^1^**/**R^3^** and **R^2^**/**R^4^** pairs of *ortho*‐substituents and 2×A^1,3^ interactions require the aryl rings to be rotated such that they are both perpendicular to the N−H bond, forfeiting n→π* delocalisation (Figure 6 b, right). The smaller pair of *ortho*‐substituents pass one another more closely.

**Figure 7:**
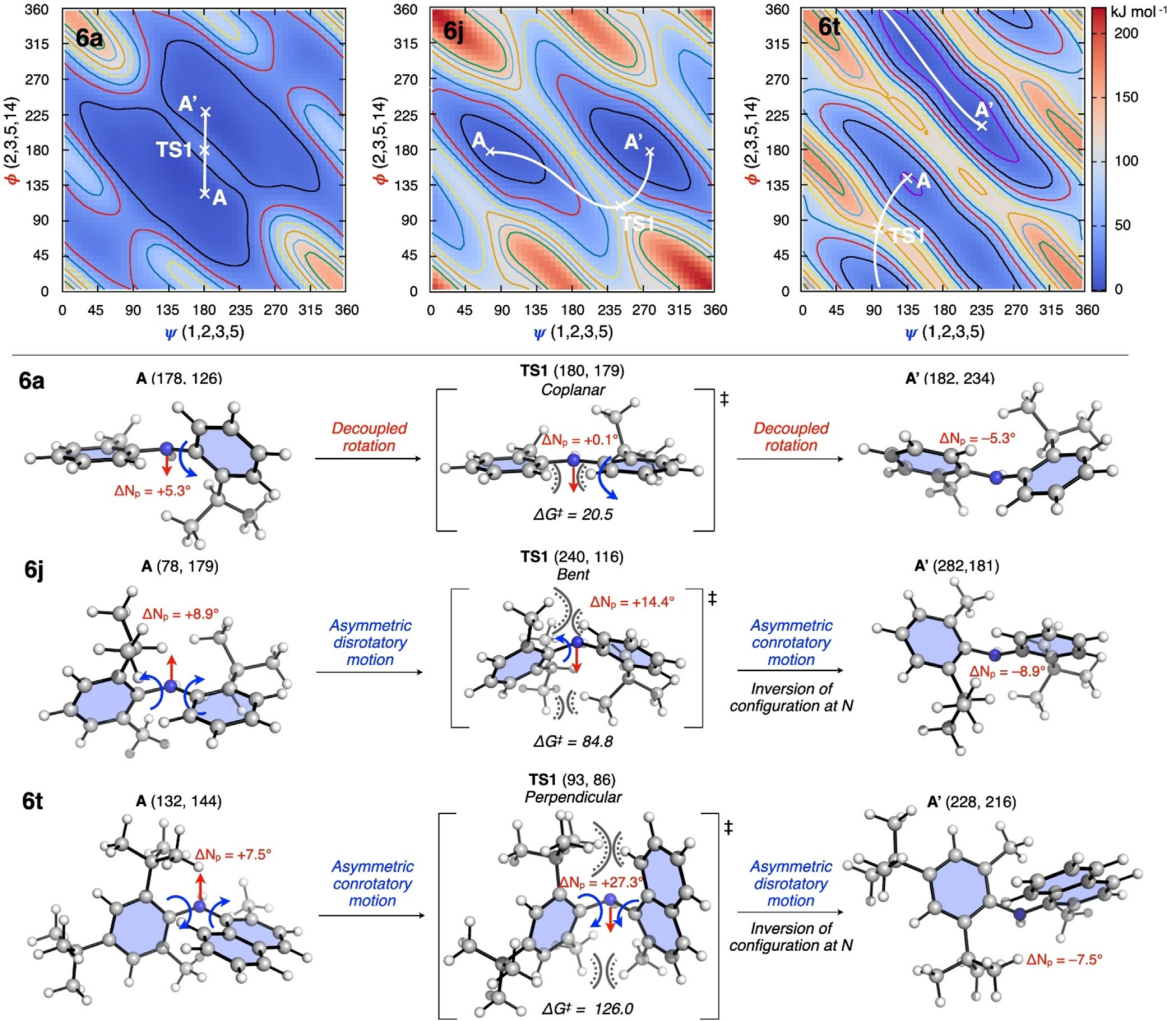
Classical FES with the preferred reaction pathway (solid white lines) for the isomerisation of diarylamines **6 a**, **6 j** and **6 t**. Optimised minima and TSs (coordinates on FES in parentheses), nitrogen pyramidalization (Δ*N*_p_) and activation free energies (in kJ mol^−1^) at the SMD(toluene)‐DLPNO‐CCSD(T)/def2‐TZVPP//SMD(toluene)‐M06‐2X/6–31+G* level of theory.

The pathways found for **6 a**, **6 j** and **6 t** represent archetypical examples of these three scenarios. For **6 a**, possessing only two *ortho*‐substituents, the lowest energy conformer has one aryl group fully conjugated with the nitrogen lone pair, while the other is slightly twisted out of the plane. Isomerisation takes place through a *coplanar* TS with the two aryl groups and the N−H bond lying in the same plane (Figure 7, **6 a**). The motion of each aryl group is decoupled (Δ*ψ*=2°, Δ*φ*=53°), such that only the C−N bond of the less conjugated *ortho*‐isopropyl aryl group rotates in this process. The mild steric penalty of bringing the two *ortho*‐hydrogen atoms together is compensated by an increase in N lone pair delocalisation at the TS (Δ*E* ^(2)^=+152 kJ mol^−1^ relative to the ground state and nitrogen pyramidalization ΔN_p_=+0.1°), leading to facile isomerisation.

Adding a third *ortho*‐substituent, such as in **6 j** (a *tert*‐butyl group) leads to a significant increase in the barrier to isomerisation. This arises from the increased steric repulsion between the new *ortho*‐substituent and the remaining *ortho*‐hydrogen, which disfavours a *coplanar* TS. Instead, isomerisation takes place through a *bent* TS, where the two aryl groups move in a disrotatory manner (Δ*ψ*=162°, Δ*φ*=−63°), with the two aryl rings facing each other while increasing the nitrogen pyramidalization (Δ*N*_p_) by 5.5° to minimise the loss of nitrogen lone pair delocalisation (Δ*E* ^(2)^=−45 kJ mol^−1^). Only after the TS does inversion of configuration occur at the nitrogen centre. The increased steric demand and reduction of electronic stabilisation at the TS lead to a barrier of 84.8 kJ mol^−1^, in good agreement with the experimental value (Table 1).

Introduction of a fourth *ortho*‐substituent, as in **6 t**, increases the barrier to isomerisation further. In the ground state, and similar to the arrangement observed in the crystal structure of **6 s**, the two rings are approximately perpendicular to one another, with the nitrogen lone pair almost exclusively delocalised into the naphthyl group (Figures 2 and 7, **6 t**, and Figure S19). To achieve isomerisation, the two aryl groups must first undergo concerted conrotation (Δ*ψ*=39°, Δ*φ*=58°), sacrificing nitrogen lone pair delocalisation in the process (Δ*E* ^(2)^=−150 kJ mol^−1^, Δ*N*_p_=+27.3°). This leads to a *perpendicular* TS, in which both aryl groups lie in planes perpendicular to the C−N−C plane. To move past each other, and minimise 1,5‐steric repulsion between the two pairs of *ortho*‐substituents, both aryl groups are distorted, further increasing the TS energy. Finally (and similarly to **6 j**) inversion of configuration at nitrogen occurs after the TS, accompanied by disrotatory motion of the two aryl rings to return the naphthyl group to its original position.

**Figure 8:**
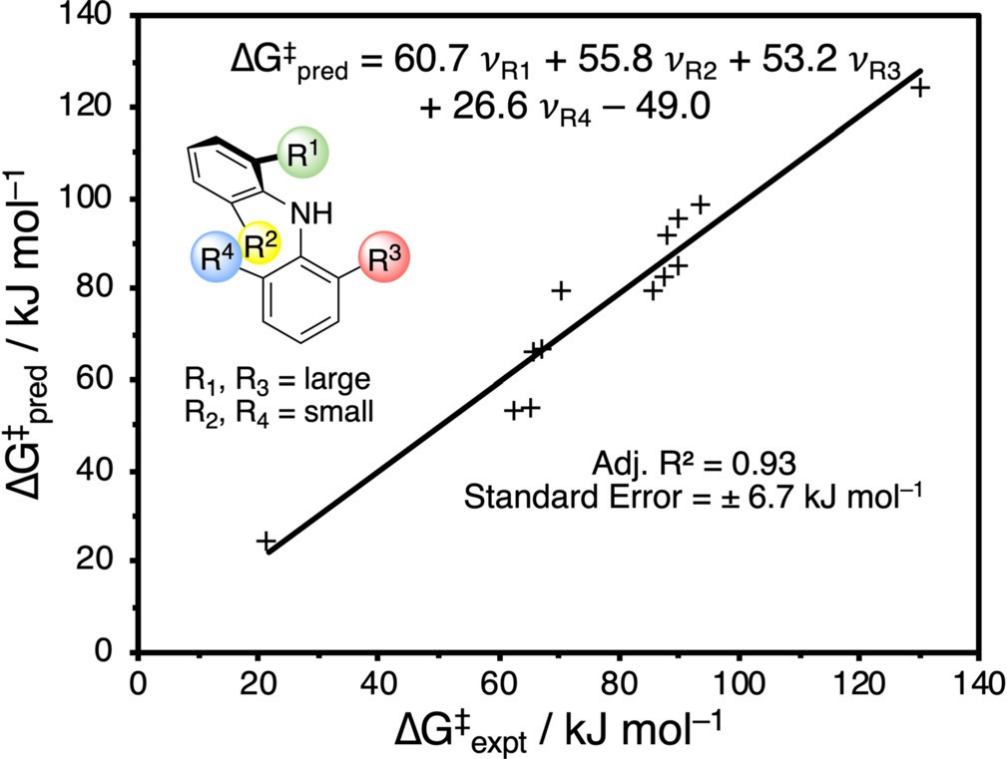
Linear free energy relationship between the predicted and experimental barriers to isomerisation using Charton steric parameters (ν_R*i*_, *i*=1–4) for each *ortho*‐substituent.

It is interesting to note that while adding a fourth *ortho*‐substituent increases the barrier, the consequence is less pronounced than adding a third *ortho*‐substituent to a di‐*ortho*‐substituted diarylamine, suggesting that the size of the fourth substituent has a smaller influence on the barrier to isomerisation than the other three. This result is supported by a linear free energy relationship (LFER) between the barrier to isomerisation and the Charton steric parameters for the four *ortho*‐substituents (Figure 8, Table S2), where R^1^ is the largest substituent overall, and R^3^ is the largest substituent on the second ring.^40^, ^41^ A strong correlation (adjusted R^2^=0.93) suggests that the barrier is almost entirely dominated by steric effects, and that the three TS models lie on a continuum where *coplanar, bent* and *perpendicular* geometries are archetypal representations. This empirical model confirms that the fourth substituent has a smaller influence on the barrier compared to the others, and provides a simple approach to the prediction of configurational stability in novel diarylamines.

**Figure 9:**
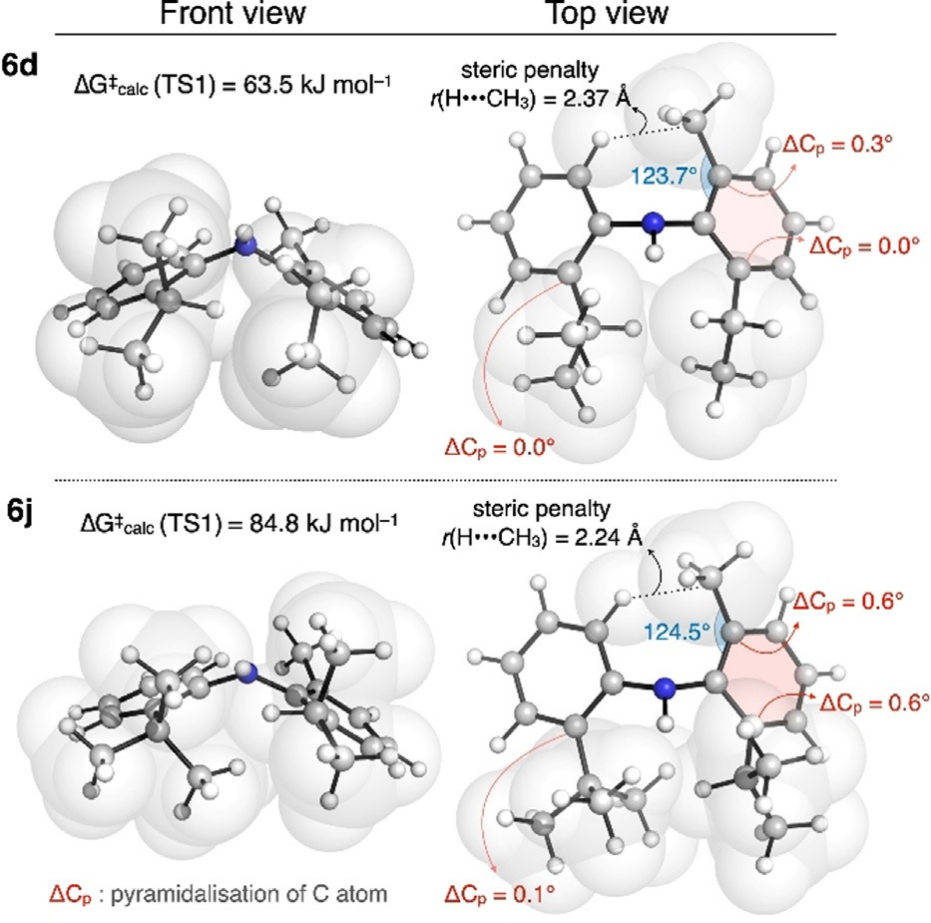
Comparison of lowest energy TS1 structures for **6 d** and **6 j** calculated at the SMD(toluene)‐DLPNO‐CCSD(T)/def2‐TZVPP//SMD(toluene)‐M06‐2X/6–31+G* level of theory.

Among the substituents studied in this work, a single *tert*‐butyl group was found to have a critical effect on the interconversion barrier. This effect can be better understood by comparing the lowest energy TS for the isomerisation of **6 d** and **6 j** (Figure 9), where the ethyl/isopropyl groups in **6 d** are replaced by isopropyl/*tert*‐butyl groups in **6 j**. In the former, steric clashes are minimised by positioning the hydrogen atom of the isopropyl group towards the *ortho*‐substituent. However, in **6 j**, this is not possible, so the *tert*‐butyl group must rotate to avoid pointing a methyl group directly towards the opposite *ortho*‐substituent. This increased *tert*‐butyl/isopropyl 1,5‐steric clash also causes these two *ortho*‐substituents to move apart, forcing an increase in the 1,5‐steric clash between the second pair of *ortho*‐substituents (H and CH_3_), where *r*(H⋅⋅⋅CH_3_) is reduced by 0.13 Å in **6 j** relative to **6 d**. To alleviate some of the resultant 1,5‐steric repulsion, the substituents distort out of the plane of the aryl ring, leading to greater pyramidalization of the *ortho*‐carbon atoms (ΔC_p_) in **6 j** than **6 d**. The combination of these factors results in a significantly increased barrier to interconversion in **6 j** over **6 d**, despite the small difference in structure.

The basic amino group of **6 a**–**u** has the potential to be involved in hydrogen bonding, suggesting that the rate of conformational interconversion might be solvent‐dependent. The barrier of rotation of **6 d** was calculated in a variety of solvents by line‐shape analysis of VT ^1^H NMR spectra. We chose non‐polar [D_8_]toluene, polar protic [D_6_]ethanol, and polar aprotic [D_3_]acetonitrile (Table 2, Figures S1–3). In all solvents, the barrier to rotation is comparable, with an error of ±0.7 kJ mol^−1^, indicating that polarity has little to no effect on the barrier to rotation.

**Table 2:** Solvent effects on the barrier to interconversion of **6 d**.

Solvent	Δ*H* ^≠^ [kJ mol^−1^]	Δ*S* ^≠^ [J mol^−1^ K^−1^]	Δ*G* ^≠[a]^ [kJ mol^−1^]
[D_8_]toluene^[b]^	41.5	−80.0	64.7
[D_8_]toluene^[c]^	58.3	−21.4	65.7
C_2_D_5_OD^[b]^	31.7	−114.0	65.7
C_2_D_5_OD^[c]^	35.8	−96.0	64.4
CD_3_CN^[b]^	31.9	−112.1	65.3
CD_3_CN^[c,d]^	–	–	–

[a] At 25 °C. [b] Calculated using the CH(C*H_3_*)_2_ signal. [c] Calculated using the CH_2_C*H_3_* signal. [d] Not determined due to peak overlap.

Sutherland and co‐workers,^42^ in their seminal work on the conformational restriction of anilides, showed that the planarity of the nitrogen and conjugation of its lone pair with a delocalized π‐system is necessary for high conformational stability. Kitagawa et al. also observed acceleration of the rate of interconversion in protonated *N*‐aryl tetrahydroquinolines.^43^

**Figure 10:**
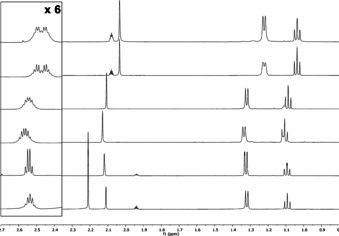
Line sharpening upon protonation of **6 d**. Top to bottom: [D_8_]toluene + TfOH; [D_8_]toluene; C_2_D_5_OD + TfOH; C_2_D_5_OD; CD_3_CN + TfOH; CD_3_CN.

To explore this feature in diarylamines, solutions of **6 d** in different deuterated solvents were treated with a strong acid. Because of the low basicity of diarylamines (p*K*_a_≈1 for diphenylamine^44^), triflic acid was used. ^1^H NMR spectra of the resulting solutions were recorded in [D_3_]acetonitrile, [D_6_]ethanol, and [D_8_]toluene. Excessive line broadening of the samples (probably due to precipitation) precluded lineshape analysis of protonated **6 d** at low temperature. However, in all three solvents, the isopropyl CH(*CH_3_*)_2_ signals centred around 1.22 ppm in [D_8_]toluene and 1.32 ppm in [D_3_]acetonitrile and [D_6_]ethanol became significantly sharper upon protonation, implying an acceleration of the rate of enantiomerisation (Figure 10).

Previous examples of atropisomerism in diarylamines relied either on a hydrogen bond between one of the rings and the secondary nitrogen atom,^17^, ^20^ or on the steric effect of *N*‐alkylation.^14^, ^15^ We also aimed to explore the conformational effect of alkylating the amine nitrogen in these simple diarylamines. Compound **6 g** could be methylated by treatment with sodium hydride and methyl iodide, and this *N*‐CH_3_ substituent increased the half‐life for racemisation of the product **6 g′** more than tenfold (Table 3). The increased enthalpy of activation, together with the low and negative entropy of activation, suggest that the transition state is destabilized by the strain caused by the methyl substituent in the vicinity of the aryl ring. **6 g** was unreactive towards any other electrophile, and attempted *N*‐methylation of the other diarylamines with an excess of base and alkylating agent in refluxing THF yielded no product, showing that the nitrogen atom is buried within the sterically demanding groups at the *ortho*‐positions.

**Table 3:** Effect of *N*‐methylation on the barrier to interconversion of **6 g**. 
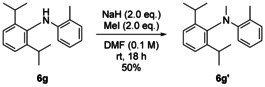

Cpd	Δ*H* ^≠^ [kJ mol^−1^]	Δ*S* ^≠^ [J mol^−1^ K^−1^]	Δ*G* ^≠[a]^ [kJ mol^−1^]	*k* [s^−1^]	*t*_1/2_^[a]^ [s]
**6 g**	53.4	−47.7	67.6	8.9	0.039
**6 g′**	71.7	−7.80	74.0	0.67	0.52

[a] At 25 °C.

## Conclusion

Sterically hindered, secondary diarylamines may display atropisomerism. While 2,2′‐di‐*ortho*‐substituted diarylamines interconvert too rapidly for individual conformers to be detected by ^1^H NMR at low temperature, introduction of a third sterically demanding substituent decreases the rates of interconversion enough for the barriers to enantiomerisation to be calculated by NMR lineshape analysis. A single *tert*‐butyl group can have a critical effect on the rate of enantiomerisation, as tri‐*ortho*‐substituted diarylamines in which one of the substituents is *tert*‐butyl may be separated by HPLC on chiral stationary phase. However, racemisation at room temperature precludes their isolation in enantiopure form. Finally, four *ortho* substituents lead to conformational stability at room temperature, particularly when one of them is a *tert*‐butyl group. Diarylamine **6 t** was the most congested compound prepared, displaying a barrier to rotation of up to 130.1 kJ mol^−1^ and a half‐life of racemisation of 20 hours at 100 °C, or approximately 122 years at room temperature.

Computation was used to probe the mechanism of isomerisation. A simple model based on the steric requirements imposed by substituents allows the prediction of the pathways by which interconversion takes place, where *coplanar, bent* and *perpendicular* TS geometries were found to be archetypal scenarios experimentally illustrated by **6 a**, **6 j**, and **6 t**, respectively. A simple LFER based on Charton parameters was found to accurately predict the configurational stability of novel diarylamines. We expect this information will be of value in the design of chiral atropisomeric ligands and of potential medicinal chemistry targets.

## Conflict of interest

The authors declare no conflict of interest.

## Supporting information

As a service to our authors and readers, this journal provides supporting information supplied by the authors. Such materials are peer reviewed and may be re‐organized for online delivery, but are not copy‐edited or typeset. Technical support issues arising from supporting information (other than missing files) should be addressed to the authors.

Supplementary

## References

[1] D. Parmar , E. Sugiono , S. Raja , M. Rueping , Chem. Rev. 2014, 114, 9047–9153. 25203602 10.1021/cr5001496

[2] S. Sakane , J. Fujiwara , K. Maruoka , H. Yamamoto , J. Am. Chem. Soc. 1983, 105, 6154–6155.

[3] T. D. James , K. R. A. Samankumara Sandanayake , S. Shinkai , Nature 1995, 374, 345–347.

[4] J. Clayden , Angew. Chem. Int. Ed. Engl. 1997, 36, 949–951;

[5] E. Kumarasamy , R. Raghunathan , M. P. Sibi , J. Sivaguru , Chem. Rev. 2015, 115, 11239–11300. 26414162 10.1021/acs.chemrev.5b00136

[6] S. Brandes , B. Niess , M. Bella , A. Prieto , J. Overgaard , K. A. Jørgensen , Chem. Eur. J. 2006, 12, 6039–6052. 16789058 10.1002/chem.200600495

[7] S. T. Toenjes , J. L. Gustafson , Future Med. Chem. 2018, 10, 409–422. 29380622 10.4155/fmc-2017-0152 PMC5967358

[8] J. Clayden , W. J. Moran , P. J. Edwards , S. R. Laplante , Angew. Chem. Int. Ed. 2009, 48, 6398–6401; 10.1002/anie.200901719 19637174

[9] G. Bringmann , S. Tasler , H. Endress , K. Messer , M. Wohlfarth , W. Lobin , D.- Wu , J. Am. Chem. Soc. 2001, 123, 2703–2712. 11456955 10.1021/ja003488c

[10] G. Bringmann , T. Gulder , M. Reichert , F. Meyer , Org. Lett. 2006, 8, 1037–1040. 16524262 10.1021/ol052946p

[11] A. A. Kanakis , V. Sarli , Org. Lett. 2010, 12, 4872–4875. 20886840 10.1021/ol102035s

[12] D. P. Curran , G. R. Hale , S. J. Geib , A. Balog , Q. B. Cass , A. L. G. Degani , M. Z. Hernandes , L. C. G. Freitas , Tetrahedron: Asymmetry 1997, 8, 3955–3975.

[13] J. Clayden , H. Turner , M. Helliwell , E. Moir , J. Org. Chem. 2008, 73, 4415–4423. 18399672 10.1021/jo702706c

[14] R. Costil , H. J. A. Dale , N. Fey , G. Whitcombe , J. V. Matlock , J. Clayden , Angew. Chem. Int. Ed. 2017, 56, 12533–12537; 10.1002/anie.201706341 28817222

[15] Y. Iwasaki , R. Morisawa , S. Yokojima , H. Hasegawa , C. Roussel , N. Vanthuyne , E. Caytan , O. Kitagawa , Chem. Eur. J. 2018, 24, 4453–4458. 29363203 10.1002/chem.201706115

[16] G. Furukawa , T. Shirai , Y. Homma , E. Caytan , N. Vanthuyne , D. Farran , C. Roussel , O. Kitagawa , J. Org. Chem. 2020, 85, 5109–5113. [Note added in proof: an asymmetric synthesis of N-arylindolines has recently been reported by Wencel-Delord and coworkers:32138515 10.1021/acs.joc.0c00284

[17] T. Kawabata , C. Jiang , K. Hayashi , K. Tsubaki , T. Yoshimura , S. Majumdar , T. Sasamori , N. Tokitoh , J. Am. Chem. Soc. 2009, 131, 54–55. 19093814 10.1021/ja808213r

[18] K. Hayashi , N. Matubayasi , C. Jiang , T. Yoshimura , S. Majumdar , T. Sasamori , N. Tokitoh , T. Kawabata , J. Org. Chem. 2010, 75, 5031–5036. 20586407 10.1021/jo100586b

[19] K. Hayashi , Y. Nakajima , F. Ozawa , T. Kawabata , Chem. Lett. 2010, 39, 643–645.

[20] S. D. Vaidya , S. T. Toenjes , N. Yamamoto , S. M. Maddox , J. L. Gustafson , J. Am. Chem. Soc. 2020, 142, 2198–2203. 31944689 10.1021/jacs.9b12994 PMC7239344

[21] M. S. Betson , J. Clayden , C. P. Worrall , S. Peace , Angew. Chem. Int. Ed. 2006, 45, 5803–5807; 10.1002/anie.200601866 16874829

[22] J. Clayden , C. P. Worrall , W. J. Moran , M. Helliwell , Angew. Chem. Int. Ed. 2008, 47, 3234–3237; 10.1002/anie.200705660 18348136

[23] J. Clayden , J. Senior , M. Helliwell , Angew. Chem. Int. Ed. 2009, 48, 6270–6273; 10.1002/anie.200901718 19610006

[24] T. Mino , Y. Tanaka , Y. Hattori , T. Yabusaki , H. Saotome , M. Sakamoto , T. Fujita , J. Org. Chem. 2006, 71, 7346–7353. 16958529 10.1021/jo061261f

[25] J. Clayden , S. P. Fletcher , J. Senior , C. P. Worrall , Tetrahedron: Asymmetry 2010, 21, 1355–1360.

[26] A. Lodola , S. Bertolini , M. Biagetti , S. Capacchi , F. Facchinetti , P. M. Gallo , A. Pappani , M. Mor , D. Pala , S. Rivara , F. Visentini , M. Corsi , A. M. Capelli , J. Med. Chem. 2017, 60, 4304–4315. 28489362 10.1021/acs.jmedchem.7b00247

[27] S. R. Laplante , P. J. Edwards , L. D. Fader , A. Jakalian , O. Hucke , ChemMedChem 2011, 6, 505–513. 21360821 10.1002/cmdc.201000485

[28] A. J. Fugard , A. S. K. Lahdenperä , J. S. J. Tan , A. Mekareeya , R. S. Paton , M. D. Smith , Angew. Chem. Int. Ed. 2019, 58, 2795–2798; 10.1002/anie.201814362 PMC6492105 30644159

[29] J. Clayden , Chem. Commun. 2004, 127–135. 10.1039/b307976g 14737515

[30] S. M. Raders , J. N. Moore , J. K. Parks , A. D. Miller , T. M. Leißing , S. P. Kelley , R. D. Rogers , K. H. Shaughnessy , J. Org. Chem. 2013, 78, 4649–4664. 23638733 10.1021/jo400435z

[31] Deposition Numbers 1842707 (for **6c**), and 1842708 (for **6s**) contain the supplementary crystallographic data for this paper. These data are provided free of charge by the joint Cambridge Crystallographic Data Centre and Fachinformationszentrum Karlsruhe Access Structures service www.ccdc.cam.ac.uk/structures.

[32] K. C. Gross , P. G. Seybold , Int. J. Quantum Chem. 2000, 80, 1107–1115.

[33] O. Trapp , Anal. Chem. 2006, 78, 189–198. 16383327 10.1021/ac051655r

[34] M. Ōki , Top. Stereochem. 1983, 1–81.

[35] A. Laio , M. Parrinello , Proc. Natl. Acad. Sci. USA 2002, 99, 12562–12566. 12271136 10.1073/pnas.202427399 PMC130499

[36] Y. Zhao , D. G. Truhlar , Theor. Chem. Acc. 2008, 120, 215–241.

[37] C. Riplinger , F. Neese , J. Chem. Phys. 2013, 138, 034106. 23343267 10.1063/1.4773581

[38] A. V. Marenich , C. J. Cramer , D. G. Truhlar , J. Phys. Chem. B 2009, 113, 6378–6396. 19366259 10.1021/jp810292n

[39] Similar mechanistic features were modelled at a much lower level of theory for the analogous ethers: see ref. [21].

[40] M. Charton , J. Am. Chem. Soc. 1975, 97, 1552–1556.

[41] M. Charton , J. Org. Chem. 1976, 41, 2217–2220.

[42] B. J. Price , J. A. Eggleston , I. O. Sutherland , J. Chem. Soc. B 1966, 88, 922–925.

[43] Y. Suzuki , M. Kageyama , R. Morisawa , Y. Dobashi , H. Hasegawa , S. Yokojima , O. Kitagawa , Chem. Commun. 2015, 51, 11229–11232. 10.1039/c5cc03659c 26077702

[44] J. Sangster , J. Phys. Chem. Ref. Data 1989, 18, 1111–1229.

