# Supplementary material for: Atropisomerism in Diarylamines: Structural Requirements and Mechanisms of Conformational Interconversion
Source: Angew Chem Int Ed Engl. 2020 Aug 20;59(42):18670–8. doi: 10.1002/anie.202007595 (PMC7589358; doi:10.1002/anie.202007595)
Supplement: Supplementary file 1 — Supplementary [file ANIE-59-18670-s001.pdf]

Angewandte Chemie
Eine Zeitschrift der Gesellschaft Deutscher Chemiker

Supporting Information

Atropisomerism in Diarylamines: Structural Requirements and Mechanisms of Conformational Interconversion

*Romain Costil, Alistair J. Sterling, Fernanda Duarte, and Jonathan Clayden**

anie_202007595_sm_miscellaneous_information.pdf

Supporting Information

Contents

General Directions 2

Synthetic procedures 3

Calculation of barriers to enantiomerisation 32

VT ^{1}H NMR experiments 32

Chiral VT-HPLC experiments 47

Decay of enantiomeric excess of enantioenriched samples 53

Comparison of barriers to interconversion with similar diaryl ethers. 56

Computational Details 57

MD Simulations 57

DFT Calculations 58

LFER Analysis 59

Isomerisation Pathways 61

Bibliography 74

^{1}H and ^{13}C NMR spectra of new compounds 75

General Directions

Reactions requiring anhydrous conditions were performed under nitrogen atmosphere in glassware which was flame-dried. Air- and moisture-sensitive liquids and solutions were transferred by syringe or cannula into the reaction vessels through rubber septa. All reagents were purchased at highest commercial quality and used as received. Non-anhydrous solvents were purchased (unless specified) at the highest commercial quality and used as received. Toluene was distilled over CaH_2 and stored under N_2. THF was distilled over Na using benzophenone as an indicator and stored under N_2. All solvents were removed *in vacuo* using a rotary evaporator. Petroleum ether indicates fractions of PE boiling at 40-60 °C. Anhydrous THF was distilled over sodium using benzophenone as an indicator. Toluene was distilled over CaH_2.

Nuclear Magnetic Resonance (NMR) spectra (^1H and ^{13}C) were recorded on either Bruker Avance III 400 or 500 MHz spectrometers. The residual solvent peak was used as internal standards when assigning NMR spectra. Chemical shifts (δ) are quoted in parts per million (ppm) using $CDCl_3$ (^1H: δ = 7.26 ppm; ^{13}C: δ = 77.16 ppm) or DMSO-*d6* as a reference (^1H: δ = 2.50 ppm). Coupling constants (*J*) are reported to the nearest 0.1 Hz. The splitting patterns for the spectra assignment are abbreviated to singlet (s), doublet (d), triplet (t), quartet (q), septet (sept.), multiplet (m), broad (br.) and some as a combination of these. When possible, each signal was assigned to the corresponding atom.

IR spectra were recorded on neat compounds using a Perkin Elmer (Spectrum One) FT-IR spectrometer. Only strong and selected absorbance's (ν_{max} expressed in cm^{-1}) are reported.

High resolution mass spectra were recorded on a Thermo ORBITRAP Exactive Plus (APCI) mass spectrometer.

Capillary melting points were determined on a Stuart Scientific melting point SMP 10 apparatus.

Thin layer chromatography (TLC) was performed using commercially available pre-coated plates (Macherey-Nagel alugram SIL G/UV_{254}). Visualisation was by UV light (at 254 nm) or by staining with 'Seebach' dip (2.50 g phosphomolybdic acid hydrate, 1.00 g Cerium (IV) sulfate tetrahydrate, 3.2 mL conc. H_2SO_4, 90 mL H_2O) then heating. Flash column chromatography used chromatography grade silica, 60 Å particle size from Aldrich.

Synthetic procedures

General Procedure 1 (GP1), Buchwald–Hartwig coupling: A flame-dried microwave vial was allowed to cool to rt *in vacuo* and refilled with nitrogen. To this was added the required aniline (1.2 equiv.), aryl bromide (1.0 equiv.), NaO*t*Bu (1.25 equiv.), Pd_2dba_3 (1 mol%), PNp_3 (2 mol%) and distilled toluene (1.0 M). The reaction was allowed to stir at 80 °C for 16 h, cooled down to rt, filtered over Celite and concentrated *in vacuo* to yield the crude diarylamine. Purification by column chromatography yielded the desired product.

Trineopentyl phosphine – PNp_3

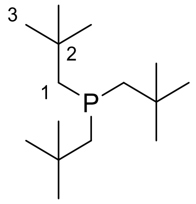

Following a literature procedure,[1] to a solution of neopentyl magnesium chloride in THF (1.0 M, 24 mL, 24 mmol, 4.5 equiv.) at 0°C was added dropwise freshly distilled phosphorus trichloride (0.46 mL, 5.27 mmol, 1.0 equiv.). The solution was refluxed for 5 h, allowed to cool to room temperature and stirred for 14 h. The mixture was concentrated *in vacuo*, and degassed diethyl ether was added, followed by a saturated aqueous solution of NH_4Cl. The aqueous layer was extracted with diethyl ether. Organics were combined, dried over $MgSO_4$ and concentrated *in vacuo*. The brown oil was distilled under vacuum (15 mbar, 150°C) and recrystallized in ethanol to afford PNp_3 as white crystalline needles (222 mg, 0.91 mmol, 17%),

m.p. 59°C.

δ^{1}H (400 MHz, $CDCl_3$) 1.35 (6 H, d, *J* = 3.7 Hz, H-1), 0.98 (27 H, s, H-3).

δ^{13}C (101 MHz, $CDCl_3$) 47.7 (d, *J* = 14.8 Hz, C-1), 32.0 (d, *J* = 13.8 Hz, C-2), 31.1 (d, *J* = 8.3 Hz, C-3).

Data consistent with that reported in the literature.[1]

1-bromo-2-(*tert*-butyl)benzene

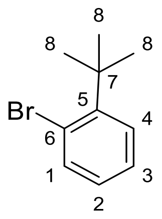

To a solution of *tert*-butylaniline (7.8 mL, 50 mmol, 1.0 equiv.) in water (10 mL) and HBr (48% aq., 12.5 mL, 110 mmol, 2.2 equiv.) at 0 °C was added slowly a solution of $NaNO_2$ (7.6 g, 110 mmol, 2.2 equiv.) in water (10 mL) while keeping the internal temperature between 5 and 10 °C. The mixture was stirred for 2 h and copper (0.20 g, 3.1 mmol, 6.2 mol%) was added and the mixture was cautiously heated to reflux. After 2 h, the mixture was diluted with water and extracted with diethyl ether. The organics were combined, washed with an aqueous solution of 10% NaOH, brine, dried over $MgSO_4$ and concentrated *in vacuo*. Purification by flash column chromatography (SiO_2, PE) yielded the title compound as a pink oil (3.2 g, 15 mmol, 30%).

δ^1H (400 MHz, $CDCl_3$) 7.60 (1 H, dd, *J* = 7.9 Hz, 1.4, H-4), 7.46 (1 H, dd, *J* = 8.0 Hz, 1.6, H-1), 7.26 (1 H, td, *J* = 7.6, 1.4 Hz, H-2), 7.05 (1 H, td, *J* = 7.6 Hz, 1.4, H-3), 1.53 (9 H, s, H-8).

δ^{13}C (101 MHz, $CDCl_3$) 147.7 (C-5), 135.8 (C-4), 128.0 (C-1), 127.5 (C-3), 127.3 (C-2), 122.7 (C-6), 36.6 (C-7), 29.7 (C-8). Data consistent with that reported in the literature.[2]

2-bromo-1,5-di-*tert*-butyl-3-methylbenzene

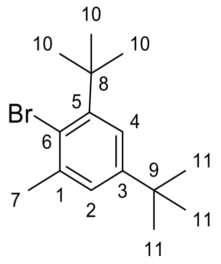

To a solution of 3,5-di-*tert*-butyltoluene (2.5 g, 12.2 mmol, 1.0 equiv.) and iron (0.15 g, 2.7 mmol, 22 mol%) in CCl_4 (25 mL) at 0°C was added a solution of bromine (0.68 mL, 13.3 mmol, 1.1 equiv.) in CCl_4 (6 mL). The mixture was stirred for 2 h, filtered, washed with a saturated aqueous solution of $NaHCO_3$, brine, dried over Na_2SO_4 and concentrated *in vacuo*. The oil was then purified by distillation (4.9 mbar, 120°C) to yield the title compound as a colourless oil (1.5 g, 5.3 mmol, 43%).

δ^{1}H (400 MHz, $CDCl_3$) 7.40 (1 H, d, *J* = 2.4 Hz, H-4), 7.21 (1 H, d, *J* = 2.4 Hz, H-2), 2.51 (3 H, s, C-7), 1.62 (9 H, s, H-10), 1.37 (9 H, s, H-11).

δ^{13}C (101 MHz, $CDCl_3$) 149.3 (C-3), 147.4 (C-5), 139.4 (C-1), 126.1 (C-2), 123.0 (C-4), 122.8 (C-6), 37.4 (C-9), 34.7 (C-8), 31.5 (C-10), 30.2 (C-11), 25.89 (C-7).

Data consistent with that reported in the literature.[3]

1,5-di-*tert*-butyl-3-methyl-2-nitrobenzene

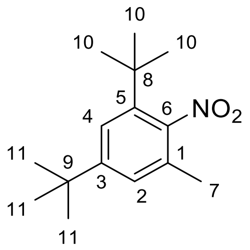

To a solution of 3,5-di-*tert*-butyltoluene (5.2 g, 25.4 mmol, 1.0 equiv.) in acetic anhydride (4.3 mL) was added dropwise a mixture of HNO_3 (70% aq., 1.6 mL, 101 mmol, 4.0 equiv.), acetic acid (1.5 mL) and acetic anhydride (1.5 mL) while keeping the internal temperature below 20°C. The solution was then stirred at room temperature for 3 h and at 55°C for 10 min. The reaction mixture was poured on ice, filtered and recrystallized in ethanol, yielding the title compound as a pale-yellow solid (3.1 g, 12.2 mmol, 49%) together with 2.2 g of crude product.

δ^1H (400 MHz, $CDCl_3$) 7.37 (1 H, d, *J* = 1.9 Hz, H-4), 7.13 (1 H, d, *J* = 1.7 Hz, H-2), 2.23 (3 H, s, H-7), 1.38 (9 H, s, H-10), 1.31 (9 H, s, *J* 1.8, H-11).

δ^{13}C (101 MHz, $CDCl_3$) 152.3 (C-3), 149.1 (C-6), 139.6 (C-5), 129.2 (C-1), 126.1 (C-2), 122.9 (C-4), 36.0 (C-8), 35.0 (C-9), 31.3 (C-11), 31.1 (C-10), 17.8 (C-7).

Data consistent with that reported in the literature.[4]

2,4-di-*tert*-butyl-6-methylaniline

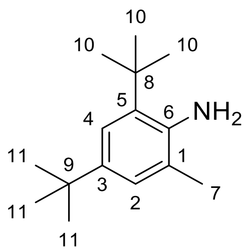

To a solution of 1,5-di-*tert*-butyl-3-methyl-2-nitrobenzene (1.0 g, 4.05 mmol) in THF (50 mL) was added portionwise $LiAlH_4$ (1.5 g, 40.5 mmol, 10.0 equiv.). The mixture was cautiously heated to reflux and stirred 16 h, then quenched with a saturated aqueous solution of $Na_2S_2O_3$. After filtration, the mixture was extracted with diethyl ether and concentrated *in vacuo*. The resulting oil was dissolved in a solution of HCl in methanol, concentrated *in vacuo* and triturated in diethyl ether. The pink solid was then suspended in an aqueous solution of 2 M NaOH, extracted with diethyl ether. Organics were combined, dried over Na_2SO_4, concentrated *in vacuo*, and filtration over a small plug of silica (PE/DCM 6040) yielded the title compound as a colourless oil (660 mg, 3.0 mmol, 75%).

δ^{1}H (400 MHz, $CDCl_3$) 7.19 (1 H, d, *J* = 1.6 Hz, H-4), 7.01 (1 H, d, *J* = 1.6 Hz, H-2), 3.75 (2 H, br. s,), 2.20 (3 H, s, H-7), 1.45 (9 H, s, H-11), 1.29 (9 H, s, H-10).

δ^{13}C (101 MHz, $CDCl_3$) 140.4 (C-6), 140.2 (C-5), 132.7 (C-3), 125.5 (C-2), 122.9 (C-1), 121.7 (C-4), 34.6 (C-9), 34.2 (C-8), 31.8 (C-10), 30.0 (C-11), 18.8 (C-7).

Data consistent with that reported in the literature.[4]

2,4-di-*tert*-butyl-1-nitrobenzene

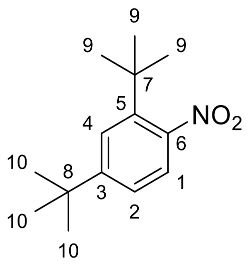

To a solution of 1,3-di-*tert*-butylbenzene (7.9 g, 41.8 mmol, 1.0 equiv.) in trifluoroacetic acid (32 mL) was added NH_4NO_3 (3.43 g, 42.8 mmol, 1.02 equiv.). The mixture was stirred for 2 h, poured on water and extracted with DCM. The organics were combined, washed with brine, dried over $MgSO_4$ and concentrated *in vacuo*. Recrystallization in isopropanol yielded the title compound as an off-white solid (6.4 g, 27.2 mmol, 65%).

δ^{1}H (400 MHz, $CDCl_3$) 7.55 (1 H, d, *J* = 1.5 Hz, H-4), 7.29 (1 H, dd, *J* = 8.4 Hz, 1.7, H-2), 7.26 (1 H, d, *J* = 8.2 Hz, H-1), 1.41 (9 H, s, H-9), 1.33 (9 H, s, H-10).

δ^{13}C (101 MHz, $CDCl_3$) 154.1 (C-3), 149.1 (C-6), 140.9 (C-5), 125.5 (C-4), 123.9 (C-2 or C-1), 123.8 (C-2 or C-1), 35.9 (C-7), 35.3 (C-8), 31.3 (C-10), 30.8 (C-9).

Data consistent with that reported in the literature.[5]

2,4-di-*tert*-butyl-aniline

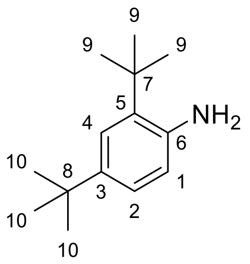

2,4-di-*tert*-butyl-1-nitrobenzene (4.1 g, 17.6 mmol, 1.0 equiv.) and palladium on activated charcoal (10 wt% Pd, 0.5 g) was suspended in a mixture of ethanol (35 mL) and hydrazine hydrate (25 mL). The mixture was refluxed for 16 h, concentrated under vacuum, taken up in a solution of 5 M NaOH and extracted with ethyl acetate. The organics were combined, washed with brine, dried over Na_2SO_4, and concentrated *in vacuo* to yield to the title compound as a yellow oil (3.4 g, 16.7 mmol, 95%)

δ^{1}H (400 MHz, $CDCl_3$) 7.28 (1 H, d, *J* = 2.3 Hz, H-4), 7.06 (1 H, dd, *J* = 8.2, 2.3 Hz, H-2), 6.61 (1 H, d, *J* = 8.2 Hz, H-1), 3.72 (2 H, br. s, NH), 1.43 (9 H, s, H-9), 1.28 (9 H, s, H-10).

δ^{13}C (101 MHz, $CDCl_3$) 142.1 (C-3), 141.2 (C-6), 133.3 (C-5), 123.8 (C-2), 123.7 (C-4), 117.6 (C-1), 34.7 (C-7), 34.3 (C-8), 31.7 (C-10), 29.8 (C-9).

IR (film, cm^{-1}) ν_{max} = 3502, 3407, 2956, 1621, 1484, 874;

HRMS ($APCI^+$) *m/z* calcd for $C_{15}H_{26}N$ $[M+H]^+$ 220.2065, found 220.6068.

2,4-di-*tert*-butyl-1-bromobenzene

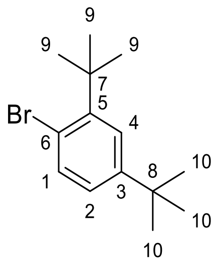

To a solution of 1,3-di-*tert*-butylbenzene (2.48 g, 13.0 mmol, 1.0 equiv.) in TMPO (26 mL) at 70°C was added dropwise a solution of bromine (0.84 mL, 16.4 mmol, 1.25 eq) in TMPO (14 mL). The mixture was stirred at this temperature for 20 h and extracted with petrol ether. The combined extraction layers were washed with a saturated aqueous solution of Na_2SO_3, brine, dried over $MgSO_4$ and concentrated *in vacuo* to yield to the title compound as a colourless oil (3.50 g, 13.0 mmol, quant.)

δ^1H (400 MHz, $CDCl_3$) 7.49 (1 H, d, *J* = 8.3 Hz, H-1), 7.46 (1 H, d, *J* = 2.4 Hz, H-4), 7.05 (1 H, dd, *J* = 8.3, 2.5 Hz, H-2), 1.51 (9 H, s, H-9), 1.30 (9 H, s, H-10).

δ^{13}C (101 MHz, $CDCl_3$) 150.1 (C-5), 146.9 (C-3), 135.3 (C-1), 125.3 (C-4), 124.7 (C-2), 119.5 (C-6), 36.9 (C-7), 34.9 (C-8), 31.4 (C-10), 29.8 (C-9).

Data consistent with that reported in the literature.[6]

2-isopropyl-*N*-(o-tolyl)aniline – 6a

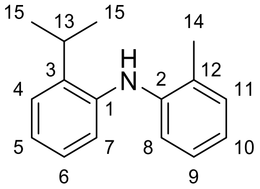

Following **GP1** with 2-bromotoluene (180 μL, 1.5 mmol, 1.0 equiv.), 2-isopropyl aniline (263 μL, 1.87 mmol, 1.25 eq), NaO*t*Bu (159 mg, 1.65 mmol, 1.5 equiv.), $Pd_2(dba)_3$ (13.7 mg, 0.015 mmol, 1 mol%), PNp_3 (7.3 mg, 0.03 mmol, 2 mol%) and toluene (3 mL). Filtration over a plug of silica (SiO_2, petroleum ether) yielded the title compound as a yellow oil (341 mg, 1.5 mmol, quant.).

δ^1H (400 MHz, $CDCl_3$) 7.33 (1 H, dd, *J* = 7.5, 1.5 Hz, H-4), 7.21 (1 H, d, *J* = 7.4 Hz, H-11), 7.14 (2 H, m, H-6 + H-9), 7.08 (1 H, m, H-7), 7.05 (1 H, m, H-5), 6.93 (1 H, d, *J* = 8.0 Hz, H-8), 6.88 (1 H, td, *J* = 7.4, 1.1 Hz, H-10), 5.25 (1 H, s, NH), 3.16 (1 H, sept, *J* = 5.8 Hz, H-13), 2.30 (3 H, s, H-14), 1.29 (6 H, d, *J* = 5.9 Hz, H-15).

δ^{13}C (101 MHz, $CDCl_3$) 143.2 (C-2), 140.4 (C-3), 139.5 (C-1), 130.8 (C-11), 127.0 (C-7), 126.6 (C-6), 126.5 (C-12), 126.1 (C-4), 122.8 (C-9), 121.0 (C-5), 120.7 (C-10), 117.0 (C-8), 27.9 (C-13), 23.1 (C-15), 18.0 (C-14).

IR (film, cm^{-1}) ν_{max} 3405 (N–H), 3033 (C–H), 2960 (C–H), 1584 (N–H), 1491;

HRMS (APCI$^+$) *m/z* calcd for $C_{16}H_{20}N$ $[M+H]^+$ 226.1596, found 226.1599.

2-(*tert*-butyl)-*N*-(2-isopropylphenyl)aniline – 6b

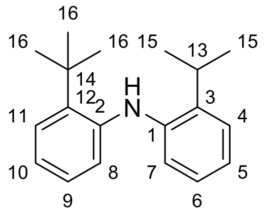

Following **GP1** with 1-bromo-2-*tert*-butylbenzene (180 µL, 1.5 mmol, 1.0 equiv.), 2-isopropylaniline (263µL, 1.87 mmol, 1.25 eq), NaO*t*Bu (159 mg, 1.65 mmol, 1.5 equiv.), $Pd_2(dba)_3$ (13.7 mg, 0.015 mmol, 1 mol%), PNp_3 (7.3 mg, 0.03 mmol, 2 mol%) in distilled toluene (3 mL). Filtration over a plug of silica (SiO_2, petroleum ether) yielded the title compound as an orange oil (388 mg, 1.4 mmol, 96%).

δ^{1}H (400 MHz, $CDCl_3$) 7.43 (1 H, dd, *J* = 7.9, 1.2 Hz, H-11), 7.32 (1 H, dd, *J* = 7.6, 1.0 Hz, H-4), 7.15 (1 H, m, H-9), 7.11 (1 H, m, H-6), 7.08 (1 H, d, *J* = 5.8 Hz, H-8), 7.01 (1 H, m, H-10), 6.99 (1 H, m, H-5), 6.96 (1 H, d, *J* = 7.9 Hz, H-7), 5.47 (1 H, br. s, NH), 3.12 (1 H, sept, *J* = 5.8 Hz, H-13), 1.50 (9 H, s, H-16), 1.33 (6 H, d, *J* = 5.8 Hz, H-15).

δ^{13}C (101 MHz, $CDCl_3$) 142.9 (C-2), 141.9 (C-1), 140.4 (C-12), 137.6 (C-3), 127.02 (C-11), 126.99 (C-9), 126.6 (C-6), 125.8 (C-4), 122.7 (C-8), 122.1 (C-10), 121.3 (C-5), 119.0 (C-7), 34.8 (C-14), 30.5 (C-16), 27.8 (C-13), 23.0 (C-15).

IR (film, cm^{-1}) ν_{max} 3479 (N–H), 2959 (C–H), 1580 (N–H), 1499, 1443;

HRMS (APCI$^+$) *m/z* calcd for $C_{19}H_{26}N$ $[M+H]^+$ 268.2065, found 268.2061.

N-(2-isopropylphenyl)-2,6-dimethylaniline – 6c

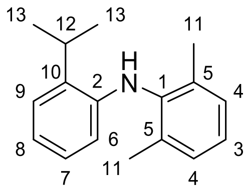

Following **GP1** with 2-bromo-1,3-dimethylbenzene (200 µL, 1.5 mmol, 1.0 equiv.), 2-isopropylaniline (263 µL, 1.87 mmol, 1.25 eq), NaO*t*Bu (159 mg, 1.65 mmol, 1.5 equiv.), $Pd_2(dba)_3$ (13.7 mg, 0.015 mmol, 1 mol%), PNp_3 (7.3 mg, 0.03 mmol, 2 mol%) in distilled toluene (3 mL). Filtration over a plug of silica (SiO_2, petroleum ether) yielded the title compound as an off-white solid (274 mg, 1.1 mmol, 76%),

m.p.: 116 – 119 °C (degradation).

δ^1H (400 MHz, $CDCl_3$) 7.25 (1 H, d, *J* = 7.2 Hz, H-9), 7.14 (2 H, d, *J* = 7.2 Hz, H-4), 7.09 (1 H, dd, *J* = 8.5, 6.2 Hz, H-3), 6.97 (1 H, t, *J* = 7.6 Hz, H-7), 6.81 (1 H, t, *J* = 7.2 Hz, H-8), 6.20 (1 H, d, *J* = 8.0 Hz, H-6), 5.14 (1 H, br. s, NH), 3.17 (1 H, sept, *J* = 5.8 Hz, H-12), 2.20 (6 H, s, H-11), 1.40 (6 H, d, *J* = 5.8 Hz, H-13)

δ^{13}C (101 MHz, $CDCl_3$) 142.9 (C-2), 139.0 (C-1), 135.5 (C-5), 133.0 (C-10), 128.7 (C-4), 126.6 (C-7), 125.43 (C-3), 125.34 (C-9), 118.7 (C-8), 112.7 (C-6), 27.8 (C-8), 22.5 (C-13), 18.5 (C-11).

IR (film, cm^{-1}) v_{max} 3407 (N–H), 3033 (C–H), 2956 (C–H), 1604 (N–H), 1581 (N–H), 1498, 1453;

HRMS (APCI$^+$) *m/z* calcd for $C_{17}H_{22}N$ $[M+H]^+$ 240.1752, found 240.1753

2-ethyl-*N*-(2-isopropylphenyl)-6-methylaniline – 6d

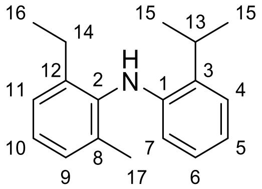

Following **GP1** with 1-bromo-2-isopropylbenzene (230 µL, 1.5 mmol, 1.0 equiv.), 2-ethyl-6-methylaniline (260 µL, 1.87 mmol, 1.25 eq), NaO*t*Bu (159 mg, 1.65 mmol, 1.5 equiv.), $Pd_2(dba)_3$ (13.7 mg, 0.015 mmol, 1 mol%), PNp_3 (7.3 mg, 0.03 mmol, 2 mol%) in distilled toluene (3 mL). Filtration over a plug of silica (SiO_2, petroleum ether) yielded the title compound as a yellow oil (348 mg, 1.4 mmol, 91%).

δ^1H (400 MHz, $CDCl_3$) 7.25 (1 H, d, *J* = 7.5 Hz, H-4), 7.18 (3 H, m, H-9 + H-10 + H-11), 6.97 (1 H, t, *J* = 7.7 Hz, H-6), 6.80 (1 H, t, *J* = 7.4 Hz, H-5), 6.18 (1 H, dd, *J* = 8.0, 0.9 Hz, H-1), 5.17 (1 H, s, NH), 3.15 (1 H, sept., *J* = 5.7 Hz, H-13), 2.58 (2 H, m, H-14), 2.18 (3 H, s, H-17), 1.40 (6 H, d, *J* = 5.8 Hz, H-15), 1.17 (3 H, t, *J* = 7.6 Hz, H-16).

δ^{13}C (101 MHz, $CDCl_3$) 143.3 (C-3), 141.5 (C-12), 138.2 (C-2), 136.0 (C-8), 132.6 (C-1), 128.7 (C-9), 126.9 (C-10), 126.6 (C-6), 125.8 (C-11), 125.3 (C-4), 118.5 (C-5), 112.5 (C-7), 27.7 (C-13), 24.9, (C-14) 22.5 (C-15), 22.5 (C-15), 18.6 (C-17), 14.9 (C-16).

IR (film, cm^{-1}) ν_{max} 3420 (N–H), 2961 (C–H), 2871 (C–H), 1604 (N–H), 1581 (N–H), 1498, 1454;

HRMS (APCI$^+$) *m/z* calcd for $C_{18}H_{24}N$ $[M+H]^+$ 254.1909, found 254.1910.

2-isopropyl-6-methyl-*N*-(o-tolyl)aniline – 6e

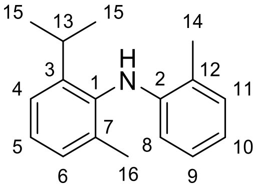

Following **GP1** with 2-bromotoluene (42 µL, 0.35 mmol, 1.0 equiv.), 2-isopropyl-6-methylaniline (68 µL, 0.44 mmol, 1.25 eq), NaO*t*Bu (50 mg, 0.52 mmol, 1.5 equiv.), $Pd_2(dba)_3$ (3.2 mg, 3.5 µmol, 1 mol%), PNp_3 (1.7 mg, 7 µmol, 2 mol%) in distilled toluene (0.7 mL). Filtration over a plug of silica (SiO_2, petroleum ether) yielded the title compound as a colourless oil (82 mg, 0.34 mmol, 98%).

δ^1H (400 MHz, $CDCl_3$) 7.34 (1 H, d, *J* = 7.2 Hz, H-4), 7.29 (1 H, t, *J* = 7.5 Hz, H-5), 7.23 (2 H, d, *J* = 7.0 Hz, H-6 + H-8), 7.06 (1 H, t, *J* = 7.6 Hz, H-10), 6.79 (1 H, t, *J* = 7.3 Hz, H-9), 6.23 (1 H, d, *J* = 8.0 Hz, H-11), 5.05 (1 H, br. s, NH), 3.26 (1 H, sept., *J* = 5.8 Hz, H-13), 2.44 (3 H, s, H-14), 2.26 (3 H, s, H-16), 1.27 (6 H, br. s, H-15).

δ^{13}C (101 MHz, $CDCl_3$) 146.7 (C-3), 145.1 (C-7), 137.3 (C-1), 136.5 (C-2), 130.3 (C-8), 128.4 (C-6), 127.1 (C-10), 126.5 (C-5), 124.0 (C-4), 122.0 (C-12), 117.8 (C-9), 111.5 (C-11), 28.2 (C-13), 24.6 (C-15), 23.2 (C-15), 18.6 (C-16), 17.8 (C-14).

IR (film, cm^{-1}) ν_{max} 3412 (N–H), 3034 (C–H), 2961 (C–H), 2867 (C–H), 1606 (N–H), 1584 (N–H), 1500, 1462;

HRMS (APCI$^+$) *m/z* calcd for $C_{17}H_{22}N$ $[M+H]^+$ 240.1752, found 240.1753.

2-isopropyl-*N*-(2-isopropylphenyl)-6-methylaniline – 6f

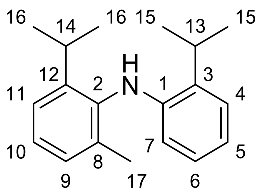

Following **GP1** with 2-bromotoluene (230 μL, 1.5 mmol, 1.0 equiv.), 2-isopropyl-6-methylaniline (0.29 mL, 1.87 mmol, 1.25 eq), NaO*t*Bu (159 mg, 1.65 mmol, 1.5 equiv.), $Pd_2(dba)_3$ (13.7 mg, 0.015 mmol, 1 mol%), PNp_3 (7.3 mg, 0.03 mmol, 2 mol%) in distilled toluene (3 mL). Filtration over a plug of silica (SiO_2, petroleum ether) yielded the title compound as a yellow oil (334 mg, 1.2 mmol, 83%).

δ^{1}H (400 MHz, $CDCl_3$) 7.26 (2 H, d, *J* = 7.5 Hz, H-4 + H-11), 7.21 (1 H, t, *J* = 7.5 Hz, H-10), 7.16 (1 H, d, *J* = 7.2 Hz, H-9), 6.97 (1 H, t, *J* = 7.7 Hz, H-6), 6.80 (1 H, t, *J* = 7.4 Hz, H-5), 6.18 (1 H, dd, *J* = 8.0, 0.8 Hz, H-7), 5.17 (1 H, s, NH), 3.16 (1 H, m, H-14), 3.15 (1 H, m, H-13), 2.19 (3 H, s, H-17), 1.41 (6 H, d, *J* = 5.8 Hz, H-15), 1.20 ($C\mathit{H_A}H_B$, 3 H, d, *J* = 5.8 Hz, H-16), 1.18 ($CH_A\mathit{H_B}$, 3 H, d, *J* = 5.9 Hz, H-16).

δ^{13}C (101 MHz, $CDCl_3$) 146.5 (C-12), 143.7 (C-2), 137.4 (C-3), 136.3 (C-8), 132.3 (C-1), 128.5 (C-5), 126.7 (C-6), 126.3 (C-10), 125.2 (C-4), 124.0 (C-11), 118.3 (C-5), 112.2 (C-7), 28.2 (C-14), 27.8 (C-13), 24.6 (C-16), 23.1 (C-16), 22.48 (C-15), 22.43 (C-15), 18.8 (C-17).

IR (film, cm^{-1}) v_{max} 3423 (N–H), 3035 (C–H), 2960 (C–H), 2868 (C–H), 1604 (N–H), 1581 (N–H), 1497, 1454;

HRMS (APCI$^+$) *m/z* calcd for $C_{19}H_{26}N$ $[M+H]^+$ 268.2065 found 268.2056.

2,6-diisopropyl-*N*-(o-tolyl)aniline – 6g

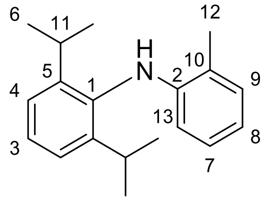

Following **GP1** with 2-bromotoluene (230 μL, 1.5 mmol, 1.0 equiv.), 2,6-diisopropyl aniline (260 μL, 1.87 mmol, 1.25 eq), NaO*t*Bu (159 mg, 1.65 mmol, 1.5 equiv.), $Pd_2(dba)_3$ (13.7 mg, 0.015 mmol, 1 mol%), PNp_3 (7.3 mg, 0.03 mmol, 2 mol%) in distilled toluene (3 mL). Filtration over a plug of silica (SiO_2, petroleum ether) yielded the title compound as a colourless oil (348 mg, 1.3 mmol, 87%).

δ^1H (500 MHz, $CDCl_3$) 7.31 (1 H, m, H-3), 7.25 (2 H, m, H-4), 7.16 (1 H, d, *J* = 7.3 Hz, H-13), 6.97 (1 H, t, *J* = 7.7 Hz, H-7), 6.69 (1 H, t, *J* = 7.3 Hz, H-8), 6.14 (1 H, d, *J* = 8.0 Hz, H-9), 4.93 (1 H, br. s, NH), 3.13 (2 H, sept, *J* = 5.8 Hz, H-11), 2.37 (3 H, s, H-12), 1.18 (CH_AH_B, 6 H, t, *J* = 7.5 Hz, H-6), 1.14 (CH_AH_B, 6 H, d, *J* = 5., H-6).

δ^{13}C (126 MHz, $CDCl_3$) 147.4 (C-1), 146.2 (C-2), 135.9 (C-5), 130.2 (C-9), 127.2 (C-3), 127.1 (C-7), 123.9 (C-4), 121.4 (C-10), 117.6 (C-8), 111.6 (C-13), 28.4 (C-11), 24.9 (C-6), 23.1 (C-6), 17.8 (C-12).

IR (film, cm^{-1}) ν_{max} 3425 (N–H), 2961 (C–H), 1606 (N–H), 1585 (N–H), 1500;

HRMS (APCI$^+$) *m/z* calcd for $C_{19}H_{26}N$ $[M+H]^+$ 268.2065, found 268.2066.

2,6-diisopropyl-*N*-(2-isopropylphenyl)aniline – 6h

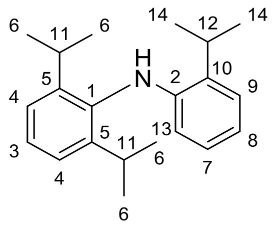

Following **GP1** with 1-bromo-2-isopropylbenzene (54 μL, 0.35 mmol, 1.0 equiv.), 2,6-diisopropylaniline (83 μL, 0.44 mmol, 1.25 eq), NaO*t*Bu (50 mg, 0.52 mmol, 1.5 equiv.), $Pd_2(dba)_3$ (3.2 mg, 3.5 μmol, 1 mol%), PNp_3 (1.7 mg, 7 μmol, 2 mol%) in distilled toluene (0.7 mL). Filtration over a plug of silica (SiO_2, petroleum ether) yielded the title compound as a yellow oil (90 mg, 0.23 mmol, 65%).

δ^{1}H (400 MHz, $CDCl_3$) 7.31 (1 H, dd, *J* = 8.6, 6.4 Hz, H-3), 7.26 (2 H, m, H-4), 7.24 (1 H, br. d, H-6), 6.95 (1 H, t, *J* = 7.3 Hz, H-8), 6.77 (1 H, t, *J* = 7.4 Hz, H-7), 6.16 (1 H, d, *J* = 8.0 Hz, H-9), 5.11 (1 H, s, NH), 3.12 (3 H, m, H-12 + H-11), 1.41 (6 H, d, *J* = 5.8 Hz, H-14), 1.18 (CH_AH_B, 6 H, d, *J* = 5.9 Hz, H-13), 1.13 (CH_AH_B, 6 H, d, *J* = 5.9 Hz, H-13).

δ^{13}C (101 MHz, $CDCl_3$) 147.2 (C-5), 144.7 (C-2), 135.8 (C-1), 131.6 (C-10), 127.0 (C-3), 126.6 (C-8), 125.1 (C-6), 124.0 (C-4), 117.9 (C-7), 112.1 (C-9), 28.3 (C-11), 27.8 (C-12), 24.9 (C-13), 23.0 (C-13), 22.4 (C-14).

IR (film, cm^{-1}) ν_{max} 3432 (N–H), 3034 (C–H), 2961 (C–H), 1605 (N–H), 1581 (N–H), 1498, 1445;

HRMS (APCI$^+$) *m/z* calcd for $C_{21}H_{30}N$ $[M+H]^+$ 296.2378, found 296.2364.

***N*-(2-(*tert*-butyl)phenyl)-2-ethyl-6-methylaniline – 6i**

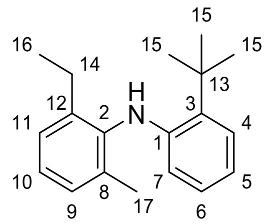

Following **GP1** with 1-bromo-2-*tert*-butylbenzene (476 mg, 2.2 mmol, 1.0 equiv.), 2-ethyl-6-methylaniline (0.39 mL, 2.8 mmol, 1.25 eq), NaO*t*Bu (0.24 g, 2.5 mmol, 1.1 equiv.), $Pd_2(dba)_3$ (20.6 mg, 0.022 mmol, 1 mol%), PNp_3 (11.0 mg, 0.045 mmol, 2 mol%) in distilled toluene (4.5 mL). Filtration over a plug of silica (SiO_2, petroleum ether) yielded the title compound as an off-white solid (425 mg, 1.6 mmol, 72%),

m.p.: 67 – 68 °C.

δ^{1}H (400 MHz, $CDCl_3$) 7.34 (1 H, dd, *J* = 7.8, 1.2 Hz, H-4), 7.17 (1 H, dd, *J* = 8.9, 4.7 Hz, H-10), 7.14 (2 H, m, H-9 + H-11), 6.96 (1 H, t, *J* = 7.6 Hz, H-6), 6.75 (1 H, t, *J* = 7.5 Hz, H-5), 6.20 (1 H, dd, *J* = 8.0, 0.8 Hz, H-7), 5.36 (1 H, s, br.s, NH), 2.60 (CH_AH_B, 1 H, sept., *J* = 7.6 Hz, H-14), 2.57 (CH_AH_B, 1 H, sept., *J* = 7.6 Hz, H-14), 2.16 (3 H, s, H-17), 1.56 (9 H, s, H-15), 1.16 (3 H, t, *J* = 7.6 Hz, H-16).

δ^{13}C (101 MHz, $CDCl_3$) 144.5 (C-3), 141.3 (C-12), 138.4 (C-2), 135.9 (C-6), 133.9 (C-1), 128.8 (C-9), 127.1 (C-6), 126.9 (C-10), 126.5 (C-4), 125.7 (C-11), 118.3 (C-5), 113.8 (C-7), 34.6 (C-13), 30.0 (C-15), 25.0 (C-14), 18.8 (C-17), 15.0 (C-16).

IR (film, cm^{-1}) ν_{max} 3458 (N–H), 2963, (C–H), 1600 (N–H), 1591 (N–H), 1576;

HRMS (APCI$^+$) *m/z* calcd for $C_{19}H_{26}N$ $[M+H]^+$ 268.2065, found 268.2053.

_N_-(2-(_tert_-butyl)phenyl)-2-isopropyl-6-methylaniline – 6j

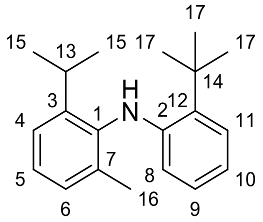

Following **GP1** with 1-bromo-2-*tert*-butylbenzene (73 mg, 0.35 mmol, 1.0 equiv.), 2-isopropyl-6-methylaniline (68 μL, 0.44 mmol, 1.25 eq), NaO*t*Bu (50 mg, 0.52 mmol, 1.5 equiv.), $Pd_2(dba)_3$ (3.2 mg, 3.5 μmol, 1 mol%), PNp_3 (1.7 mg, 7 μmol, 2 mol%) in distilled toluene (0.7 mL). The reaction time was 48 h, after which filtration over a plug of silica (SiO_2, petroleum ether) yielded the title compound as a yellow oil (34 mg, 0.12 mmol, 34%).

δ^1H (400 MHz, $CDCl_3$) 7.38 (1 H, dd, *J* = 7.8, 1.0, H-8), 7.28 (1 H, d, *J* = 7.4 Hz, H-4), 7.22 (1 H, t, *J* = 7.5 Hz, H-5), 7.18 (1 H, d, *J* = 7.0 Hz, H-6), 6.99 (1 H, t, *J* = 7.2 Hz, H-9), 6.78 (1 H, t, *J* = 7.4 Hz, H-10), 6.24 (1 H, d, *J* = 8.0 Hz, H-11), 5.39 (1 H, s, NH), 3.15 (1 H, sept, *J* = 5.8 Hz, H-13), 2.20 (3 H, s, H-16), 1.60 (9 H, s, H-17), 1.23 (3 H, d, *J* = 5.9 Hz, H-15), 1.19 (3 H, d, *J* = 5.9 Hz, H-15).

δ^{13}C (101 MHz, $CDCl_3$) 146.3 (C-1), 144.9 (C-2), 137.6 (C-3), 136.1 (C-7), 133.5 (C-12), 128.6 (C-6), 127.1 (C-2), 126.5 (C-8), 126.1 (C-5), 124.0 (C-4), 118.1 (C-3), 113.6 (C-11), 34.5 (C-14), 30.0 (C-17), 28.3 (C-13), 24.7 (C-15), 22.9 (C-15), 19.0 (C-16).

IR (film, cm^{-1}) v_{max} 3463 (N–H), 3049 (C–H), 2963 (C–H), 2871 (C–H), 1600 (N–H), 1576 (N–H), 1497, 1443;

HRMS (APCI$^+$) *m/z* calcd for $C_{20}H_{28}N$ $[M+H]^+$ 282.2222, found 282.2236.

***N*-(2-(*tert*-butyl)phenyl)-2,6-diisopropylaniline – 6k**

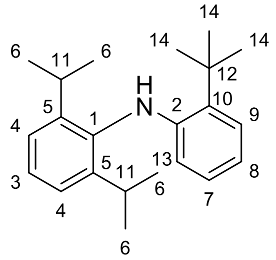

Following **GP1.1** with 1-bromo-2-*tert*-butylbenzene (75 mg, 0.35 mmol, 1.0 equiv.), 2,6-diisopropylaniline (83 μL, 0.44 mmol, 1.25 eq), NaO*t*Bu (50 mg, 0.52 mmol, 1.5 equiv.), $Pd_2(dba)_3$ (3.2 mg, 3.5 μmol, 1 mol%), PNp_3 (1.7 mg, 7 μmol, 2 mol%) in distilled toluene (0.7 mL). Filtration over a plug of silica (SiO_2, petroleum ether) yielded the title compound as a yellow oil (80 mg, 0.26 mmol, 76%).

δ^1H (400 MHz, $CDCl_3$) 7.34 (1 H, dd, *J* = 7.9, 1.0 Hz, H-9), 7.30 (1 H, m, H-3), 7.25 (2 H, m, H-4), 6.95 (1 H, t, *J* = 7.6 Hz, H-7), 6.73 (1 H, t, *J* = 7.5 Hz, H-8), 6.19 (1 H, d, *J* = 8.0 Hz, H-6), 5.28 (1 H, s, NH), 3.09 (2 H, sept, *J* = 5.8 Hz, H-11), 1.57 (9 H, s, H-14), 1.18 (CH_AH_B, 6 H, d, *J* = 5.9 Hz, H-13), 1.12 (CH_AH_B, 6 H, d, *J* = 5.9 Hz, H-13).

δ^{13}C (101 MHz, $CDCl_3$) 147.1 (C-5), 146.0 (C-2), 136.0 (C-1), 132.9 (C-10), 127.1 (C-7), 126.9 (C-3), 126.4 (C-9), 124.1 (C-4), 117.8 (C-8), 113.6 (C-6), 34.4 (C-12), 29.9 (C-14), 28.4 (C-11), 25.0 (C-13), 22.9 (C-13).

IR (film, cm^{-1}) ν_{max} 3468 (N–H), 2961 (C–H), 2869 (C–H), 1601 (N–H), 1576 (N–H), 1497, 1443;

HRMS (APCI$^+$) *m/z* calcd for $C_{22}H_{32}N$ $[M+H]^+$ 310.2535, found 310.2520.

***N*-(2,4-di-*tert*-butylphenyl)-2-methylnaphthale*N*-1-amine – 6l**

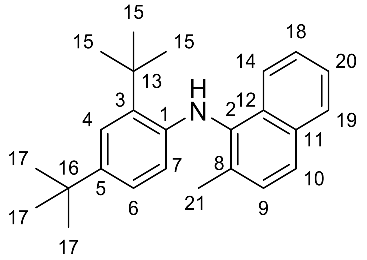

Following **GP1** with 1-bromo-2-methylnaphthalene (63 µL, 0.41 mmol, 1.0 equiv.), 2,4-di-*tert*-butyl-aniline (104 mg, 0.51 mmol, 1.25 eq), NaO*t*Bu (58 mg, 0.61 mmol, 1.5 equiv.), $Pd_2(dba)_3$ (3.7 mg, 4.1 µmol, 1 mol%), PNp_3 (2.0 mg, 8.2 µmol, 2 mol%) in distilled toluene (0.8 mL). Filtration over a plug of silica (SiO_2, petroleum ether) yielded the title compound as a yellow oil (93 mg, 0.27 mmol, 66%).

δ^1H (400 MHz, $CDCl_3$) 7.91 (1 H, d, *J* = 8.1 Hz, H-14), 7.85 (1 H, d, *J* = 7.5 Hz, H-19), 7.68 (1 H, d, *J* = 8.4 Hz, H-9), 7.41 (4 H, m, H-4 + H-10 + H-18 + H-20), 6.88 (1 H, dd, *J* = 8.4, 2.3 Hz, H-6), 6.09 (1 H, d, *J* = 8.4 Hz, H-7), 5.63 (1 H, br. s, NH), 2.38 (3 H, s, H-21), 1.68 (9 H, s, H-15), 1.29 (9 H, s, H-17).

δ^{13}C (101 MHz, $CDCl_3$) 142.2 (C-5), 140.9 (C-3), 135.9 (C-2), 133.6 (C_{Ar}), 133.5 (C_{Ar}), 131.4 (C_{Ar}), 131.2 (C_{Ar}), 129.5 (C-4), 128.3 (C-19), 126.2 (C-18 or C-20), 125.24 (C-18 or C-20), 125.21 (C-9), 123.7 (C-14), 123.6 (C-6 + C-10), 114.8 (C-7), 35.0 (C-13), 34.3 (C-16), 31.7 (C-17), 30.3 (C-15), 19.0 (C-21).

IR (film, cm^{-1}) v_{max} 3450 (N–H), 3052 (C–H), 2959 (C–H), 2868 (C–H), 1608 (N–H), 1571 (N–H), 1491, 1391;

HRMS (APCI$^+$) *m/z* calcd for $C_{25}H_{32}N$ $[M+H]^+$ 346.2535, found 346.2541.

2,4-di-*tert*-butyl-6-methyl-*N*-(o-tolyl)aniline – 6m

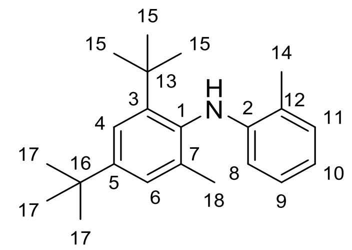

Following **GP1** with 2-bromotoluene (49 µL, 0.41 mmol, 1.0 equiv.), 2,4-di-*tert*-butyl-6-methylaniline (111 mg, 0.51 mmol, 1.25 eq), NaO*t*Bu (58 mg, 0.61 mmol, 1.5 equiv.), $Pd_2(dba)_3$ (3.7 mg, 4.1 µmol, 1 mol%), PNp_3 (2.0 mg, 8.2 µmol, 2 mol%) in distilled toluene (0.8 mL). Filtration over a plug of silica (SiO_2, petroleum ether) yielded the title compound as a colourless oil (123 mg, 0.40 mmol, 97%).

δ^1H (400 MHz, $CDCl_3$) 7.36 (1 H, d, *J* = 2.1 Hz, H-4), 7.16 (1 H, d, *J* = 2.0 Hz, H-6), 7.13 (1 H, d, *J* = 7.3 Hz, H-11), 6.97 (1 H, t, *J* = 7.6 Hz, H-9), 6.66 (1 H, t, *J* = 7.1 Hz, H-10), 6.09 (1 H, d, *J* = 8.0 Hz, H-8), 5.06 (1 H, br. s, NH), 2.31 (3 H, s, H-14), 2.06 (3 H, s, H-18), 1.38 (9 H, s, H-15), 1.35 (9 H, s, H-17).

δ^{13}C (101 MHz, $CDCl_3$) 148.4 (C-5), 147.0 (C-3), 145.2 (C-2), 137.6 (C-1), 135.9 (C-7), 130.2 (C-11), 127.0 (C-9), 126.3 (C-6), 122.0 (C-4), 121.4 (C-12), 117.3 (C-6), 112.1 (C-8), 35.7 (C-13), 34.7 (C-16), 31.6 (C-17), 31.4 (C-15), 19.3 (C-18), 17.9 (C-14).

IR (film, cm^{-1}) v_{max} 3457 (N–H), 2961 (C–H), 2867 (C–H), 1607 (N–H), 1586 (N–H), 1492, 1471;

HRMS ($APCI^+$) *m/z* calcd for $C_{22}H_{32}N$ $[M+H]^+$ 310.2535, found 310.2531.

2,4-di-*tert*-butyl-*N*-(2-isopropylphenyl)-6-methylaniline – 6n

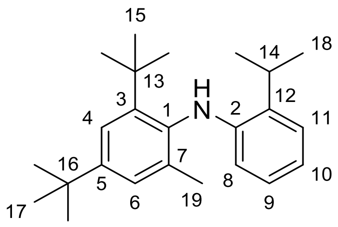

Following **GP1** with 1-bromo-2-isopropylbenzene (62 µL, 0.41 mmol, 1.0 equiv.), 2,4-di-*tert*-butyl-6-methylaniline (111 mg, 0.51 mmol, 1.25 eq), NaO*t*Bu (58 mg, 0.61 mmol, 1.5 equiv.), $Pd_2(dba)_3$ (3.7 mg, 4.1 µmol, 1 mol%), PNp_3 (2.0 mg, 8.2 µmol, 2 mol%) in distilled toluene (0.8 mL). Filtration over a plug of silica (SiO_2, petroleum ether) yielded the title compound as a colourless oil (20 mg, 0.06 mmol, 14%).

δ^1H (400 MHz, $CDCl_3$) 7.37 (1 H, d, *J* = 2.0 Hz, H-4), 7.23 (1 H, d, *J* = 7.6 Hz, H-11), 7.16 (1 H, d, *J* = 1.8 Hz, H-6), 6.95 (1 H, t, *J* = 7.6 Hz, H-9), 6.75 (1 H, t, *J* = 7.4 Hz, H-10), 6.12 (1 H, d, *J* = 8.0 Hz, H-8), 5.25 (1 H, s, NH), 3.07 (1 H, sept., *J* = 5.7 Hz, H-14), 2.06 (3 H, s, H-19),1.39 (9 H, s, H-17), 1.37 (6 H, m, C-18), 1.35 (9 H, s, H-15).

δ^{13}C (101 MHz, $CDCl_3$) 148.2 (C-3), 146.9 (C-5), 143.9 (C-12), 137.4 (C-1), 136.1 (C-7), 132.0 (C-12), 126.5 (C-9), 126.5 (C-4), 125.1 (C-8), 122.0 (C-6), 117.8 (C-10), 113.1 (C-8), 35.7 (C-13), 34.7 (C-16), 31.7 (C-17), 31.4 (C-15), 27.7 (C-14), 22.8 (C-18), 22.4 (C-18), 19.5 (C-19).

IR (film, cm^{-1}) ν_{max} 2961 (C–H), 2869 (C–H), 1605 (N–H), 1582 (N–H), 1498, 1456, 1362;

HRMS (APCI$^+$) *m/z* calcd for $C_{24}H_{36}N$ $[M+H]^+$ 338.2848, found 338.2833.

***N*-(2,4-di-*tert*-butyl-6-methylphenyl)-[1,1'-biphenyl]-2-amine – 6o**

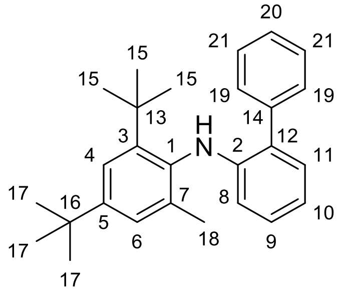

Following **GP1** with 2-bromo-1,1'-biphenyl (70 µL, 0.41 mmol, 1.0 equiv.), 2,4-di-*tert*-butyl-6-methylaniline (111 mg, 0.51 mmol, 1.25 eq), NaO*t*Bu (58 mg, 0.61 mmol, 1.5 equiv.), $Pd_2(dba)_3$ (3.7 mg, 4.1 µmol, 1 mol%), PNp_3 (2.0 mg, 8.2 µmol, 2 mol%) in distilled toluene (0.8 mL). Filtration over a plug of silica (SiO_2, petroleum ether) yielded the title compound as a colourless oil (9 mg, 0.024 mmol, 6%).

δ^1H (400 MHz, $CDCl_3$) 7.52 (2 H, d, *J* = 7.1 Hz, H-19), 7.46 (2 H, t, *J* = 7.6 Hz, H-21), 7.36 (1 H, t, *J* = 7.3 Hz, H-20), 7.30 (1 H, d, *J* = 1.9 Hz, H-4), 7.17 (1 H, d, *J* = 7.4 , H-11), 7.13 (1 H, d, *J* = 1.8 Hz, H-6), 7.09 (1 H, t, *J* = 7.7 Hz, H-9), 6.76 (1 H, t, *J* = 7.3 Hz, H-10), 6.18 (1 H, d, *J* = 8.1 Hz, H-8), 5.32 (1 H, br. s, NH), 2.09 (3 H, s, H-18), 1.32 (9 H, s, H-17), 1.28 (9 H, s, H-15).

δ^{13}C (101 MHz, $CDCl_3$) 148.6 (C-5), 147.5 (C-3), 144.3 (C-2), 139.6 (C-14), 137.7 (C-1), 135.6 (C-7), 130.0 (C-11), 129.7 (C-19), 129.0 (C-21), 128.6 (C-9), 127.5 (C-20), 127.4 (C-12), 126.4 (C-6), 122.0 (C-4), 117.1 (C-10), 112.3 (C-8), 35.7 (C-13), 34.7 (C-16), 31.6 (C-17), 31.2 (C-15), 19.6 (C-18).

IR (film, cm^{-1}) ν_{max} 3480 (N–H), 2959 (C–H), 2867 (C–H), 1592 (N–H), 1466;

HRMS (APCI$^+$) *m/z* calcd for $C_{27}H_{34}N$ $[M+H]^+$ 372.2691, found 372.2692.

2,4-di-*tert*-butyl-*N*-(2,4-di-*tert*-butylphenyl)-6-methylaniline – 6p

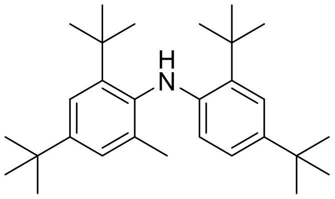

Following **GP1.1** with 2,4-di-*tert*-butyl-6-methylaniline (109 mg, 0.41 mmol, 1.0 equiv.), 2,4-di-*tert*-butyl-1-bromobenzene (111 mg, 0.51 mmol, 1.25 eq), NaO*t*Bu (58 mg, 0.61 mmol, 1.5 equiv.), $Pd_2(dba)_3$ (3.7 mg, 4.1 µmol, 1 mol%), PNp_3 (2.0 mg, 8.2 µmol, 2 mol%) in distilled toluene (0.8 mL). Filtration over a plug of silica (SiO_2, petroleum ether) yielded the title compound as a colourless oil (3 mg, 0.0074 mmol, 2%).

δ^1H (400 MHz, $CDCl_3$) 7.35 (1 H, d, *J* = 2.1 Hz, $C_{Ar}H$), 7.30 (1 H, d, *J* = 2.2 Hz, $C_{Ar}H$), 7.12 (1 H, d, *J* = 1.8 Hz, $C_{Ar}H$), 6.91 (1 H, dd, *J* = 8.4, 2.1 Hz, $C_{Ar}H$), 6.08 (1 H, d, *J* = 8.4 Hz, $C_{Ar}H$), 5.30 (1 H, br. s, NH), 2.00 (3 H, s, CH_3), 1.54 (9 H, s, $C(CH_3)_3$), 1.39 (9 H, s, $C(CH_3)_3$), 1.33 (9 H, s, $C(CH_3)_3$), 1.26 (9 H, s, $C(CH_3)_3$).

IR (film, cm^{-1}) v_{max} 2958 (C–H), 2855 (C–H), 1610 (N–H), 1500, 1480, 1393;

HRMS (APCI$^+$) *m/z* calcd for $C_{29}H_6N$ $[M+H]^+$ 408.3630, found 408.3646.

Too little material was isolated for the measurement of a ^{13}C NMR spectrum.

***N*-(2-ethyl-6-methylphenyl)-2-methylnaphthale*N*-1-amine – 6q**

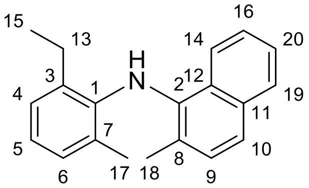

Following **GP1** with 1-bromo-2-methylnaphthalene (0.31 mL, 2.0 mmol, 1.0 equiv.), 2-ethyl-6-methylaniline (0.36 mL, 2.5 mmol, 1.25 eq), NaO*t*Bu (0.24 g, 2.5 mmol, 1.25 equiv.), $Pd_2(dba)_3$ (9.1 mg, 0.01 mmol, 0.5 mol%), PNp_3 (4.9 mg, 0.02 mmol, 1 mol%) in distilled toluene (4 mL). The reaction time was 48 h, after which filtration over a plug of silica (SiO_2, petroleum ether) yielded the title compound as a green oil (548 mg, 2.0 mmol, quant.).

δ^1H (400 MHz, $CDCl_3$) 7.95 (1 H, d, *J* = 8.2 Hz, H-14), 7.80 (1 H, d, *J* = 7.7 Hz, H-19), 7.48 (1 H, d, *J* = 8.3 Hz, H-10), 7.39 (2 H, m, H-16 + H-20), 7.24 (1 H, d, *J* = 8.3 Hz, H-9), 7.10 (1 H, d, *J* = 7.4 Hz, H-6), 6.98 (1 H, d, *J* = 7.4 Hz, H-4), 6.93 (1 H, t, *J* = 7.4 Hz, H-5), 2.54 (2 H, q, *J* = 7.5 Hz, H-13), 2.07 (3 H, s, H-18), 1.86 (3 H, s, H-17), 1.17 (3 H, t, *J* = 7.5 Hz, H-15).

δ^{13}C (101 MHz, $CDCl_3$) 141.9 (C-13), 137.7 (C-2), 134.5 (C-1), 133.4 (C-11), 129.9 (C-9), 129.3 (C-7), 129.2 (C-4), 128.9 (C-12), 128.3 (C-19), 126.7 (C-6), 125.7 (C-8), 125.6 (C-16 or C-20), 125.1 (C-16 or C-20), 122.6 (C-14), 122.4 (C-10), 121.9 (C-5), 25.1 (C-13), 19.44 (C-17 or C-18), 19.39 (C-17 or C-18), 14.0 (C-15).

IR (film, cm^{-1}) ν_{max} 3416 (N–H), 3049 (C–H), 2965 (C–H), 1593 (N–H), 1569, 1468.

HRMS (APCI$^+$) *m/z* calcd for $C_{20}H_{22}N$ $[M+H]^+$ 276.1747, found 276.1767.

2,4-di-*tert*-butyl-*N*-(2-isopropyl-6-methylphenyl)-6-methylaniline – 6r

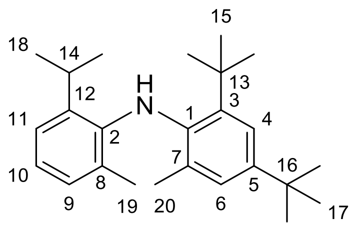

Following **GP1** with 2-bromo-1,5-di-*tert*-butyl-3-methylbenzene (115 mg, 0.41 mmol, 1.0 equiv.), 2-isopropyl-6-methylaniline (79 µL, 0.51 mmol, 1.25 eq), NaO*t*Bu (58 mg, 0.61 mmol, 1.5 equiv.), $Pd_2(dba)_3$ (3.7 mg, 4.1 µmol, 1 mol%), PNp_3 (2.0 mg, 8.2 µmol, 2 mol%) in distilled toluene (0.8 mL). Filtration over a plug of silica (SiO_2, petroleum ether) yielded the title compound as a colourless oil (11 mg, 0.03 mmol, 7%).

δ^{1}H (400 MHz, $CDCl_3$) 7.28 (1 H, d, *J* = 2.1 Hz, H-4), 7.11 (1 H, d, *J* = 7.5 Hz, H-11), 6.95 (1 H, d, *J* = 1.9 Hz, H-6), 6.92 (1 H, d, *J* = 5.7 Hz, H-9), 6.85 (1 H, t, *J* = 7.5 Hz, H-10), 5.34 (1 H, br. s, NH), 3.02 (1 H, dt, *J* = 13.6, 6.8 Hz, H-14), 1.86 (3 H, s, H-19), 1.72 (3 H, s, H-20), 1.51 (9 H, s, H-15), 1.31 (9 H, s, H-17), 1.18 ($C\mathit{H}_A H_B$, 3 H, d, *J* = 5.8 Hz, H-18), 1.14 ($CH_A \mathit{H}_B$, 3 H, d, *J* = 5.8, H-18).

δ^{13}C (101 MHz, $CDCl_3$) 144.8 (C-5), 140.7 (C-2), 139.8 (C-3), 139.3 (C-1), 137.6 (C-12), 131.5 (C-7), 129.1 (C-9), 127.5 (C-8), 126.5 (C-6), 124.1 (C-11), 121.7 (C-4), 120.6 (C-10), 35.6 (C-13), 34.5 (C-16), 31.7 (C-17), 31.0 (C-15), 27.8 (C-14), 24.7 (C-18), 22.3 (C-18), 20.4 (C-20), 19.9 (C-19).

IR (film, cm^{-1}) ν_{max} 3439 (N–H), 2955 (C–H), 2925 (C–H), 2867 (C–H), 1605 (N–H), 1580 (N–H), 1503, 1486, 1458;

HRMS (APCI$^+$) *m/z* calcd for $C_{25}H_{38}N$ $[M+H]^+$ 352.3004, found 352.3021.

_N_-(2-isopropyl-6-methylphenyl)-2-methylnaphthale_N_-1-amine – 6s

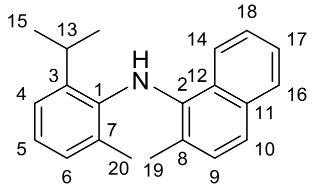

Following **GP1** with 1-bromo-2-methylnaphthalene (55 µL, 0.35 mmol, 1.0 equiv.), 2-isopropyl-6-methylaniline (68 µL, 0.44 mmol, 1.25 eq), NaO*t*Bu (50 mg, 0.52 mmol, 1.5 equiv.), $Pd_2(dba)_3$ (3.2 mg, 3.5 µmol, 1 mol%), PNp_3 (1.7 mg, 7 µmol, 2 mol%) in distilled toluene (0.7 mL). Filtration over a plug of silica (SiO_2, petroleum ether) yielded the title compound as a pink oil (86 mg, 0.30 mmol, 85%).

δ^1H (400 MHz, $CDCl_3$) 7.94 (1 H, d, *J* = 8.3 Hz, H-14), 7.82 (1 H, d, *J* = 8.2 Hz, H-16), 7.47 (1 H, d, *J* = 8.3 Hz, H-10), 7.42 (1 H, t, *J* = 7.9 Hz, H-17), 7.37 (1 H, m, H-18), 7.25 (1 H, d, *J* = 8.2 Hz, H-9), 7.21 (1 H, d, *J* = 7.4 Hz, H-4), 7.02 (1 H, , t, *J* = 7.4 Hz, H-5), 6.99 (1 H, d, *J* = 7.3 Hz, H-6), 5.49 (1 H, s, NH), 3.27 (1 H, sept, *J* = 5.8 Hz, H-13), 2.10 (3 H, s, H-19), 1.85 (3 H, s, H-20), 1.24 (CH_AH_B, 3 H, d, *J* = 5.8 Hz, H-15), 1.20 (CH_AH_B, 3 H, d, *J* = 5.8 Hz, H-15).

δ^{13}C (101 MHz, $CDCl_3$) 141.0 (C-7), 140.0 (C-3), 138.1 (C-2), 133.5 (C-12), 130.5 (C-1), 130.1 (C-9), 128.9 (C-6), 128.4 (C-16), 128.3 (C-12), 125.4 (C-18), 125.1 (C-17), 124.6 (C-8), 123.9 (C-4), 122.7 (C-5), 122.5 (C-14), 121.9 (C-10), 28.1 (C-13), 23.8 (C-15), 23.0 (C-15), 19.6 (C-20), 19.5 (C-19).

IR (film, cm^{-1}) v_{max} 3423 (N–H), 3051 (C–H), 2960 (C–H), 2866 (C–H), 1593 (N–H), 1568, 1465;

HRMS (APCI$^+$) *m/z* calcd for $C_{21}H_{24}N$ $[M+H]^+$ 290.1909, found 290.1895.

***N*-(2-(*tert*-butyl)-6-methylphenyl)-2-methylnaphthale*N*-1-amine – 6t**

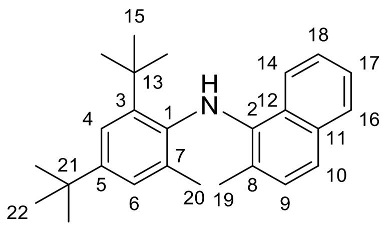

Following **GP1** with 1-bromo-2-methylnaphthalene (55 µL, 0.35 mmol, 1.0 equiv.), 2,4-di-*tert*-butyl-6-methylaniline (96 mg, 0.44 mmol, 1.25 eq), NaO*t*Bu (50 mg, 0.52 mmol, 1.5 equiv.), $Pd_2(dba)_3$ (3.2 mg, 3.5 µmol, 1 mol%), PNp_3 (1.7 mg, 7 µmol, 2 mol%) in distilled toluene (0.7 mL). Filtration over a plug of silica (SiO_2, petroleum ether) yielded the title compound as a yellow oil (38 mg, 0.11 mmol, 30%).

δ^{1}H (500 MHz, $CDCl_3$) 7.80 (1 H, d, *J* = 8.1 Hz, H-14), 7.76 (1 H, d, *J* = 8.6 Hz, H-16), 7.40 (1 H, d, *J* = 2.2 Hz, H-4), 7.39 (2 H, m, H-18 + H-10), 7.27 (2 H, m, H-17 + H-9), 7.02 (1 H, d, *J* = 2.1 Hz, H-6), 5.82 (1 H, s, NH), 2.14 (3 H, s, H-19), 1.69 (3 H, s, H-20), 1.58 (9 H, s, H-15), 1.38 (9 H, s, H-22).

δ^{13}C (126 MHz, $CDCl_3$) 145.6 (C-5), 140.6 (C-3), 139.3 (C-1), 138.1 (C-2), 133.7 (C-11), 132.5 (C-12), 130.4 (C-9), 128.5 (C-14), 126.7 (C-7), 126.5 (C-6), 124.9 (C-18), 124.8 (C-17), 122.4 (C-14), 121.8 (C-4), 121.1 (C-8), 120.2 (C-10), 35.5 (C-15), 34.6 (C-21), 31.7 (C-22), 31.2 (C-15), 20.0 (C-20), 19.5 (C-19).

IR (film, cm^{-1}) ν_{max} 3465 (N–H), 2953 (C–H), 2867 (C–H), 1567, 1481;

HRMS (APCI$^+$) *m/z* calcd for $C_{26}H_{34}N$ $[M+H]^+$ 360.2691, found 360.2682.

2,6-diisopropyl-*N*-methyl-*N*-(o-tolyl)aniline - 6g'

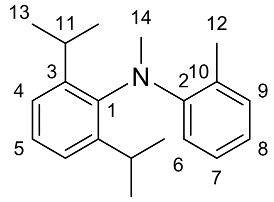

To a solution of compound **6g** (53 mg, 0.20 mmol, 1.0 equiv.) in DMF (2.0 mL, 0.1 M) at 0 °C was added NaH (60% suspension in mineral oil, 16 mg, 0.40 mmol, 2.0 equiv.) followed by iodomethane (33 μL, 0.40 mmol, 2.0 equiv.). The reaction mixture was allowed to warm up to rt, stirred for 18 h, then quenched with an aqueous solution of saturated $NaHCO_3$ and extracted with ethyl acetate. The organic phases were combined, washed with brine, dried over $MgSO_4$ and concentrated *in vacuo*. Purification by flash column chromatography (SiO_2, petroleum ether) yielded the title compound as a colourless oil (28 mg, 50%).

δ^1H (400 MHz, DMSO-d_6) 7.28 – 7.20 (1 H, m, $C_{Ar}H$), 7.20 – 7.06 (3 H, m, $C_{Ar}H$), 7.02 – 6.76 (2 H, m, $C_{Ar}H$), 6.67 (1 H, td, *J* = 7.3, 1.2 Hz, $C_{Ar}H$), 3.16 (3 H, s, NCH_3), 3.08 (2 H, sept, *J* = 5.9 Hz, $CH(CH_3)_2$), 1.47 (3 H, br. s, CCH_3), 1.14 (CH_AH_B, 6 H, d, *J* = 5.9 Hz, $CH(CH_3)_2$), 0.93 (CH_AH_B, 6 H, d, *J* = 5.8 Hz, $CH(CH_3)_2$).

δ^{13}C (126 MHz, $CDCl_3$) 147.2 (C-1), 144.6 (C-2), 132.5 (C-5 + C-9), 126.9 (C-3), 126.7 (C-7), 125.5 (C-4), 124.3 (C-10), 118.6 (C-8), 111.4 (C-13), 42.9 (C-14), 27.9 (C-11), 25.4 (C-6), 22.8 (C-6), 21.2 (C-12).

IR (film, cm^{-1}) v_{max} 2966 (C–H), 1697, 1514, 1156 (C–N);

HRMS (APCI$^+$): *m/z* calcd for $C_{20}H_{28}N$ $[M+H]^+$ 282.2222, found 282.2212.

Calculation of barriers to enantiomerisation

VT ^{1}H NMR experiments

^{1}H NMR spectra of a solution of **6d–g** in various deuterated solvent were recorded at different temperatures until coalescence was reached and line sharpening was observed. Their slow exchange spectra were simulated using the software Spinworks 4 (available at ftp://davinci.chem.umanitoba.ca/pub/marat/SpinWorks/ (accessed on 09/05/2020)), and for each temperature recorded, the corresponding rate of enantiomerisation k_{enant} was estimated using the dynamic NMR simulation module MEXICO.

From an Eyring plot, their enthalpy $\Delta H^{\neq}$ and entropy $\Delta S^{\neq}$ were calculated, and their corresponding Gibbs free energy $\Delta G^{\neq}$ were found at 25 °C. Using the following equations, their rate of exchange and half-life to enantiomerisation at 25 °C were calculated:

$$\Delta G_T^{\ddagger} = R * T * \ln\left(\frac{k_{\text{B}} * T}{h * k_{\text{enant}}}\right)$$

$$t_{1/2} = \frac{\ln(2)}{2 * k_{\text{enant}}}$$

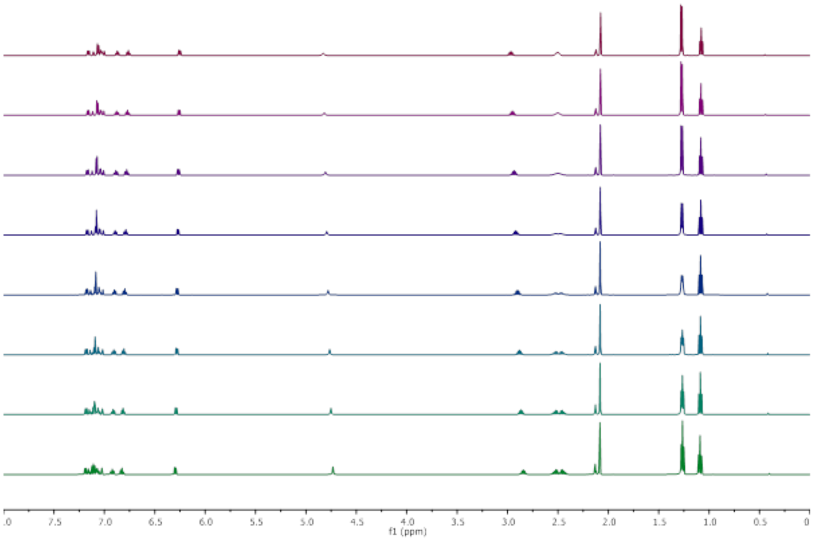

Figure S1a: Full spectrum of **6d** in toluene-*d8* (top to bottom: 60 °C, 50 °C, 40 °C, 30 °C, 20 °C, 10 °C, 0 °C, –10 °C)

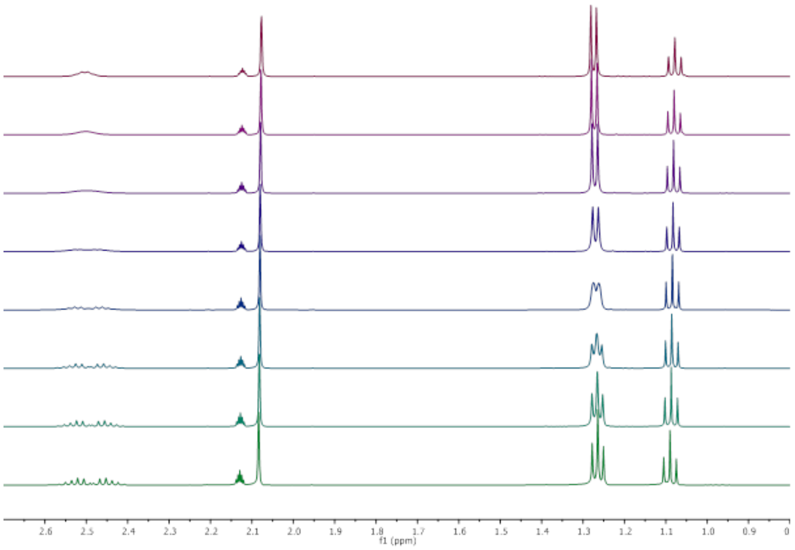

Figure S1b: Zoom on the alkyl region (2.7 – 0.8 ppm)

Calculation of the barrier to rotation of **6d** in toluene-d_8 with the coalescence of the signal centred around 1.28 ppm for the CH*Me*$_2$ protons.

Temperature (K)	k (Hz)
263	2
273	4.5
283	7.5
293	16.5
303	32
313	55
323	75

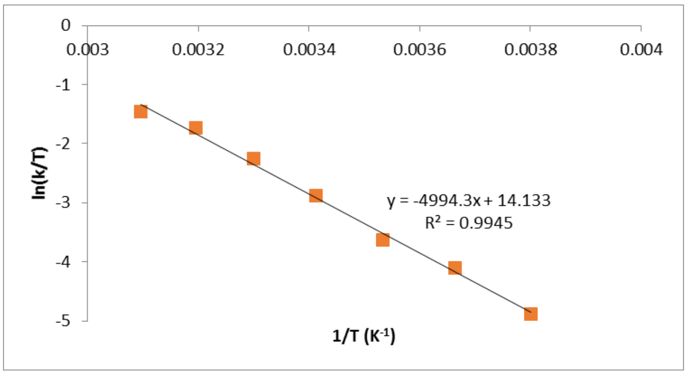

Calculation of the barrier to rotation of **6d** in toluene-d_8 with the coalescence of the signal centred around 2.49 ppm for the C*H*$_2$CH$_3$ protons.

Temperature (K)	k (Hz)
283	9
293	17
303	38
313	90
323	200
333	400

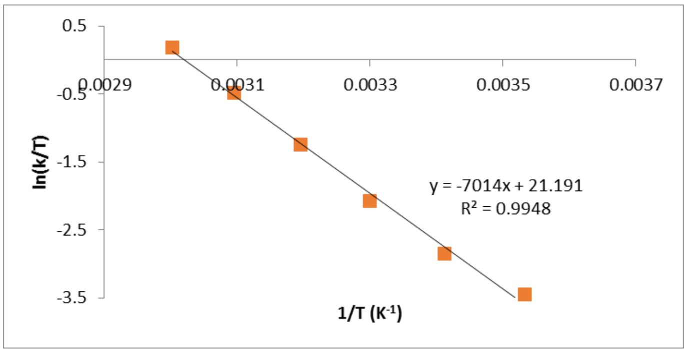

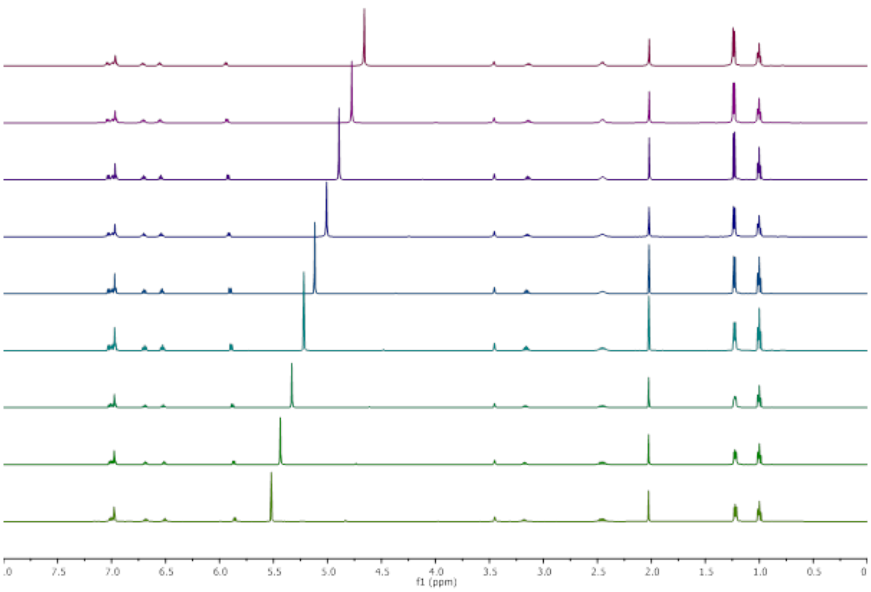

Figure S2a: Full spectrum of **6d** in C_2D_5OD (top to bottom: 70 °C, 60 °C, 50 °C, 40 °C, 30 °C, 20 °C, 10 °C, 0 °C, –10 °C)

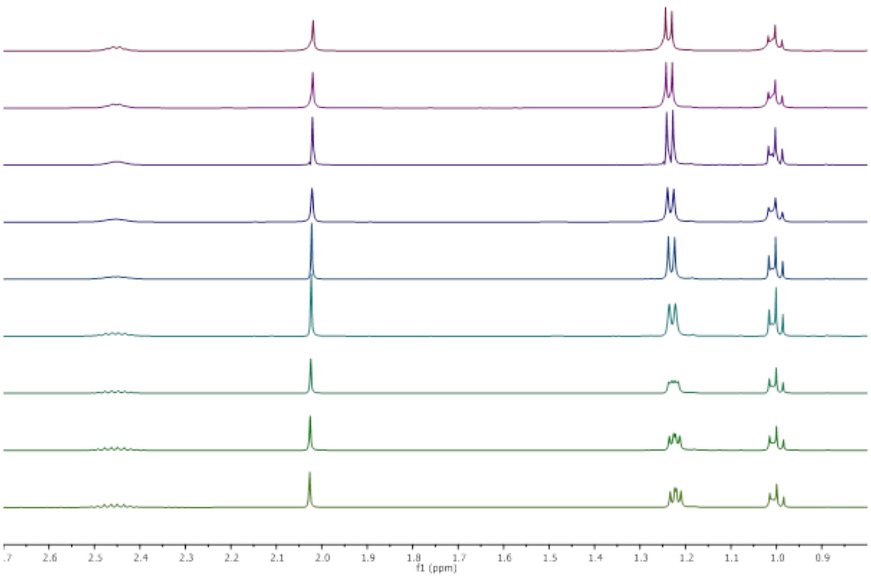

Figure S2b: Zoom on the alkyl region (2.7 – 0.8 ppm)

Temperature (K)	k (Hz)
263	3.4
273	4.9
284	8.5
293	14.8
303	26

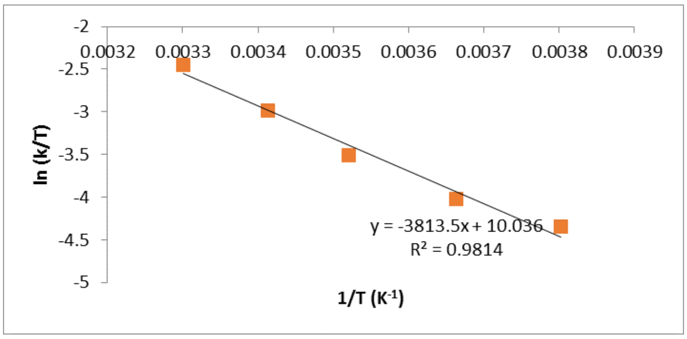

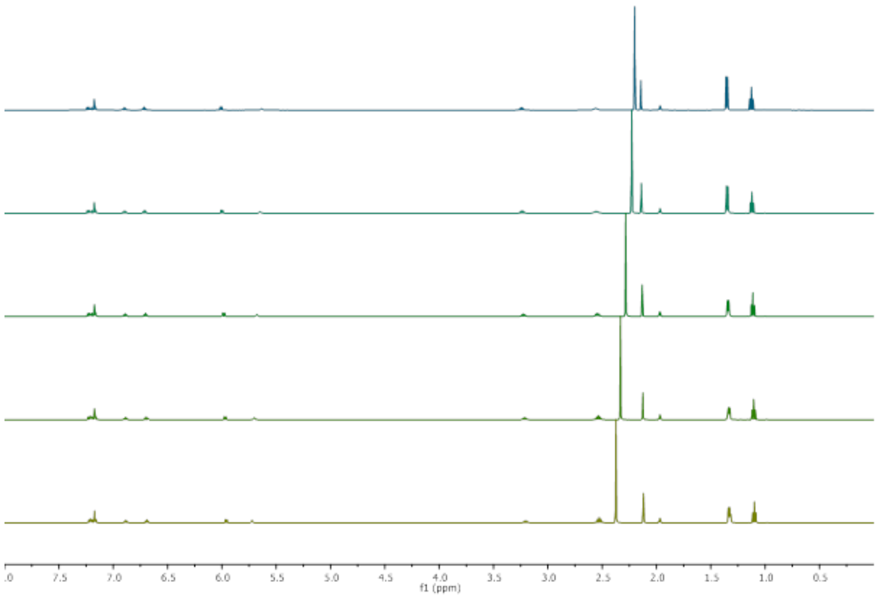

Figure S3a: Full spectrum of **6d** in CD_3CN (top to bottom: 30 °C, 20 °C, 10 °C, 0 °C, –10 °C)

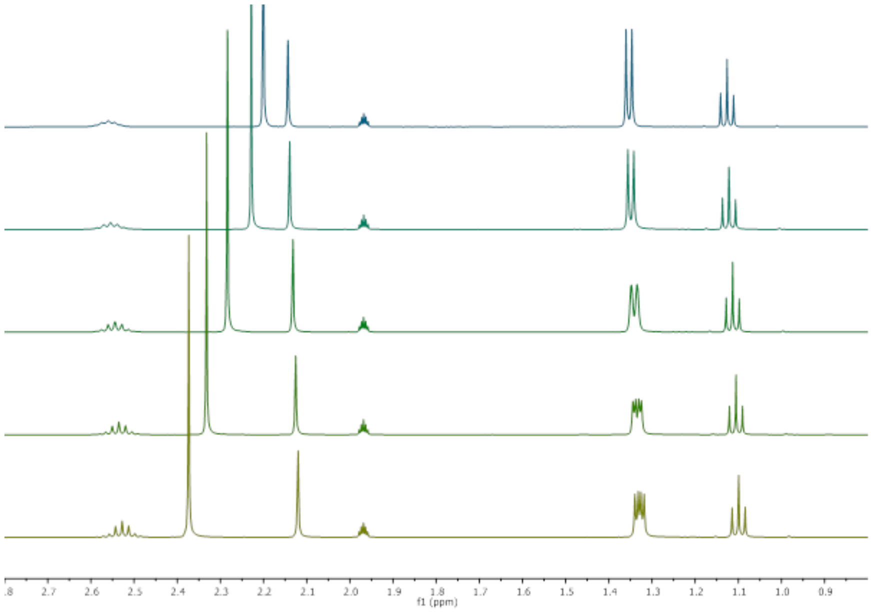

Figure S3b: Zoom on the alkyl region (2.7 – 0.8 ppm)

Temperature (K)	k (Hz)
263	3.85
273	5.8
284	10.5
295	20
303	29

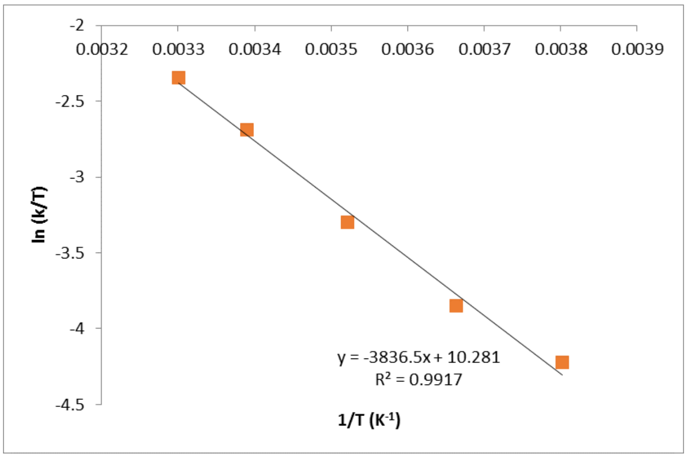

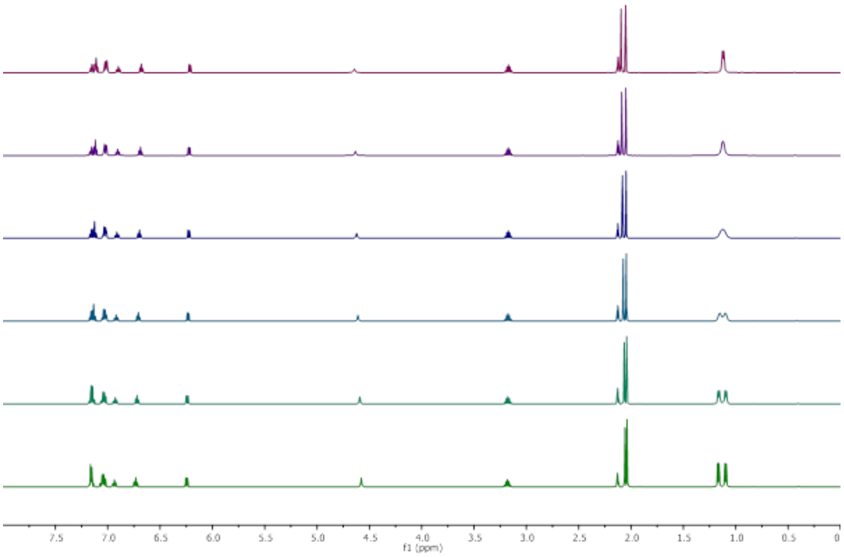

Figure S4a: Full spectrum of **6e** in toluene-*d8* (top to bottom: 50 °C, 40 °C, 30 °C, 20 °C, 9 °C, 1 °C)

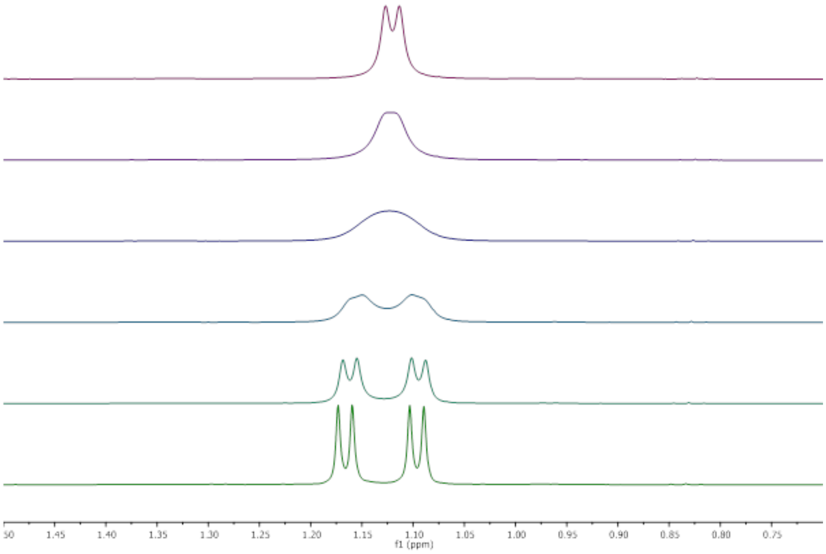

Figure S4b: Zoom on the CHMe_2 region (1.5 – 0.7 ppm)

Calculation of the barrier to rotation of **6e** in toluene-d_8 with the coalescence of the signal centred around 1.13 ppm for the CHMe_2 protons.

Temperature (K)	K (Hz)
274	6
282	12
293	40
303	100
313	240
323	490

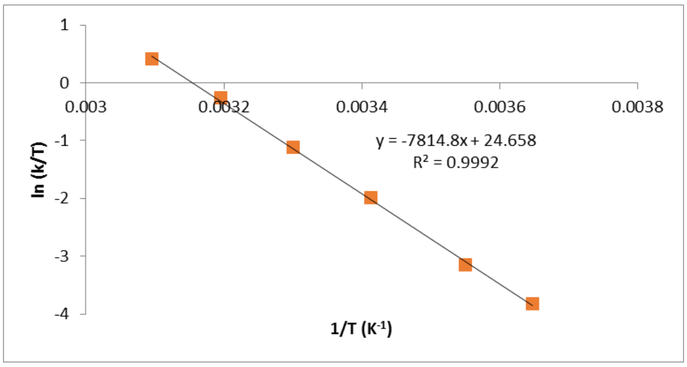

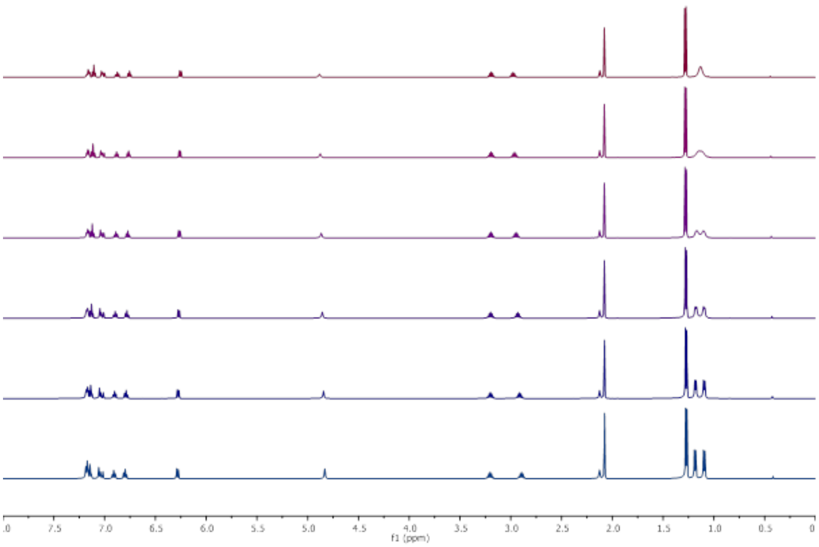

Figure S5a: Full spectrum of **6f** in toluene-*d8* (top to bottom: 60 °C, 50 °C, 40 °C, 30 °C, 20 °C, 9 °C)

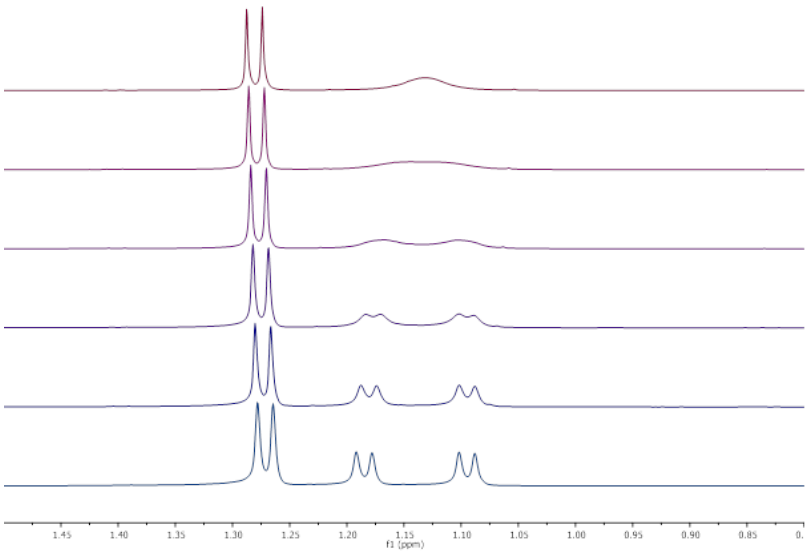

Figure S5b: Zoom on the CH*Me*$_2$ region (1.5 – 0.8 ppm)

Calculation of the barrier to rotation of **6f** in toluene-d_8 with the coalescence of the signal centred around 1.14 ppm for the CH*Me*$_2$ protons.

Temperature (K)	k (Hz)
282	8
293	11.5
303	20
313	47
323	105
333	215

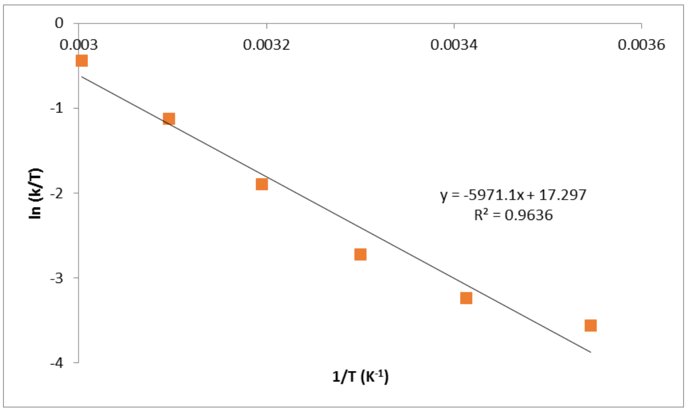

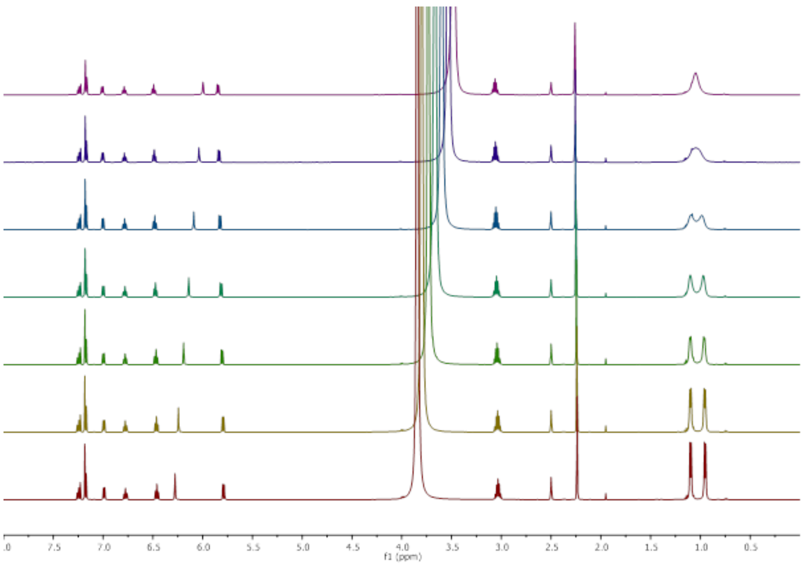

Figure S6a: Full spectrum of **6g** in DMSO-*d6* (top to bottom: 80 °C, 70 °C, 60 °C, 50 °C, 40 °C, 30 °C, 23 °C)

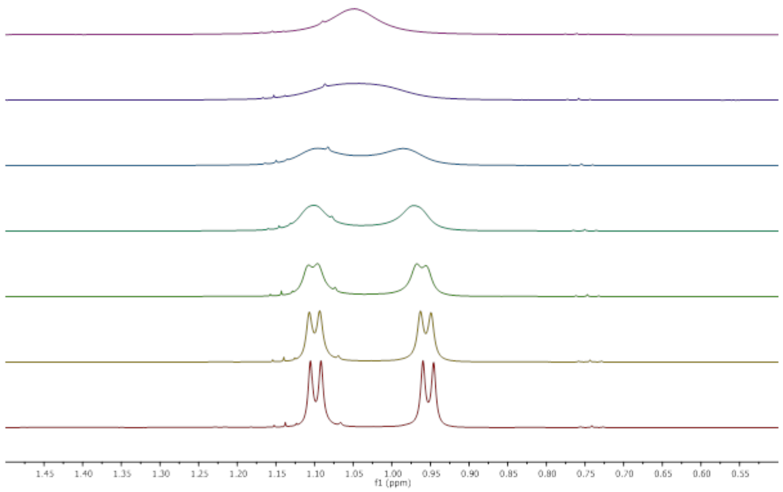

Figure S6b: Zoom on the CHMe_2 region (1.5 – 0.8 ppm)

Calculation of the barrier to rotation of **6g** in DMSO-d_6 with the coalescence of the signal centred around 1.02 ppm for the CHMe_2 protons.

Temperature (K)	k (Hz)
296	10
303	14
313	25
323	50
333	91
343	186
351	278

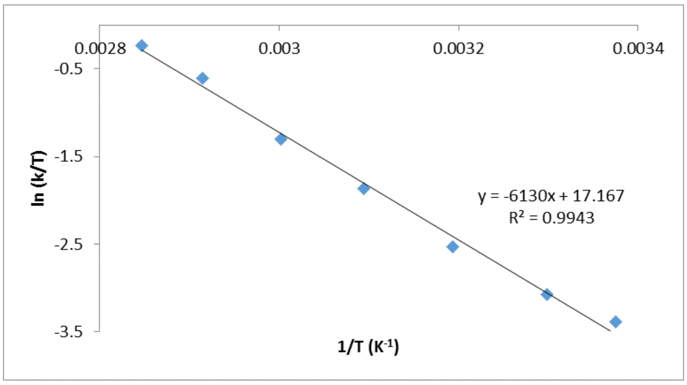

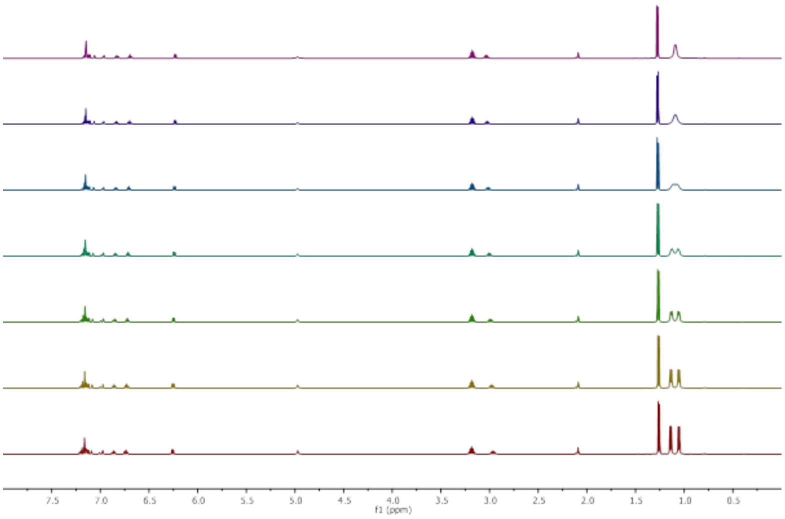

Figure S7a: Full spectrum of **6h** in toluene-*d8* (top to bottom: 90 °C, 80 °C, 70 °C, 60 °C, 50 °C, 40 °C, 30 °C)

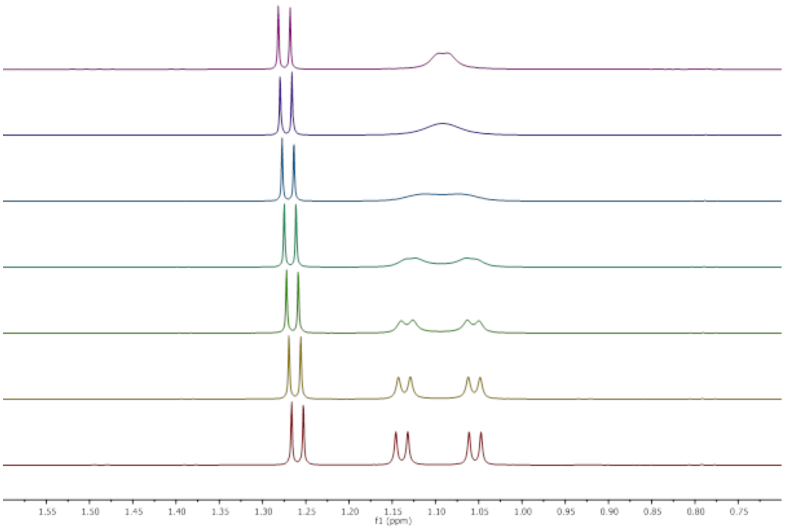

Figure S7b: Zoom on the CH*Me*$_2$ region (1.6 – 0.7 ppm)

Calculation of the barrier to rotation of **6h** in toluene-d_8 with the coalescence of the signal centred around 1.10 ppm for the CHMe_2 protons.

Temperature (K)	k (Hz)
303	5.5
313	8.5
323	16.5
333	32
343	87
353	170
363	425

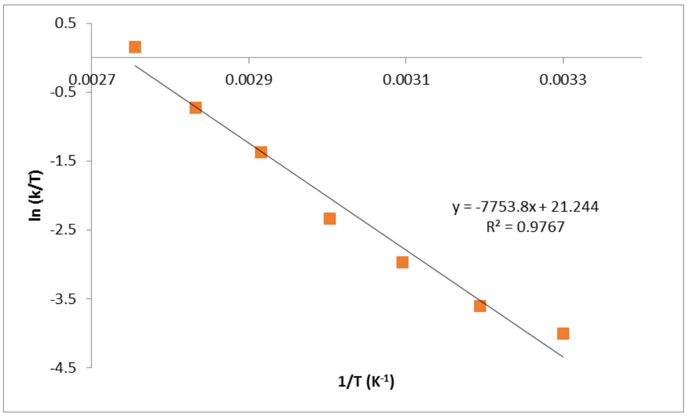

Chiral VT-HPLC experiments

Separation of both enantiomers of compounds **6i–j** and **6m–o** by chiral HPLC was performed on an Agilent 1260 Infinity. Temperature of the eluent and of the column was controlled with a ± 0.3 °C error margin.

For each temperature, the corresponding rate of exchange was calculated using the Trapp equation.[7] From an Eyring plot, their enthalpy ΔH$^{\neq}$ and entropy ΔS$^{\neq}$ were calculated, and their corresponding Gibbs free energy ΔG$^{\neq}$ were found at 25 °C. Using the following equations, their rate of exchange and half-life to enantiomerisation at 25 °C were calculated:

$$\Delta G_T^{\ddagger} = R * T * ln\left(\frac{k_B * T}{h * k_{enant}}\right)$$

$$\tau_{1/2} = \frac{ln(2)}{2 * k_{enant}}$$

Compound **6i**:

Conditions: Daicel CHIRALCEL® OD-H, hexane/2-propanol (98:2), flow: 0.8 mL/min.

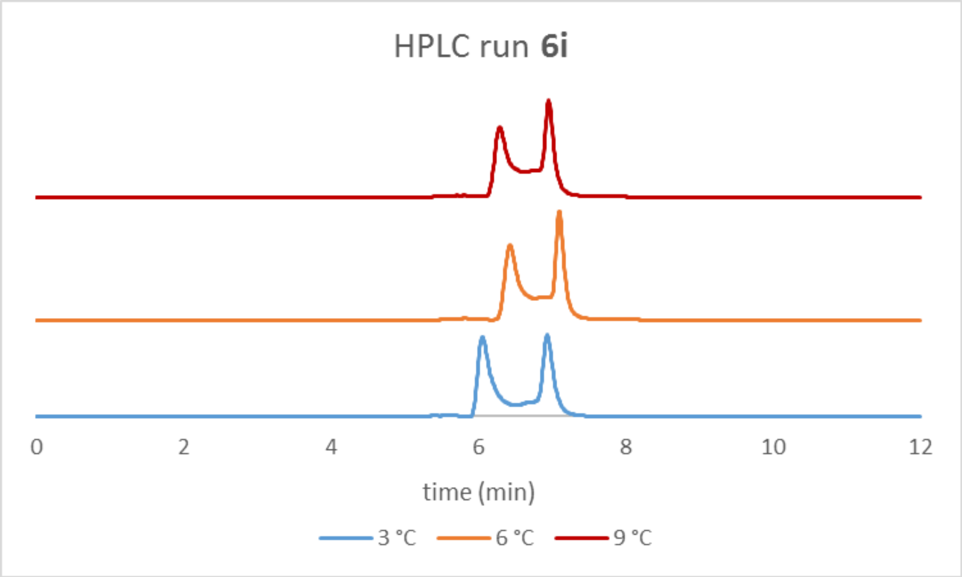

Figure S8: VT-HPLC trace of compound **6i**

Temperature (K)	k (Hz)
276	9.41 10^{-4}
279	1.18 10^{-3}
281	1.45 10^{-3}

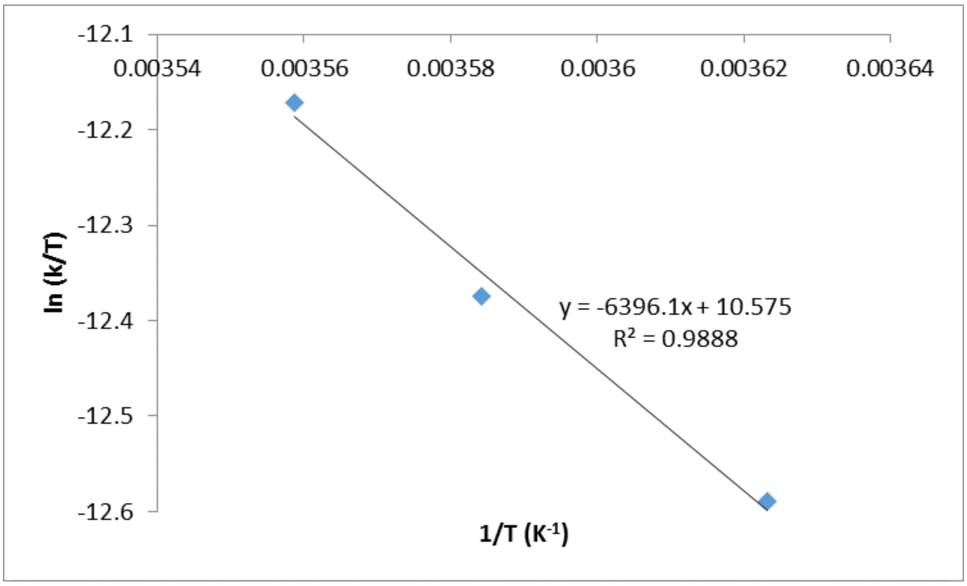

Compound **6j**:

Conditions: Daicel CHIRALCEL® OD-H, hexane/2-propanol (99.8:0.2), flow: 0.8 mL/min.

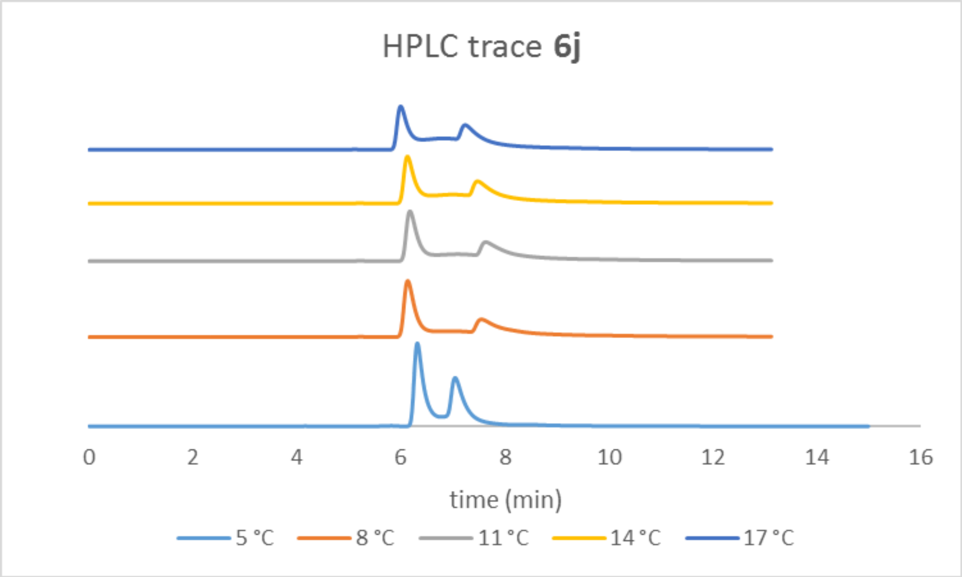

Figure S9: VT-HPLC trace of compound **6j**

Temperature (K)	k (Hz)
278	4.87 10^{-4}
281	5.86 10^{-4}
284	7.74 10^{-4}
287	1.00 10^{-3}
290	1.25 10^{-3}

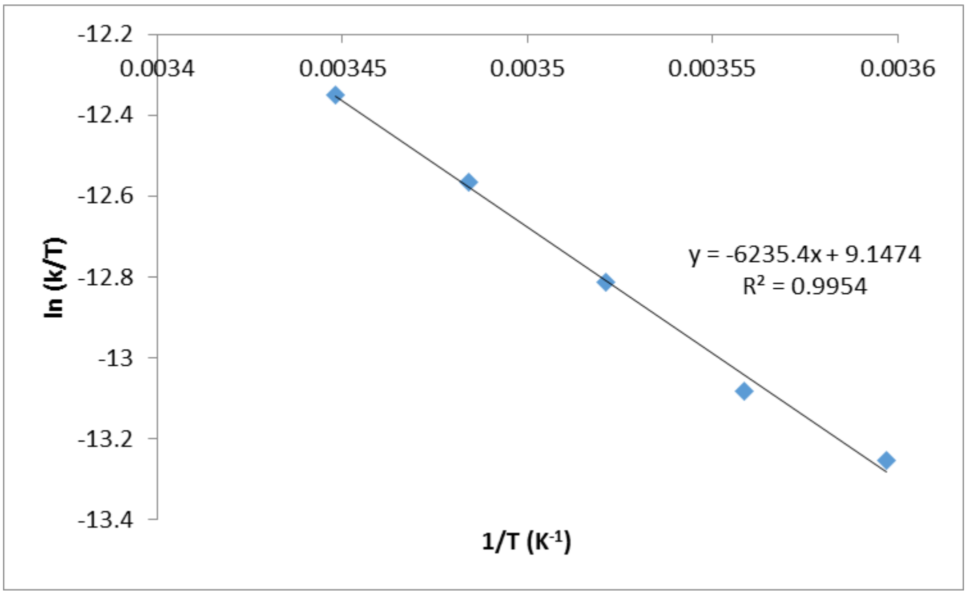

Compound **6m**:

Conditions: Daicel CHIRALCEL® OD-H, hexane, flow: 0.8 mL/min.

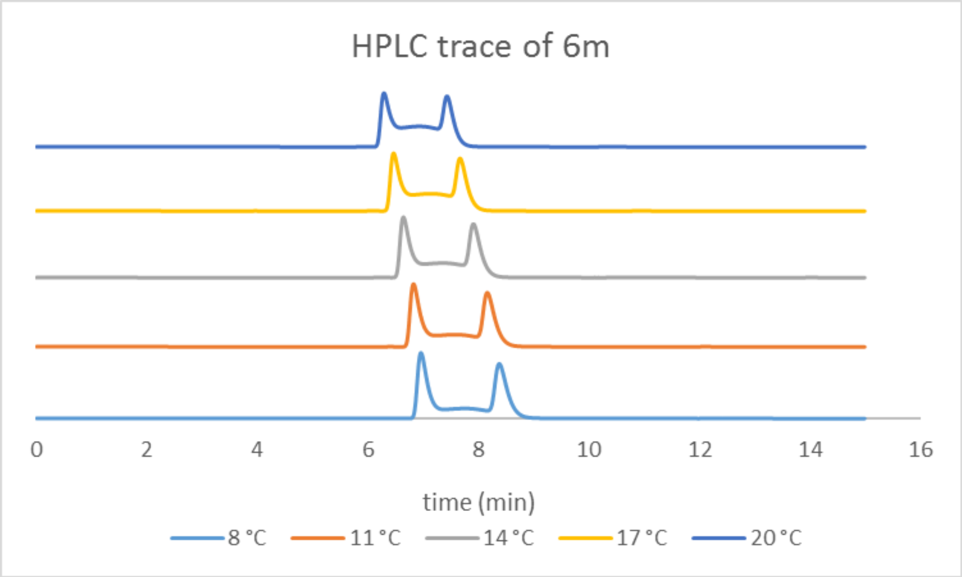

Figure S10: VT-HPLC trace of compound **6m**

Temperature (K)	k (Hz)
281	1.04 10^{-3}
284	1.25 10^{-3}
287	1.48 10^{-3}
290	1.79 10^{-3}
293	2.11 10^{-3}

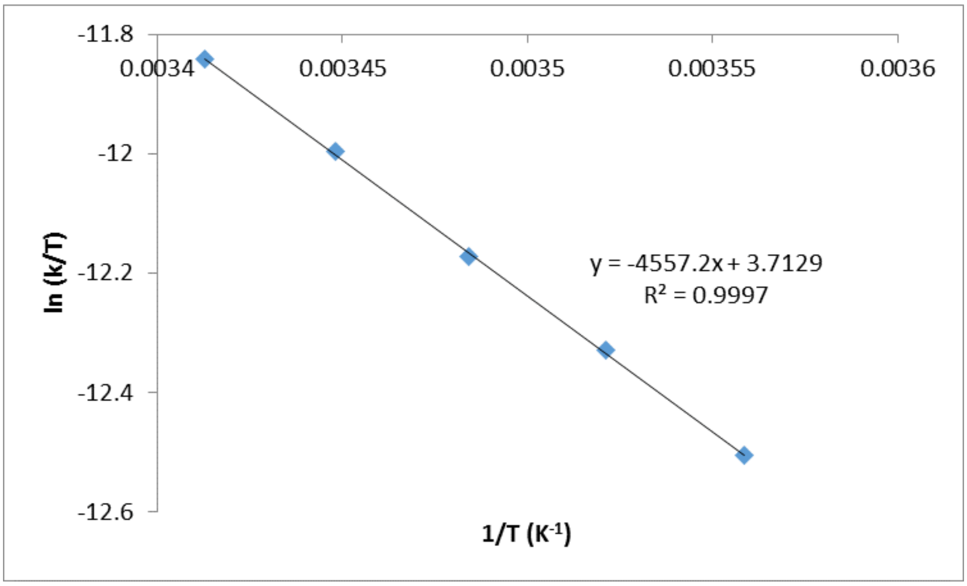

Compound **6n**:

Conditions: Daicel CHIRALCEL® OD-H, hexane/ethyl acetate (99:1), flow: 0.8 mL/min.

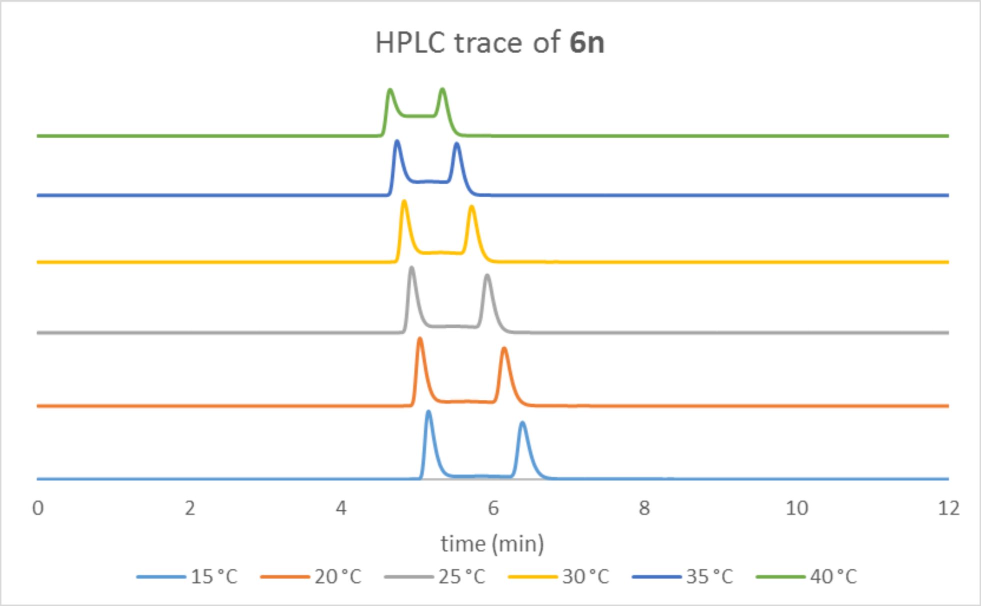

Figure S11: VT-HPLC trace of compound **6n**

Temperature (K)	k (Hz)
288	5.91 10^{-4}
293	8.05 10^{-4}
298	1.07 10^{-3}
303	1.47 10^{-3}
308	1.96 10^{-3}
313	2.45 10^{-3}

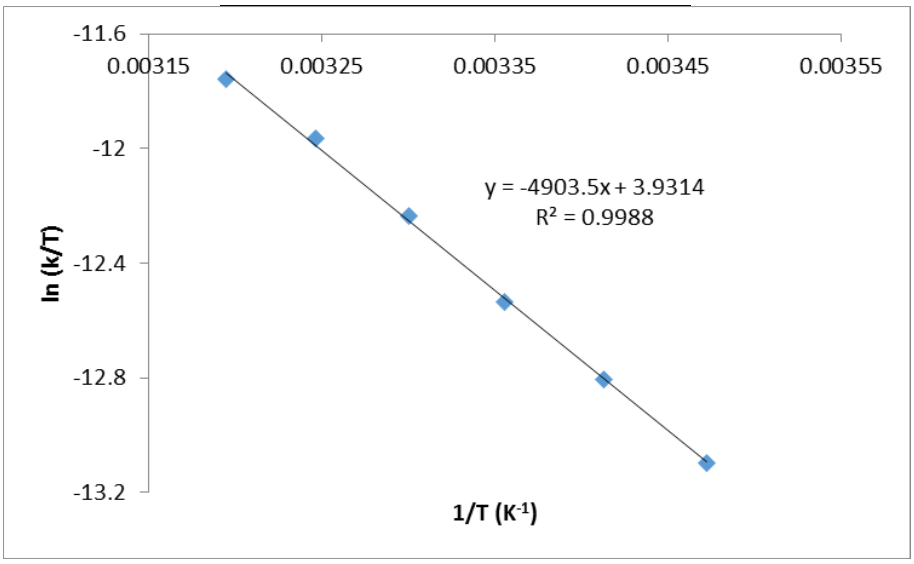

Compound **6o**:

Conditions: Daicel CHIRALCEL® OD-H, hexane/ethyl acetate (99:1), flow: 0.8 mL/min.

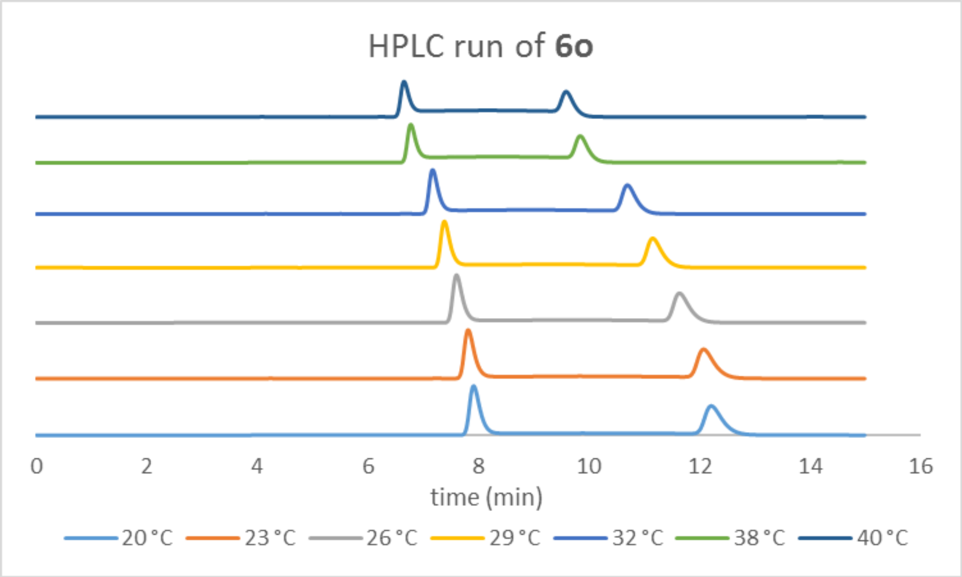

Figure S12: VT-HPLC trace of compound **6o**

Temperature (K)	k (Hz)
293	6.98 10^{-4}
296	8.77 10^{-4}
299	1.04 10^{-3}
302	1.25 10^{-3}
305	1.53 10^{-3}
311	2.17 10^{-3}
313	2.46 10^{-3}

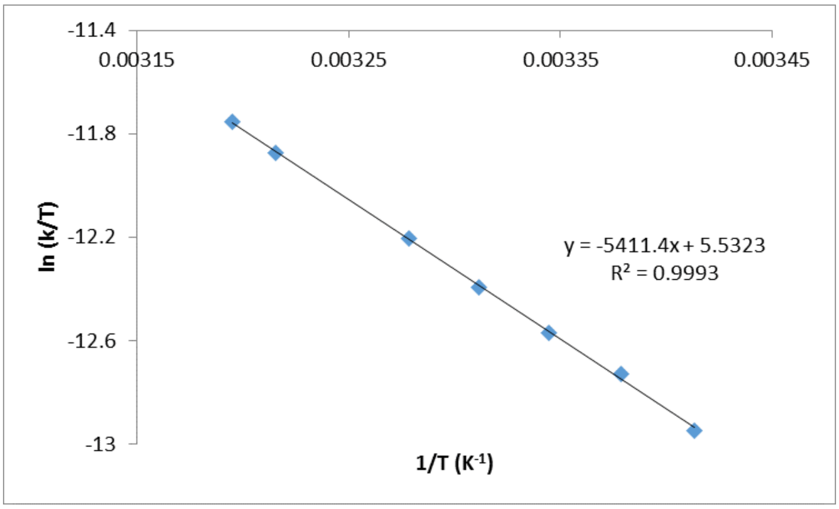

Decay of enantiomeric excess of enantioenriched samples

Separation of both enantiomers of compounds **6s** and **6t** by chiral HPLC was performed on an Agilent 1260 Infinity. The pure fractions of each enantiomer were combined, diluted in toluene and left at room temperature or heated to 100 °C for **6s** and **6t** respectively. Aliquots of the solution were taken, diluted in hexane and ran on HPLC using the conditions stated below.

The ln of the enantiomeric excess was plotted against time, and their rate of racemisation k_{rac} was obtained with the slope of the trendline. (Caution!: k_{rac} = 2* k_{enant}).

Using the following equations, their Gibbs free energy and half-life to enantiomerisation were calculated at 25 °C and 100 °C respectively using:

$$\Delta G_T^{\ddagger} = R * T * ln\left(\frac{k_B * T}{h * k_{enant}}\right)$$

$$\tau_{1/2} = \frac{ln(2)}{2 * k_{enant}}$$

Compound **6s**:

Conditions: Daicel CHIRALCEL® OD-H, hexane/2-propanol (99.5:0.5), 1.0 mL/min,

t_R = 11.2 and 12.3 min.

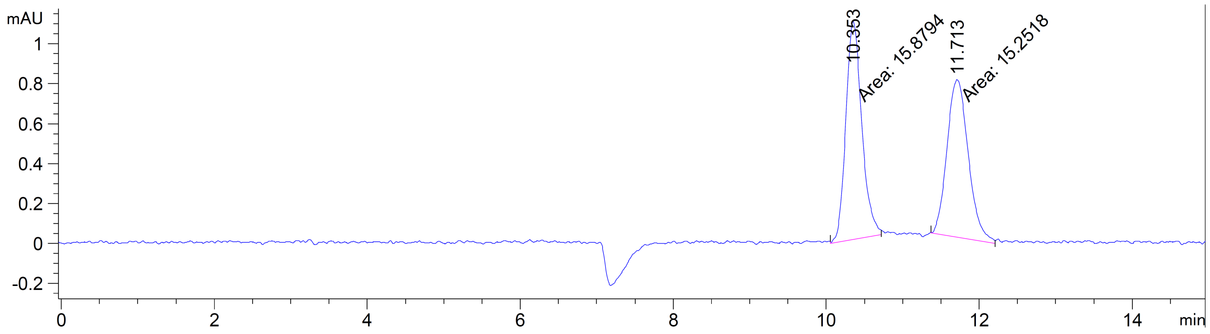

Figure S13: HPLC trace of compound **6s**

Time (min)	*ee* (%)
5	79.8
20	56.0
35	38.6
50	33.4
65	17.5
80	11.5

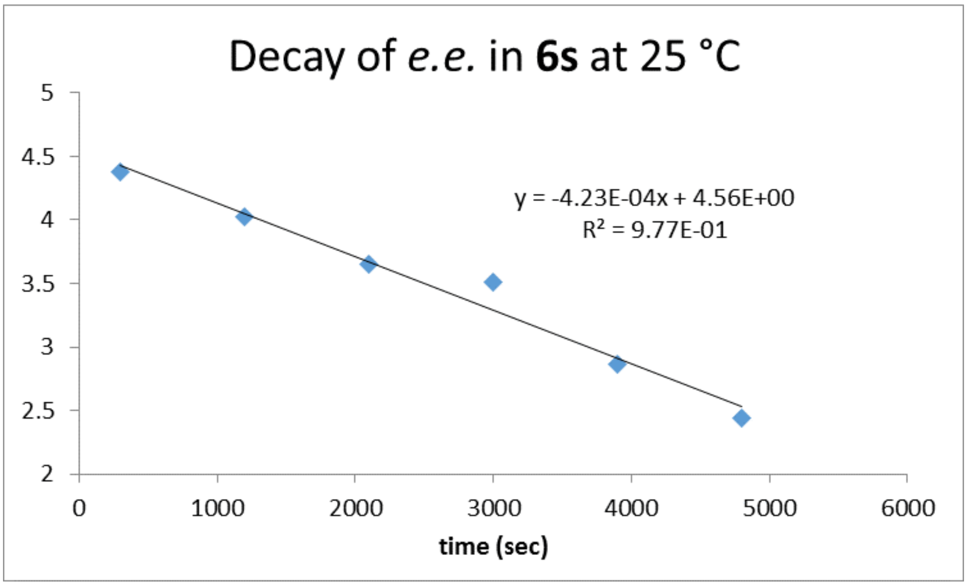

Compound **6t**:

Conditions: Daicel CHIRALCEL® OD-H, hexane, flow: 1.0 mL/min, t_R = 13.9 and 15.8 min.

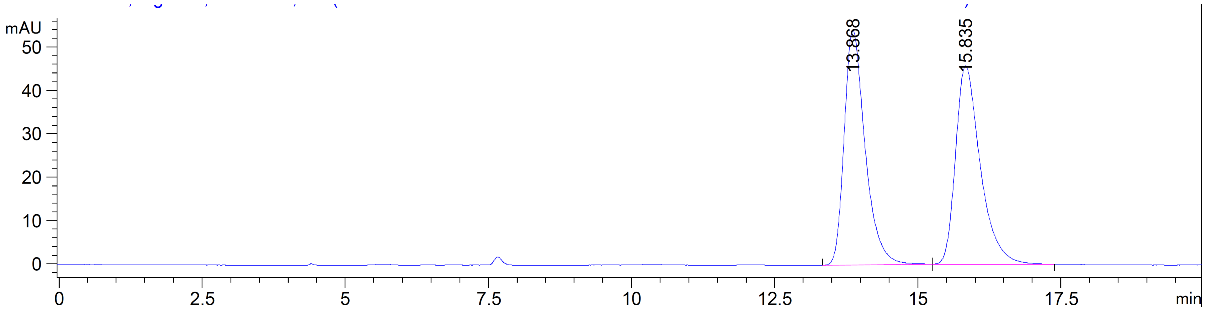

Figure S14: HPLC trace of compound **6t**

**Time (h)**	***ee* (%)**
0	98.6
7	72.4
14	56.2
19	47.0

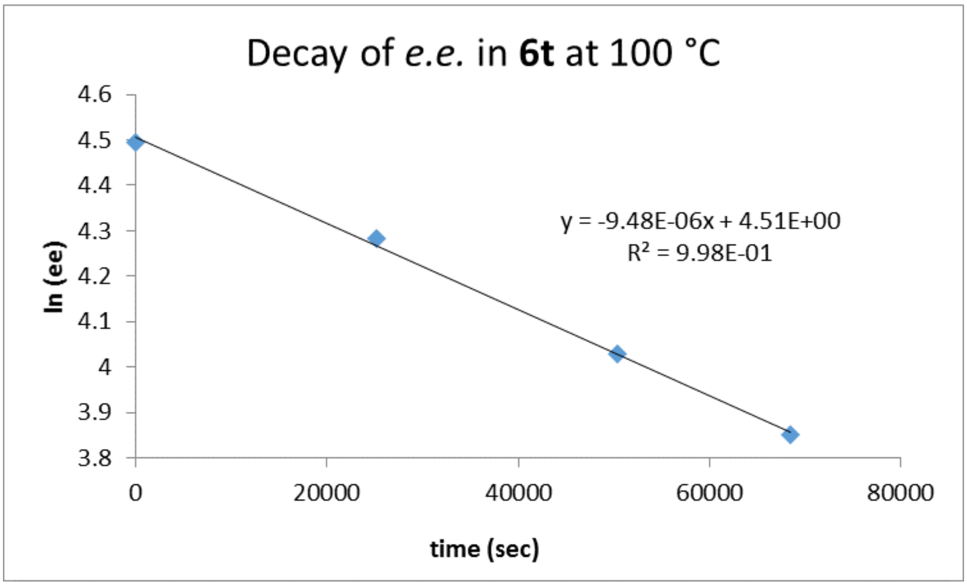

Comparison of barriers to interconversion with similar diaryl ethers.[8]

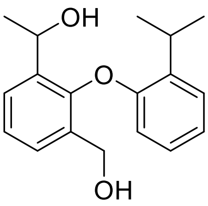

$\Delta G^{\ddagger}_{25\ ^{\circ}C}$ *n/a* ; $t_{1/2}$ = 2 s

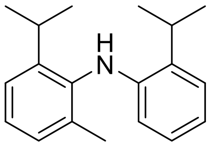

6f

$\Delta G^{\ddagger}_{25\ ^{\circ}C}$ = 65.8 kj/mol ; $t_{1/2}$ = 18 ms

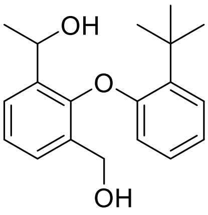

$\Delta G^{\ddagger}_{25\ ^{\circ}C}$ = 105.0 kj/mol ; $t_{1/2}$ = 20 h

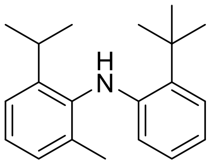

6j

$\Delta G^{\ddagger}_{25\ ^{\circ}C}$ = 88.0 kj/mol ; $t_{1/2}$ = 2.5 min

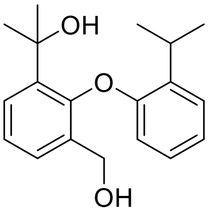

$\Delta G^{\ddagger}_{25\ ^{\circ}C}$ = 90.9 kj/mol ; $t_{1/2}$ = 8 min

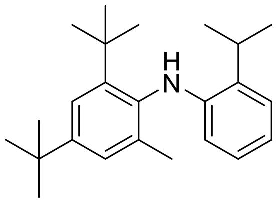

6n

$\Delta G^{\ddagger}_{25\ ^{\circ}C}$ = 89.9 kj/mol ; $t_{1/2}$ = 5.3 min

Computational Details

MD Simulations

Molecular dynamics (MD) simulations were performed in explicit toluene solvent using the Gromacs version 2016.4 package[9,10] and the PLUMED 2.4 plugin.[11] For the substrates studied computationally, OPLS-AA-compatible force-field parameters were generated using the Maestro 2015 release, force-field version 14 (Schrödinger LCC).[12] The topology for toluene was taken from the GROMACS Molecule & Liquid Database.[13] Long-range electrostatic interactions were treated using the particle mesh Ewald (PME)[14] approach with a cutoff length of 1.0 nm. A dispersion correction was applied to energy and pressure terms to account for truncation of van der Waals terms. H-bond lengths were constrained to their equilibrium length with the LINCS algorithm.

The substrates were immersed in a box of solvent with a distance from the border of at least 15 Å. Following steepest descent minimization, the systems were equilibrated in two steps, the first phase involved 500 ps of MD simulation under a constant volume and temperature (NVT) ensemble with position restraints applied to heavy atoms. The temperature was maintained at 298 K using the V-Rescale method.[15] This was followed by 1 ns of constant-pressure (NPT) equilibration utilizing the Parrinello–Rahman pressure coupling[16] algorithm with the compressibility set to 4.5×10^{-5} bar^{-1} and the time constant set to 2 ps. A 1 fs time step was used during these stages to allow potential inhomogeneities to self-adjust.

Exploration of the conformational free energy surface (FES) was performed using Metadynamics.[17] Here, biases were deposited along two sets of collective variables (CVs) using the torsional angles Ψ and Φ involved in axial chirality. They are defined as the dihedral angles between atoms 1, 2, 3 and 5, and 2, 3, 5 and 14, respectively (Figure S15). *N*-aryl substituents rings are distinguished by roman numerals **I** and **II**.). The initial Gaussian height was 1.2 kcal mol^{-1}, the width was 0.35 rad, and the deposition stride was 10 ps. The total simulation time was 20 ns. Each calculation was run in quadruplicate. Thus, leading to 80 ns of cumulative simulation time per system.

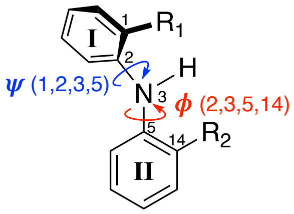

Figure S15. Definitions of dihedral angles ψ and ϕ, and aryl rings **I** and **II** used in this work.

DFT Calculations

To obtain a more accurate evaluation of energies associated to key minima and transition states (TS) identified in the 2D-FES, DFT calculations were performed on each of those states for **6a**, **6d**, **6j** and **6t**. Geometry optimizations were carried out with the M06-2X functional[18] and the 6-31+G* basis set. Solvation effects were included by an SMD description of toluene.[19] Vibrational frequencies were computed at the same level of theory to confirm whether the structures correspond to a minimum or a transition state and to evaluate the zero-point vibrational energy (ZPVE) and thermal corrections at 298 K. Vibrational entropies were corrected according to the so-called “quasi-harmonic approach”[20] using a free-rotor approximation for vibrational modes below 100 cm^{-1} and a rigid rotor approximation above this cutoff.[21] For both minima and TSs, different conformations were calculated. NCI surfaces were generated using Multiwfn (v3.6, high quality grid). Second order perturbation theory analysis of the Fock matrix from NBO (v3.1) was used to quantify $E^{(2)}$ energies for *N* lone pair donation to each aryl group. High-level single point energy evaluations were carried out using the ORCA suite of programs (version 4.1.1)[9] at the SMD(toluene)-DLPNO-CCSD(T)/def2-TZVPP level of theory. To speed up the calculations, the resolution-of-identity chain-of-spheres exchange (RIJCOSX) approximation was employed[10] with def2/J, def2-TZVPP/C auxiliary basis sets for the calculation of Coulomb and correlation integrals. The integration grids Grid6 and GridX6 were employed. Tight PNO cut-offs were found to be necessary to obtain chemical accuracy.

LFER Analysis

Given the effect of steric repulsion on the geometry of both the ground state and transition state, we hypothesised that a relationship between the steric bulk of each substituent and the barrier to isomerisation could be generated. Among the steric descriptors considered, we employed Charton values[22,23] which were found to work best for the small dataset considered here. Charton values are derived from Taft steric parameters, which provide information about substituent-effects on the rate of the hydrolysis of methyl esters.[24] The parameters for the substituents considered here were obtained from the literature and are tabulated in Table S1. No parameter has been defined for the 'benzo' substituent, so this value was estimated from the calculated structure using $\nu_{R1} = r_{VX} - r_{VH}$. With these data, the following model was obtained:

$$\Delta G^{\ddagger}_{pred} = 60.7(\pm 8.9)\ \nu_{R1} + 55.7(\pm 8.8)\ \nu_{R2} + 53.2(\pm 7.9)\ \nu_{R3} + 26.6(\pm 11.4)\ \nu_{R4} - 49.0(\pm 13.1)$$

The standard error is 6.7 kJ mol^{-1}, and the adjusted $R^2 = 0.93$ (N = 13). This model allows the prediction of the missing data values (Table S1).

Table S1. Experimental and calculated barriers to isomerisation, and Charton analysis for the compounds studied here.

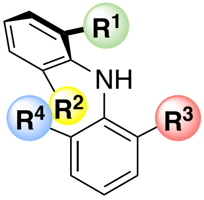

R_1, R_3 = large
R_2, R_4 = small

	ΔG$_{expt}$ / kJ mol^{-1}	ΔG$_{calc}$* / kJ mol^{-1}	Charton parameters (ν)[22–24] R$_1$	R$_2$	R$_3$	R$_4$	ΔG$_{pred}$ / kJ mol^{-1}	ΔG$_{pred-expt}$ / kJ mol^{-1}
**6a**		21.6	0.76	0.00	0.52	0.00	24.8	3.2
**6b**			1.24	0.00	0.76	0.00	66.7	
**6c**			0.52	0.52	0.76	0.00	52.0	
**6d**	65.7	63.4	1.24	0.00	0.76	0.00	54.4	-11.3
**6e**	62.8		0.76	0.52	0.52	0.00	53.8	-9.0
**6f**	65.8		0.76	0.52	0.76	0.00	66.6	0.8
**6g**	67.3		0.76	0.76	0.52	0.00	67.2	-0.1
**6h**	70.7		0.76	0.76	0.76	0.00	80.0	9.3
**6i**	85.8		0.56	0.52	1.24	0.00	80.0	-5.8
**6j**	88.0		0.76	0.52	1.24	0.00	92.1	4.1
**6k**			0.76	0.76	1.24	0.00	105.5	
**6l**			1.07[a]	0.52	1.24	0.00	110.9	
**6m**	87.6	84.8	1.24	0.52	0.52	0.00	82.9	-4.7
**6n**	89.9		1.24	0.52	0.76	0.00	95.7	5.8
**6o**	90.2		1.24	0.52	0.57	0.00	85.6	-4.6
**6p**			1.24	1.24	1.24	0.52	175.3	
**6q**			1.07[a]	0.52	0.56	0.52	88.6	
**6r**			1.24	0.52	0.76	0.52	109.5	
**6s**	93.9		1.07[a]	0.52	0.76	0.52	99.2	5.3
**6t**	130.1	129.5	1.07[a]	0.52	1.24	0.52	124.8	-5.3

* Energy barriers for the lowest energy path obtained at the SMD(toluene)-DLPNO-CCSD(T)/def2-TZVPP/SMD(toluene)-M06-2X/6-31+G* level of theory.

[a]Estimated value

Isomerisation Pathways

General Isomerisation Model

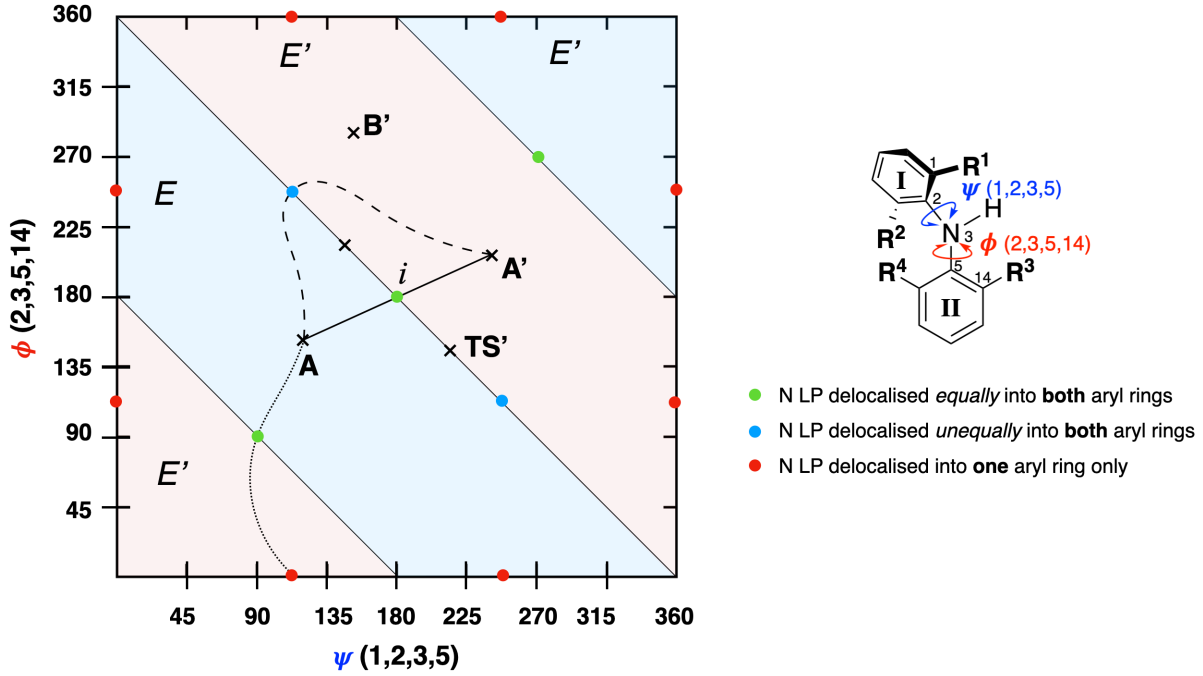

Figure S16. Model FES showing the extent of N lone pair delocalisation as a function of dihedral angles ψ and ϕ.

To undergo isomerisation, **A** must cross between valleys on the FES (Figure S16). The ease of this process depends on the minimisation of steric repulsion and maximisation of N lone pair delocalisation at the transition state. The extent of N lone pair delocalisation depends on the dihedral angles ψ and ϕ. Delocalisation is maximised at the point (180°, 180°), marked *i* in Figure S16 (green dot). The line $\psi = \phi$ indicates equal delocalisation into both aryl rings. As the extrema (0°, ϕ) and (ψ, 0°) are approached, delocalisation becomes more unequally distributed into the two aryl rings, until the lone pair is fully delocalised into one aryl ring and not localised into the other.

For the systems studied in this work, a coplanar transition state with equal delocalisation can only occur when $\mathbf{R^2}$ and $\mathbf{R^4}$ are small (*i*, green dot at (180°, 180°)). When $\mathbf{R^2}$ is large, the lowest energy transition state becomes bent, with the N lone pair delocalised unequally into both rings (blue dots). Rotation within each valley occurs through transition states where the N lone pair is delocalised only into one aryl group (red dots).

Isomerisation Pathways for 6a

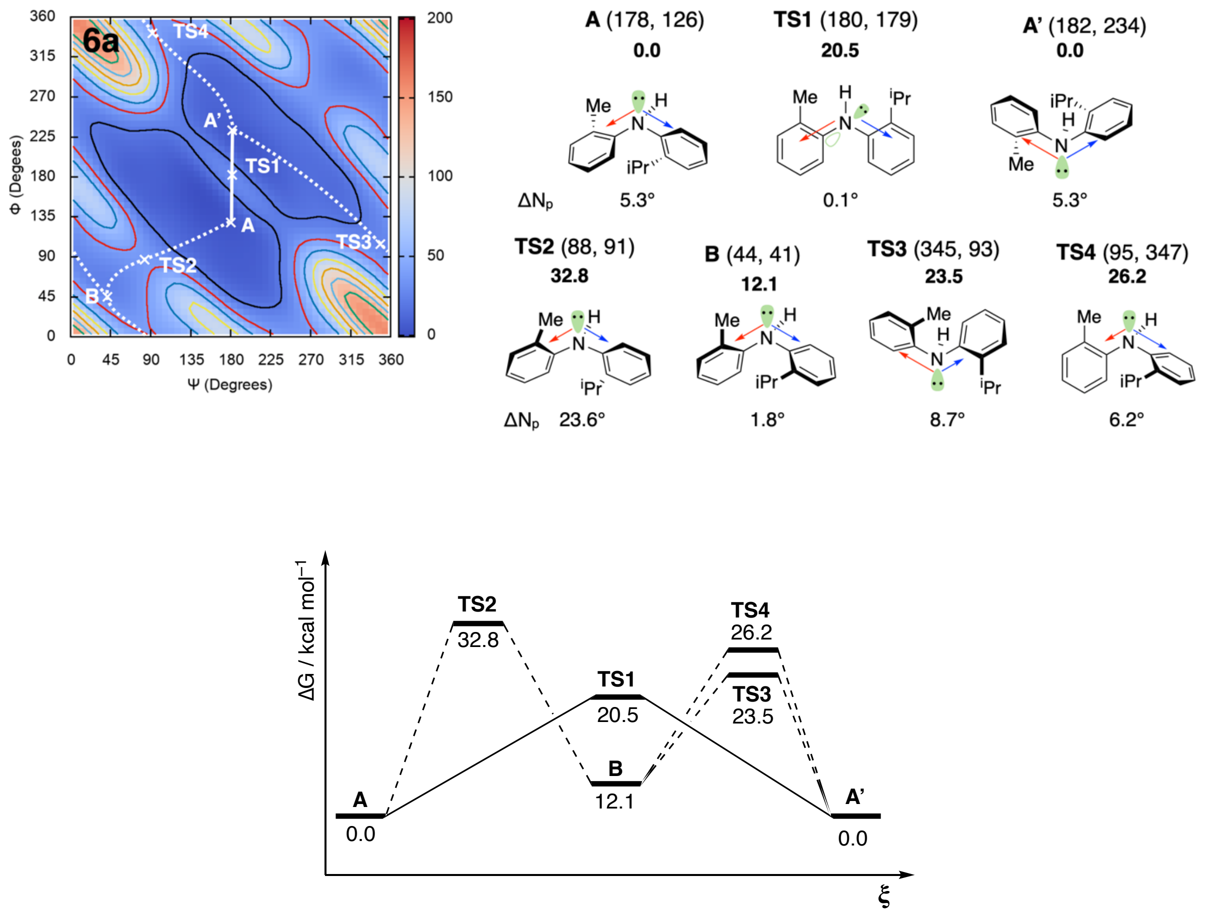

Figure S17. Classical FES in explicit toluene solvent; solid white line on the FES indicates the lowest-energy pathway; dashed lines indicate alternative isomerisation mechanisms. Nitrogen pyramidalization (ΔN_p) and relative free energies in bold calculated at the SMD(toluene)-DLPNO-CCSD(T)/def2-TZVPP//SMD(toluene)-M06-2X/6-31+G* level of theory along each isomerisation pathways and their corresponding coordinates on the FES.

Table S2. Delocalisation energy ($E^{(2)}$) of the nitrogen lone pair into each aryl ring π system, calculated at the NBO/SMD(toluene)-M06-2X/6-31+G* level of theory.

	$E^{(2)}$ [$n_N \rightarrow \pi^*$] / kJ mol^{-1}						
**Ring**	**A**	**A'**	**B**	**TS1**	**TS2**	**TS3**	**TS4**
**I** (Me)	162.7	162.7	128.7	207.5	27.6	165.5	12.6
**II** (iPr)	93.6	93.6	72.3	201.1	23.3	15.8	169.5

The lowest energy pathway linking **6a_A** and **6a_A'** involves planarisation of the molecule by rotating the isopropyl-substituted aryl group such that the C_{Ar}–Me, N–H and C_{Ar}–*i*Pr bond vectors are approximately parallel (**6a_TS1**). This maximises delocalisation of the N lone pair into both aryl groups, making it the most favoured pathway. The alternative pathway, involving concerted rotation of the methyl- and isopropyl-substituted aryl groups is higher in energy (dashed lines, Figure S17). To minimise the increase in steric clashing between *ortho*-substituents, the two aryl groups fold downwards to face each other, decreasing delocalisation of the N lone pair on the aryl groups (**6a_TS2**). The resultant metastable state **6a_B** has the C_{Ar}–Me and C_{Ar}–*i*Pr bond vectors opposing the N–H bond vector. **6a_B** undergoes inversion of configuration at N and concerted rotation of both aryl groups either through a transition state where C_{Ar}–Me and N–H are coplanar and C_{Ar}–*i*Pr is perpendicular (**6a_TS3**), or where C_{Ar}–*i*Pr and N–H are coplanar and C_{Ar}–Me is perpendicular (**6a_TS4**), the former being the lower energy pathway.

Isomerisation Pathways for 6d

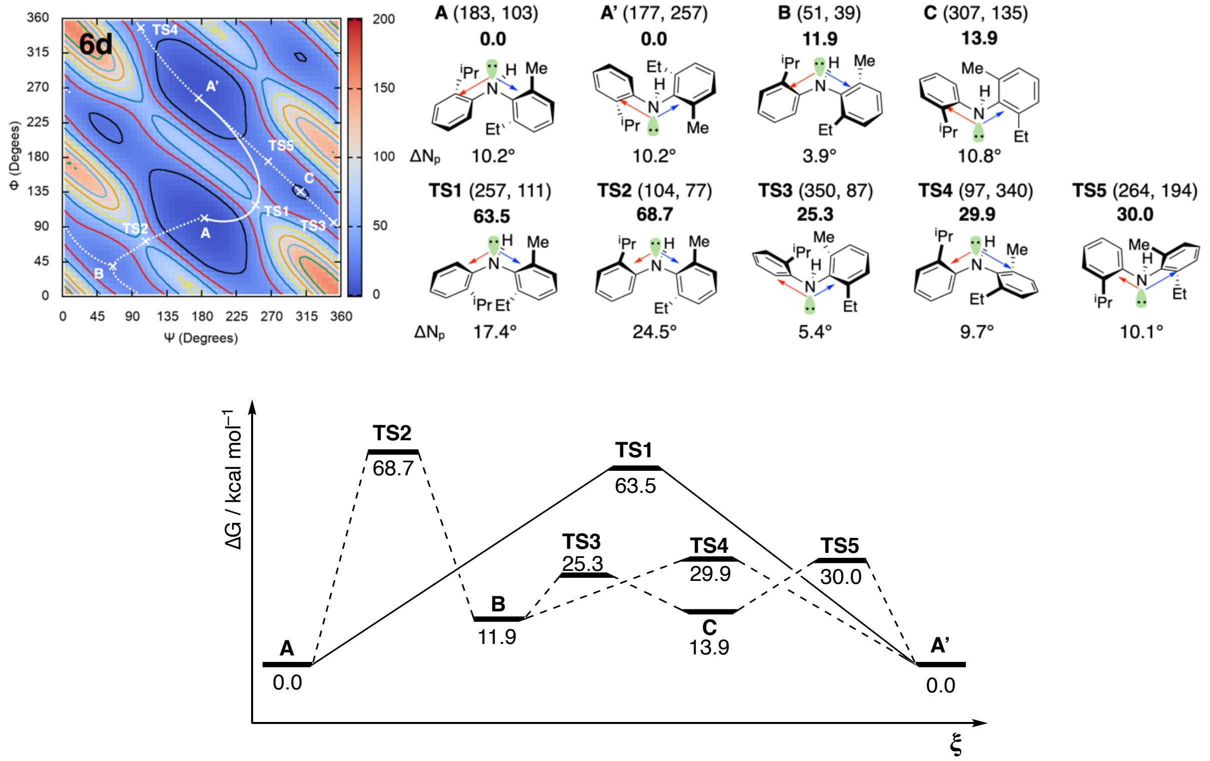

Figure S18. Classical FES in explicit toluene solvent; solid white line on the FES indicates the lowest-energy pathway; dashed lines indicate alternative isomerisation mechanisms. Nitrogen pyramidalization (ΔN_p) and relative free energies in bold calculated at the SMD(toluene)-DLPNO-CCSD(T)/def2-TZVPP//SMD(toluene)-M06-2X/6-31+G* level of theory along each isomerisation pathways and their corresponding coordinates on the FES.

Table S3. Delocalisation energy ($E^{(2)}$) of the nitrogen lone pair into each aryl ring π system, calculated at the NBO/SMD(toluene)-M06-2X/6-31+G* level of theory.

	$E^{(2)}$ [$n_N \rightarrow \pi^*$] / kJ mol^{-1}								
**Ring**	**A**	**A'**	**B**	**C**	**TS1**	**TS2**	**TS3**	**TS4**	**TS5**
**I** (*i*Pr)	43.1	43.1	111.9	106.9	53.0	8.3	175.0	15.4	13.9
**II** (Et + Me)	150.4	150.4	62.0	21.6	39.8	8.1	21.0	158.8	156.0

The direct path from **6d_A** to **6d_A’** can no longer pass through a coplanar transition state, as this will result in a steric clash between an *ortho*-substituent on the disubstituted aryl group and the *ortho*-C–H on the monosubstituted aryl group. A bent transition state similar to **6a_TS2** with face-to-face aryl groups minimises this repulsion. The C_{Ar}–Et, N–H and C_{Ar}–*i*Pr bond vectors are approximately parallel, leaving the smallest substituents (Me and H) to clash in **6d_TS1**. Increase in pyramidalization at nitrogen minimises the loss of delocalisation of the lone pair. A higher-energy pathway that instead has the C_{Ar}–Me, N–H and C_{Ar}–*i*Pr bond vectors approximately parallel leaves Et and H to clash in **6d_TS2**. Given the similar size of Me and Et, these two transition states are similar in energy ($\Delta\Delta G^{\ddagger}$ = 5.2 kJ mol^{-1}). However, while **6d_TS1** leads directly to **6d_A’**, **6d_TS2** leads to metastable **6d_B**, which must subsequently undergo a concerted rotation to return to the lowest energy conformer.

Isomerisation Pathways for 6j

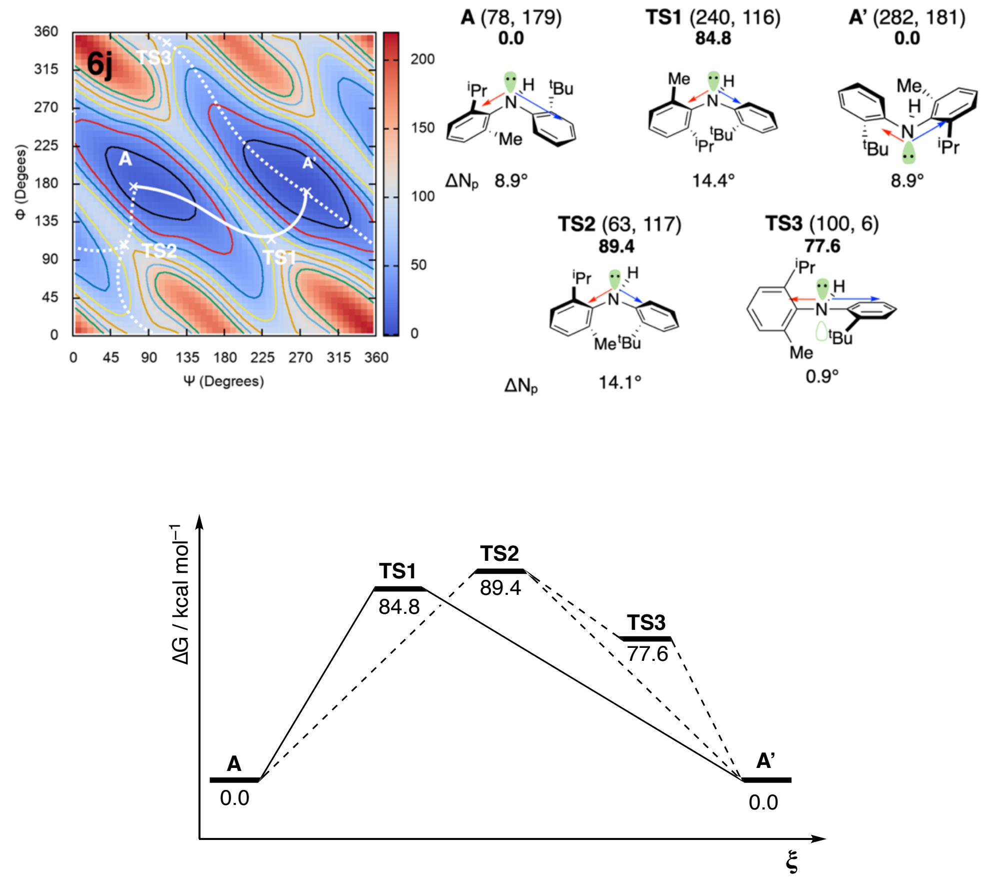

Figure S19. Classical FES in explicit toluene solvent; solid white line on the FES indicates the lowest-energy pathway; dashed lines indicate alternative isomerisation mechanisms. Nitrogen pyramidalization (ΔN_p) and relative free energies in bold calculated at the SMD(toluene)-DLPNO-CCSD(T)/def2-TZVPP//SMD(toluene)-M06-2X/6-31+G* level of theory along each isomerisation pathways and their corresponding coordinates on the FES.

Table S4. Delocalisation energy ($E^{(2)}$) of the nitrogen lone pair into each aryl ring π system, calculated at the NBO/SMD(toluene)-M06-2X/6-31+G* level of theory.

	$E^{(2)}$ [$n_N \rightarrow \pi^*$] / kJ mol^{-1}				
**Ring**	**A**	**A'**	**TS1**	**TS2**	**TS3**
**I** (Me + *i*Pr)	38.4	38.4	77.2	75.9	198.8
**II** (*t*Bu)	153.7	153.7	70.2	84.3	8.0

The direct path from **6j_A** to **6j_A’** goes via **6j_TS1**. This path involves disrotation of the two aryl groups that facilitates the formation of a bent transition state similar to that in **6d** that minimises steric repulsion between the methyl group and the *ortho*-C–H on the monosubstituted aryl group. This comes at the expense of delocalisation of the nitrogen lone pair into the monosubstituted aryl group. A second pathway that places the bulkier isopropyl group near to the *ortho*-C_{Ar}–H on the monosubstituted aryl group is only marginally higher in energy, suggesting that steric clashing between methyl and *tert*-butyl is also significant and comparable to the clash between isopropyl and *tert*-butyl.

Isomerisation Pathways for 6t

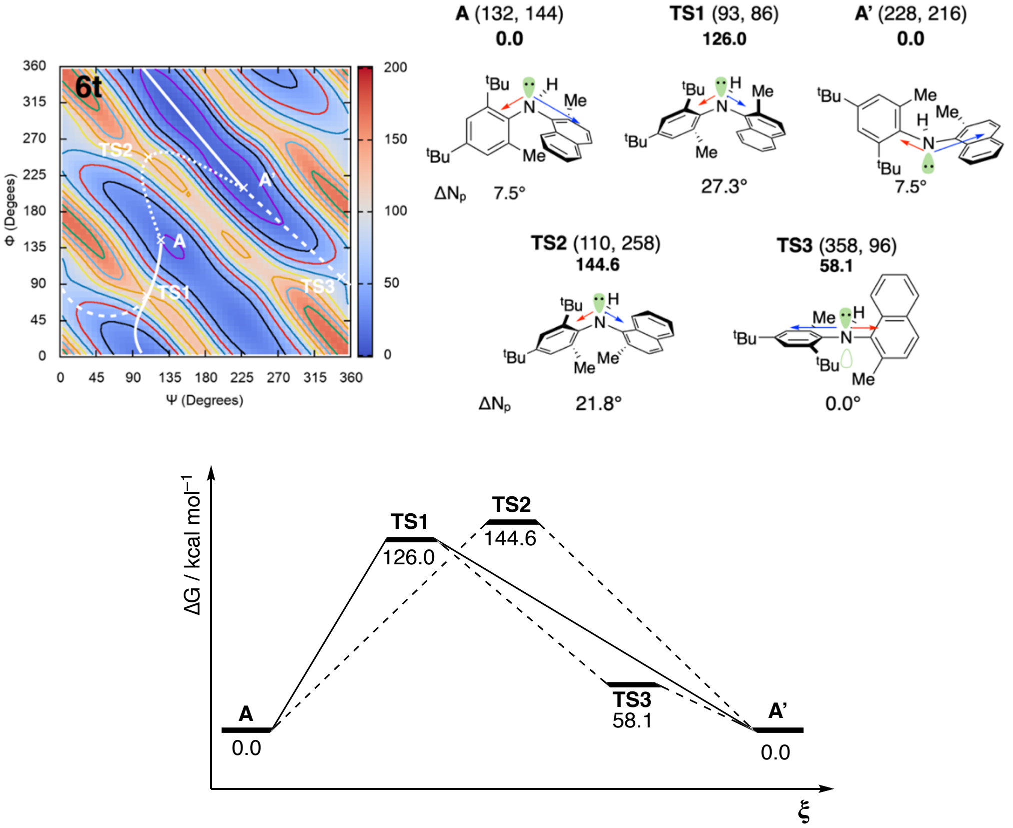

Figure S20: Classical FES in explicit toluene solvent; solid white line on the FES indicates the lowest-energy pathway; dashed lines indicate alternative isomerisation mechanisms. Nitrogen pyramidalization (ΔN_p) and relative free energies in bold calculated at the SMD(toluene)-DLPNO-CCSD(T)/def2-TZVPP//SMD(toluene)-M06-2X/6-31+G* level of theory along each isomerisation pathways and their corresponding coordinates on the FES.

Table S5. Delocalisation energy ($E^{(2)}$) of the nitrogen lone pair into each aryl ring π system, calculated at the NBO/SMD(toluene)-M06-2X/6-31+G* level of theory.

	$E^{(2)}$ [$n_N \rightarrow \pi^*$] / kJ mol^{-1}				
**Ring**	**A**	**A'**	**TS1**	**TS2**	**TS3**
**I** (Me + *t*Bu)	2.2	2.2	< 2.1	< 2.1	< 2.1
**II** (naphthyl)	154.3	154.3	5.0	13.2	3.1

In the ground state **6t_A**, the two aryl groups are perpendicular to each other, with the nitrogen lone pair almost entirely delocalised into the naphthyl arene. To undergo isomerisation, the naphthyl group twists out of conjugation to avoid steric clashing between the two pairs of *ortho*-substituents. As a result of this geometric constraint, the planes of the two aryl rings become perpendicular to the N–H bond vector at **6t_TS1** and **6t_TS2**, and nitrogen lone pair delocalisation decreases significantly. The two pathways (via **6t_TS1** or **6t_TS2**) differ only in the initial direction of the naphthyl group rotation; the lower energy path places the methyl substituent of the trisubstituted arene close to the second ring of the naphthyl group.

Table S6. Dihedral angles (ψ and ϕ), absolute electronic energies (E_{el}), zero point energy (ZPE), enthalpy (H), entropies (T.S), quasi-harmonic entropies (T.qh-S), free energies (G), quasi-harmonic Gibbs energy (qh-G), and changes in quasi-harmonic Gibbs free energies (ΔG_{rel}) for stationary points on the FES of **6a**. Geometry and thermochemistry was calculated at the SMD(toluene)-M06-2X/6-31+G* level of theory at 298.15 K and 1 M, and single point corrections were applied from the SMD(toluene)-DLPNO-CCSD(T)/def2-TZVPP level of theory with 'Tight' PNO cut-offs.

			**M06-2X/6-31+G***							**DLPNO-CCSD(T)/def2-TZVPP**		
**Structure**	ψ / °	ϕ / °	E_{el} / Ha	ZPE / Ha	H / Ha	T.S / Ha	T.qh-S / Ha	G(T) / Ha	qh-G(T) / Ha	E_{el} / Ha	qh-G(T) / Ha	ΔG_{rel} / kJ mol^{-1}
**6a_A**	178	126	-675.63864	0.31382	-675.30811	0.05712	0.05487	-675.36522	-675.36298	-674.67217	-674.39651	0.0
**6a_A'**	182	234	-675.63864	0.31382	-675.30811	0.05712	0.05488	-675.36523	-675.36298	-674.67217	-674.39651	0.0
**6a_B**	44	41	-675.63341	0.31340	-675.30328	0.05607	0.05439	-675.35935	-675.35767	-674.66762	-674.39188	12.1
**6a_TS1**	180	179	-675.63096	0.31404	-675.30091	0.05498	0.05345	-675.35589	-675.35436	-674.66530	-674.38870	20.5
**6a_TS2**	88	91	-675.62556	0.31418	-675.29582	0.05320	0.05224	-675.34902	-675.34806	-674.66152	-674.38402	32.8
**6a_TS3**	345	93	-675.63011	0.31378	-675.30055	0.05382	0.05257	-675.35437	-675.35312	-674.66455	-674.38756	23.5
**6a_TS4**	95	347	-675.62657	0.31292	-675.29728	0.05547	0.05394	-675.35275	-675.35122	-674.66187	-674.38652	26.2

Table S7. Dihedral angles (***ψ*** and ***ϕ***), absolute electronic energies (E_{el}), zero point energy (ZPE), enthalpy (H), entropies (T.S), quasi-harmonic entropies (T.qh-S), free energies (G), quasi-harmonic Gibbs energy (qh-G), and changes in quasi-harmonic Gibbs free energies (ΔG_{rel}) for stationary points on the FES of **6d**. Geometry and thermochemistry was calculated at the SMD(toluene)-M06-2X/6-31+G* level of theory at 298.15 K and 1 M, and single point corrections were applied from the SMD(toluene)-DLPNO-CCSD(T)/def2-TZVPP level of theory with 'Tight' PNO cut-offs.

			M06-2X/6-31+G*							DLPNO-CCSD(T)/def2-TZVPP		
**Structure**	***ψ*** **/ °**	***ϕ*** **/ °**	**E_{el} / Ha**	**ZPE / Ha**	**H / Ha**	**T.S / Ha**	**T.qh-S / Ha**	**G(T) / Ha**	**qh-G(T) / Ha**	**E_{el} / Ha**	**qh-G(T) / Ha**	**ΔG_{rel} / kJ mol^{-1}**
**6d_A**	183	103	-754.23076	0.37063	-753.84042	0.06432	0.06117	-753.90474	-753.90159	-753.15725	-752.82807	0.0
**6d_A'**	177	257	-754.23076	0.37063	-753.84042	0.06432	0.06117	-753.90474	-753.90159	-753.15725	-752.82807	0.0
**6d_B**	51	39	-754.22503	0.37009	-753.83509	0.06347	0.06081	-753.89856	-753.89590	-753.15268	-752.82355	11.9
**6d_C**	307	135	-754.22531	0.37049	-753.83523	0.06235	0.06017	-753.89758	-753.89540	-753.15268	-752.82277	13.9
**6d_TS1**	257	111	-754.20783	0.37166	-753.81776	0.05990	0.05787	-753.87766	-753.87563	-753.13605	-752.80385	63.6
**6d_TS2**	104	77	-754.20518	0.37128	-753.81554	0.05898	0.05769	-753.87452	-753.87323	-753.13381	-752.80187	68.8
**6d_TS3**	350	87	-754.21927	0.36980	-753.83030	0.06232	0.05976	-753.89262	-753.89005	-753.14767	-752.81845	25.3
**6d_TS4**	97	340	-754.21922	0.37058	-753.82992	0.06030	0.05846	-753.89023	-753.88838	-753.14751	-752.81667	29.9
**6d_TS5**	264	194	-754.22007	0.37055	-753.83080	0.06043	0.05851	-753.89122	-753.88931	-753.14742	-752.81665	30.0

Table S8. Dihedral angles (ψ and ϕ), absolute electronic energies (E_{el}), zero point energy (ZPE), enthalpy (H), entropies (T.S), quasi-harmonic entropies (T.qh-S), free energies (G), quasi-harmonic Gibbs energy (qh-G), and changes in quasi-harmonic Gibbs free energies (ΔG_{rel}) for stationary points on the FES of **6j**. Geometry and thermochemistry was calculated at the SMD(toluene)-M06-2X/6-31+G* level of theory at 298.15 K and 1 M, and single point corrections were applied from the SMD(toluene)-DLPNO-CCSD(T)/def2-TZVPP level of theory with 'Tight' PNO cut-offs.

			**M06-2X/6-31+G***							**DLPNO-CCSD(T)/def2-TZVPP**		
**Structure**	ψ / °	ϕ / °	**E_{el} / Ha**	**ZPE / Ha**	**H / Ha**	**T.S / Ha**	**T.qh-S / Ha**	**G(T) / Ha**	**qh-G(T) / Ha**	**E_{el} / Ha**	**qh-G(T) / Ha**	**ΔG_{rel} / kJ mol^{-1}**
**6j_A**	78	179	-832.81292	0.42751	-832.36307	0.06926	0.06584	-832.43233	-832.42891	-831.63504	-831.25103	0.0
**6j_A'**	282	181	-832.81292	0.42751	-832.36307	0.06926	0.06584	-832.43233	-832.42891	-831.63504	-831.25103	0.0
**6j_TS1**	240	116	-832.78437	0.42957	-832.33424	0.06318	0.06147	-832.39742	-832.39571	-831.60737	-831.21872	84.8
**6j_TS2**	63	117	-832.78228	0.42953	-832.33215	0.06276	0.06161	-832.39490	-832.39375	-831.60553	-831.21701	89.3
**6j_TS3**	100	6	-832.78467	0.42740	-832.33607	0.06435	0.06276	-832.40042	-832.39883	-831.60733	-831.22149	77.6

Table S9. Dihedral angles (ψ and ϕ), absolute electronic energies (E_{el}), zero point energy (ZPE), enthalpy (H), entropies (T.S), quasi-harmonic entropies (T.qh-S), free energies (G), quasi-harmonic Gibbs energy (qh-G), and changes in quasi-harmonic Gibbs free energies (ΔG_{rel}) for stationary points on the FES of **6t**. Geometry and thermochemistry was calculated at the SMD(toluene)-M06-2X/6-31+G* level of theory at 298.15 K and 1 M, and single point corrections were applied from the SMD(toluene)-DLPNO-CCSD(T)/def2-TZVPP level of theory with 'Tight' PNO cut-offs.

			**M06-2X/6-31+G***							**DLPNO-CCSD(T)/def2-TZVPP**		
**Structure**	ψ / °	ϕ / °	**E_{el} / Ha**	**ZPE / Ha**	**H / Ha**	**T.S / Ha**	**T.qh-S / Ha**	**G(T) / Ha**	**qh-G(T) / Ha**	**E_{el} / Ha**	**qh-G(T) / Ha**	**ΔG_{rel} / kJ mol^{-1}**
**6t_A**	132	144	-1064.99111	0.53048	-1064.43297	0.08004	0.07577	-1064.51301	-1064.50874	-1063.47281	-1062.99043	0.0
**6t_A'**	228	216	-1064.99111	0.53048	-1064.43297	0.08004	0.07577	-1064.51301	-1064.50874	-1063.47281	-1062.99043	0.0
**6t_TS1**	93	86	-1064.94187	0.53028	-1064.38557	0.07509	0.07230	-1064.46066	-1064.45787	-1063.42646	-1062.94246	126.0
**6t_TS2**	110	258	-1064.94086	0.53222	-1064.38265	0.07477	0.07206	-1064.45743	-1064.45471	-1063.42152	-1062.93538	144.5
**6t_TS3**	358	96	-1064.96804	0.53060	-1064.41023	0.07854	0.07490	-1064.48877	-1064.48513	-1063.45120	-1062.96830	58.1

Bibliography

1 R. B. King, J. C. Cloyd and R. H. Reimann, *J. Org. Chem.*, 1976, **41**, 972–977.

2 Y. Hou and C. Y. Meyers, *J. Org. Chem.*, 2004, **69**, 1186–1195.

3 J. Clayden, J. Senior and M. Helliwell, *Angew. Chem. Int. Ed.*, 2009, **48**, 6270–6273.

4 J. Burgers, M. A. Hoefnagel, P. E. Verkade, H. Visser and B. M. Wepster, *Receuil des Trav. Chim. des Pays-Bas*, 1958, **77**, 491–530.

5 M. Arisawa, M. Kuwajima and M. Yamaguchi, *Tetrahedron Lett.*, 2010, **51**, 3116–3118.

6 C. M. D. Komen and F. Bickelhaupt, *Synth. Commun.*, 1996, **26**, 1693–1697.

7 O. Trapp, *Anal. Chem.*, 2006, **78**, 189–198.

8 M. S. Betson, J. Clayden, C. P. Worrall and S. Peace, *Angew. Chem. Int. Ed.*, 2006, **45**, 5803–5807.

9 S. Pronk, S. Páll, R. Schulz, P. Larsson, P. Bjelkmar, R. Apostolov, M. R. Shirts, J. C. Smith, P. M. Kasson, D. Van Der Spoel, B. Hess and E. Lindahl, *Bioinformatics*, 2013, **29**, 845–854.

10 M. J. Abraham, D. van der Spoel, E. Lindhal and B. Hess, and the GROMACS development team, GROMACS User Manual version 5.1.4, www.gromacs.org, 2017 (accessed 7 March 2018).

11 G. A. Tribello, M. Bonomi, D. Branduardi, C. Camilloni and G. Bussi, *Comput. Phys. Commun.*, 2014, **185**, 604–613.

12 *Schrödinger Release 2013-3: MacroModel version 10.2, Schrödinger LLC, New York, NY, 2013*, .

13 C. Caleman, P. J. Van Maaren, M. Hong, J. S. Hub, L. T. Costa and D. van der Spoel, *J. Chem. Theory Comput.*, 2012, **8**, 61–74.

14 T. Darden, D. York and L. Pedersen, *J. Chem. Phys.*, 1993, **98**, 10089.

15 G. Bussi, D. Donadio and M. Parrinello, *J. Chem. Phys.*, 2007, **126**, 014101.

16 M. Parrinello and A. Rahman, *J. Appl. Phys.*, 1981, **52**, 7182.

17 A. Laio and M. Parrinello, *Proc. Natl. Acad. Sci.*, 2002, **99**, 12562–12566.

18 Y. Zhao and D. G. Truhlar, *Theor. Chem. Acc.*, 2008, **120**, 215–241.

19 A. V. Marenich, C. J. Cramer and D. G. Truhlar, *J. Phys. Chem. B*, 2009, **113**, 6378–6396.

20 I. Funes-Ardois and R. S. Paton, http://patonlab.com/code/ (accessed 09 May 2020).

21 S. Grimme, *Chem. A Eur. J.*, 2012, **18**, 9955–9964.

22 M. Charton, *J. Am. Chem. Soc.*, 1975, **97**, 1552–1556.

23 M. Charton, *J. Org. Chem.*, 1976, **41**, 2217–2220.

24 A. F. Zahrt, S. V. Athavale and S. E. Denmark, *Chem. Rev.*, 2020, **120**, 1620–1689.

^{1}H and ^{13}C NMR spectra of new compounds

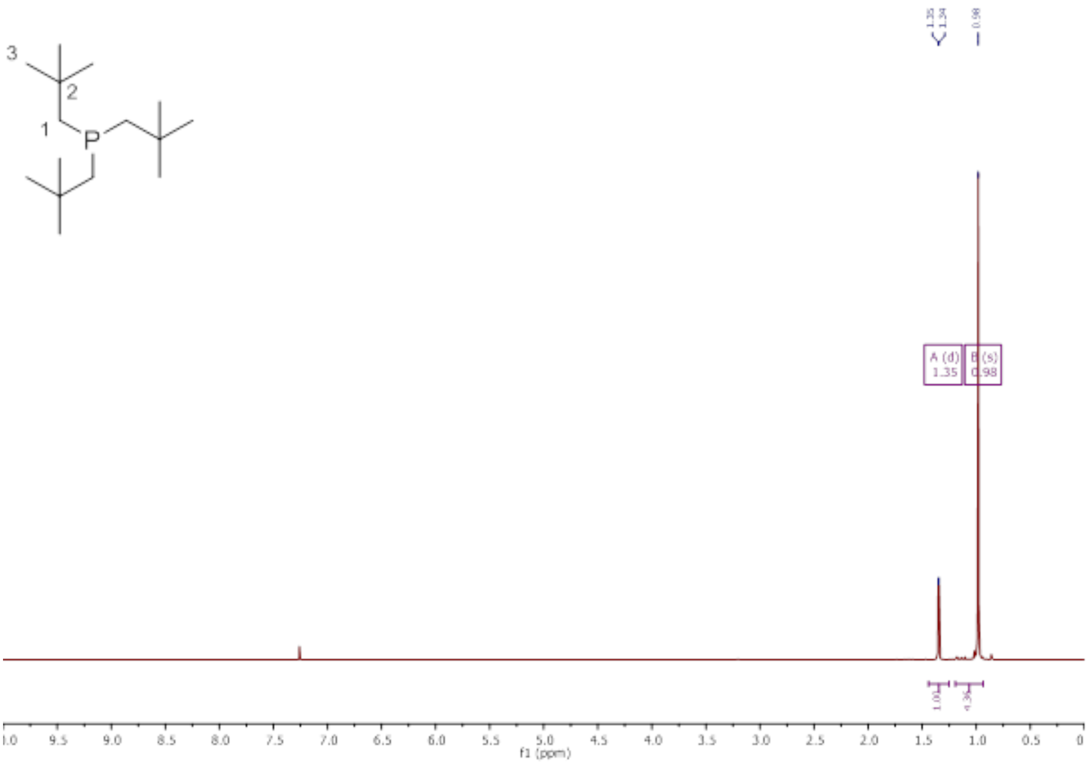

^{1}H NMR spectrum of PNp_3

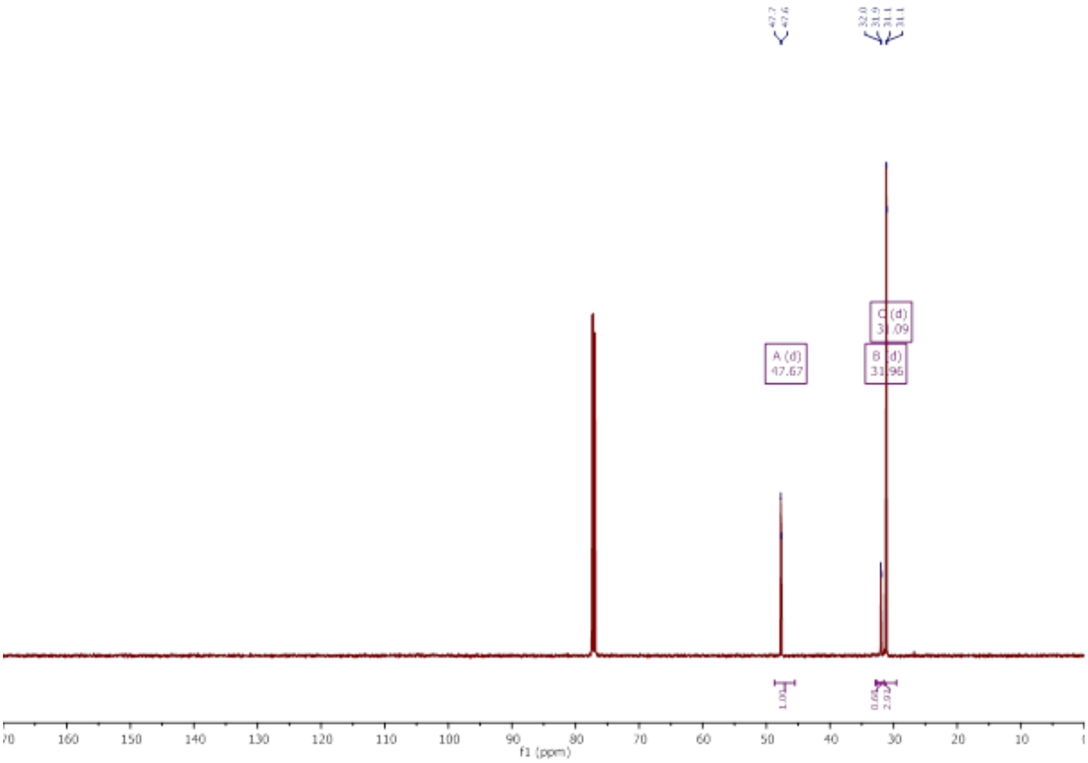

^{13}C NMR spectrum of PNp_3

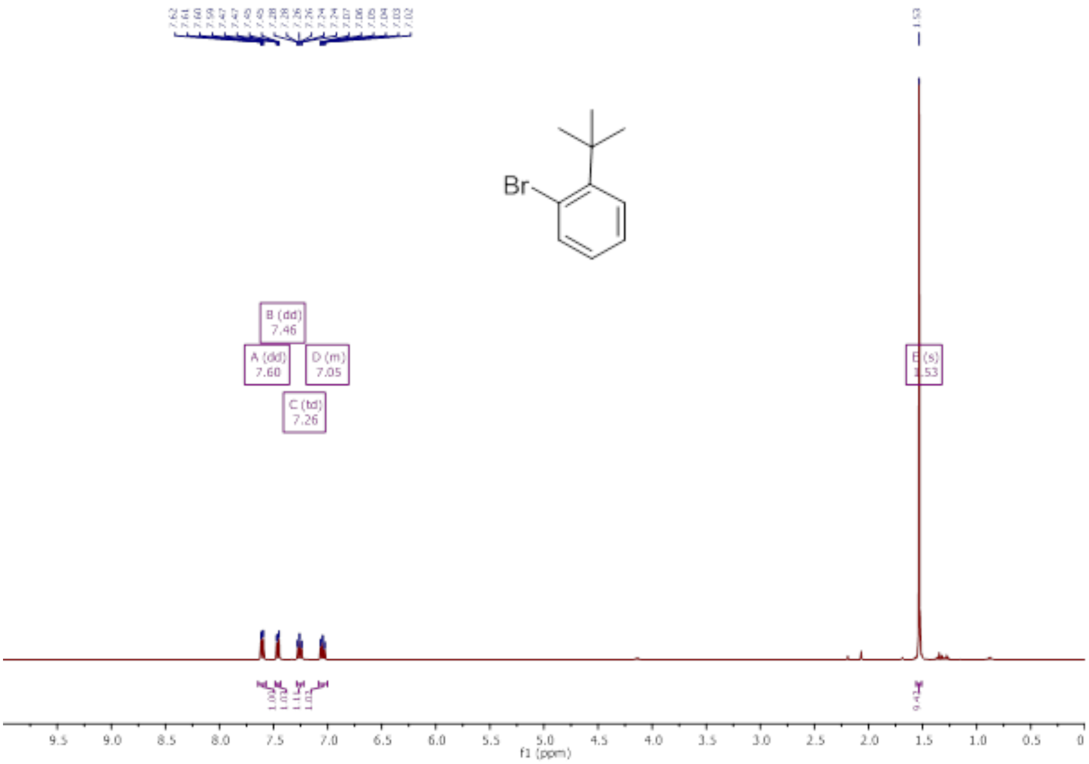

^{1}H NMR spectrum of 1-bromo-2-(*tert*-butyl)benzene

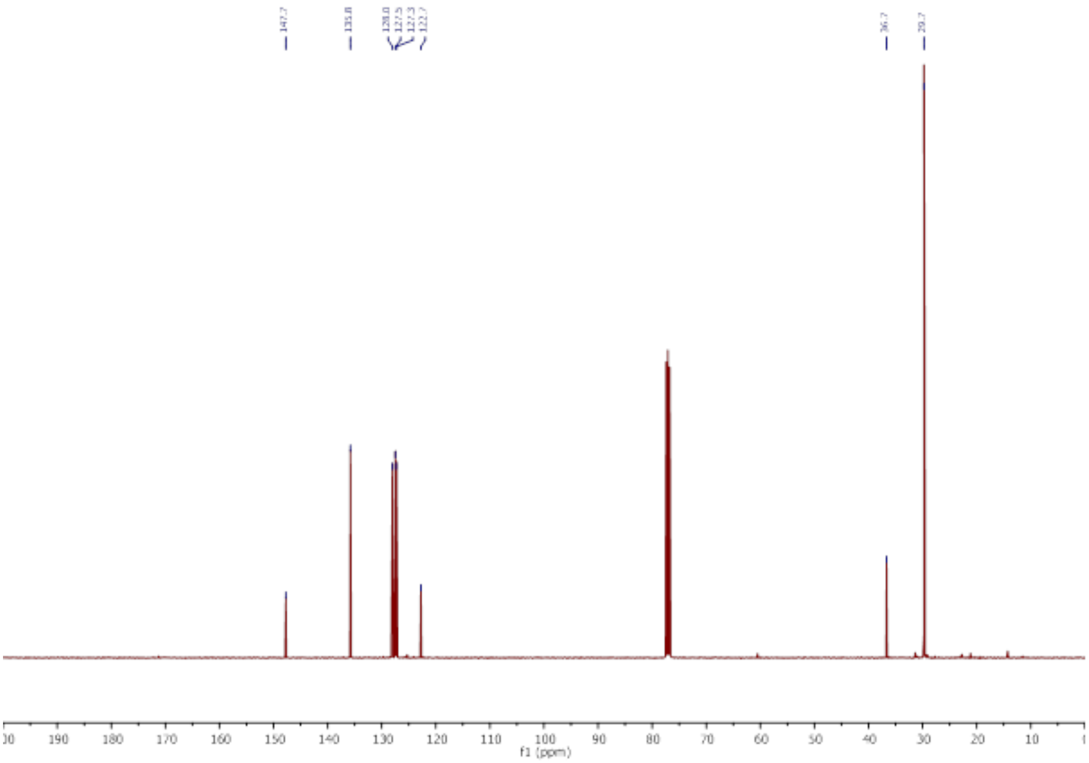

^{13}C NMR spectrum of 1-bromo-2-(*tert*-butyl)benzene

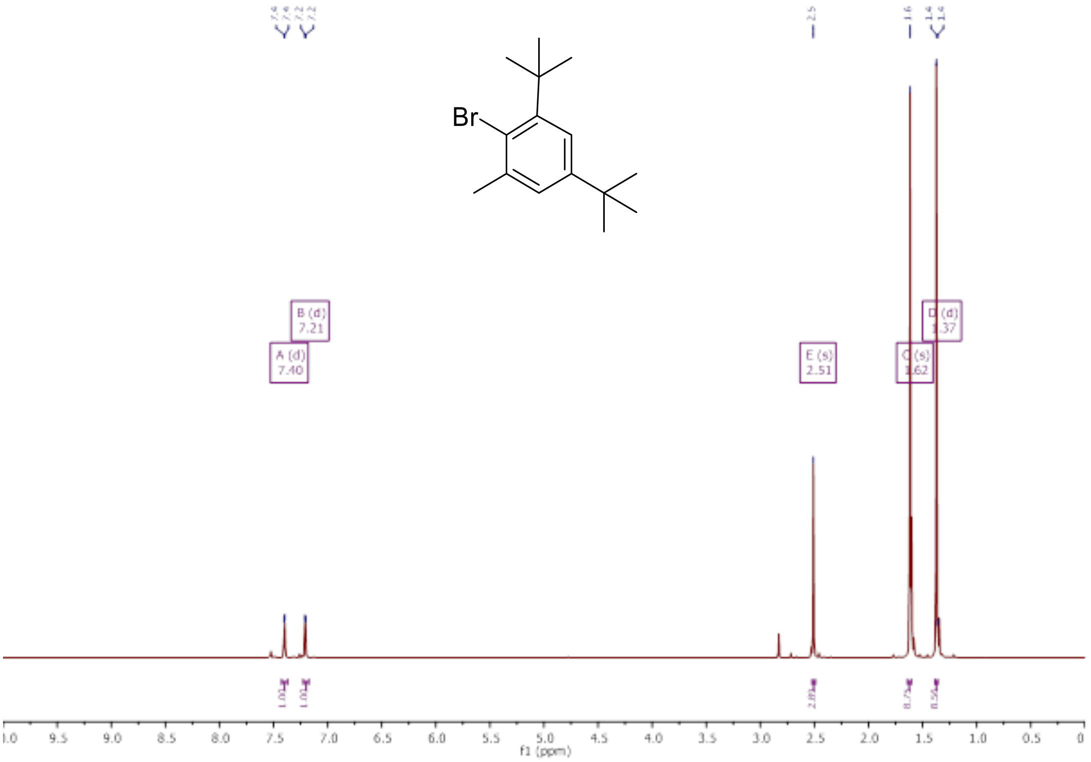

^{1}H NMR spectrum of 2-bromo-1,5-di-*tert*-butyl-3-methylbenzene

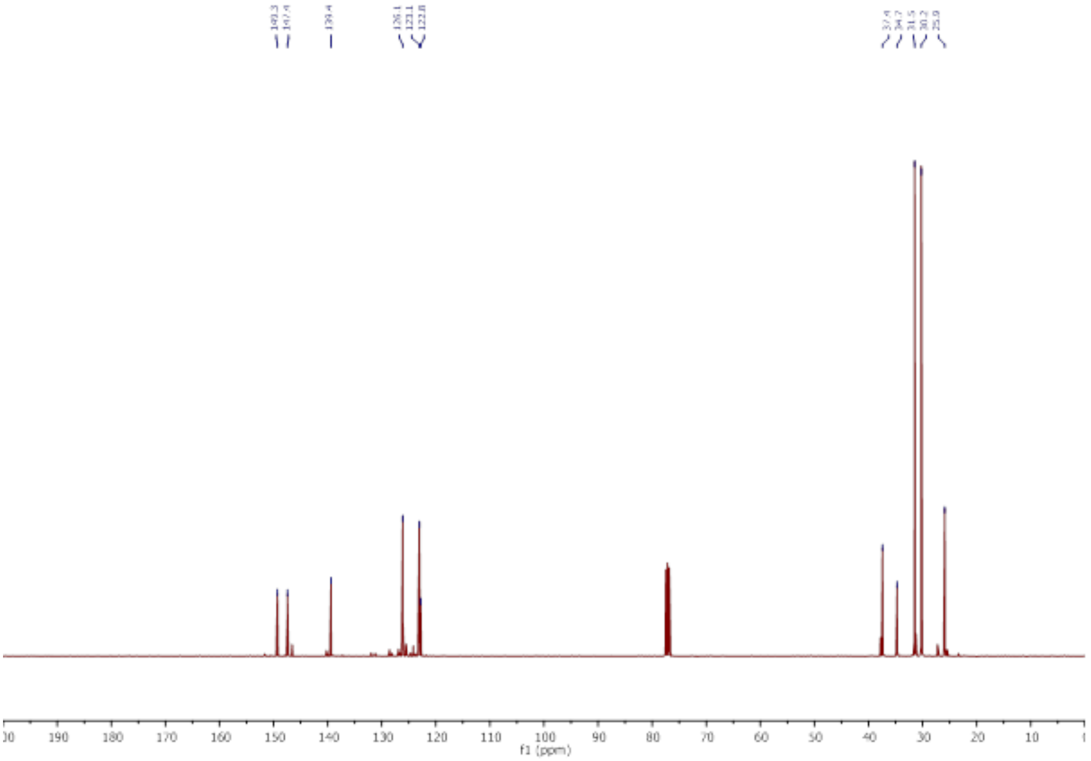

^{13}C NMR spectrum of 2-bromo-1,5-di-*tert*-butyl-3-methylbenzene

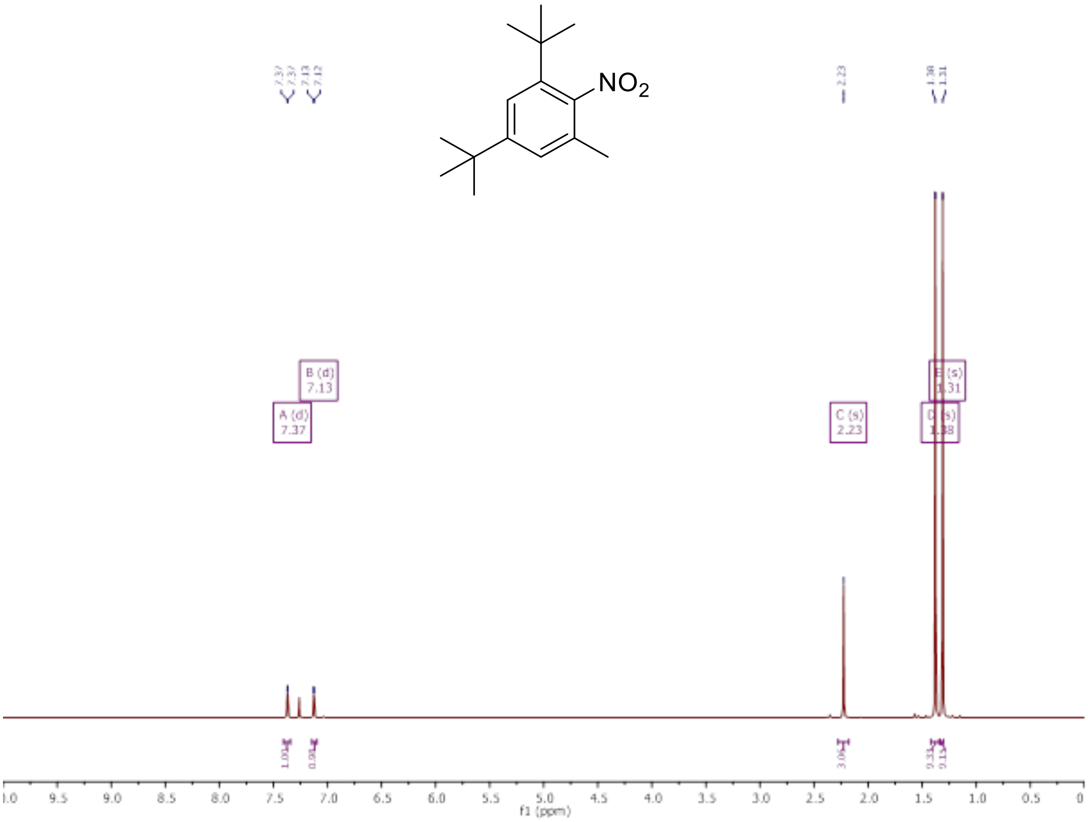

^{1}H NMR spectrum of 1,5-di-*tert*-butyl-3-methyl-2-nitrobenzene

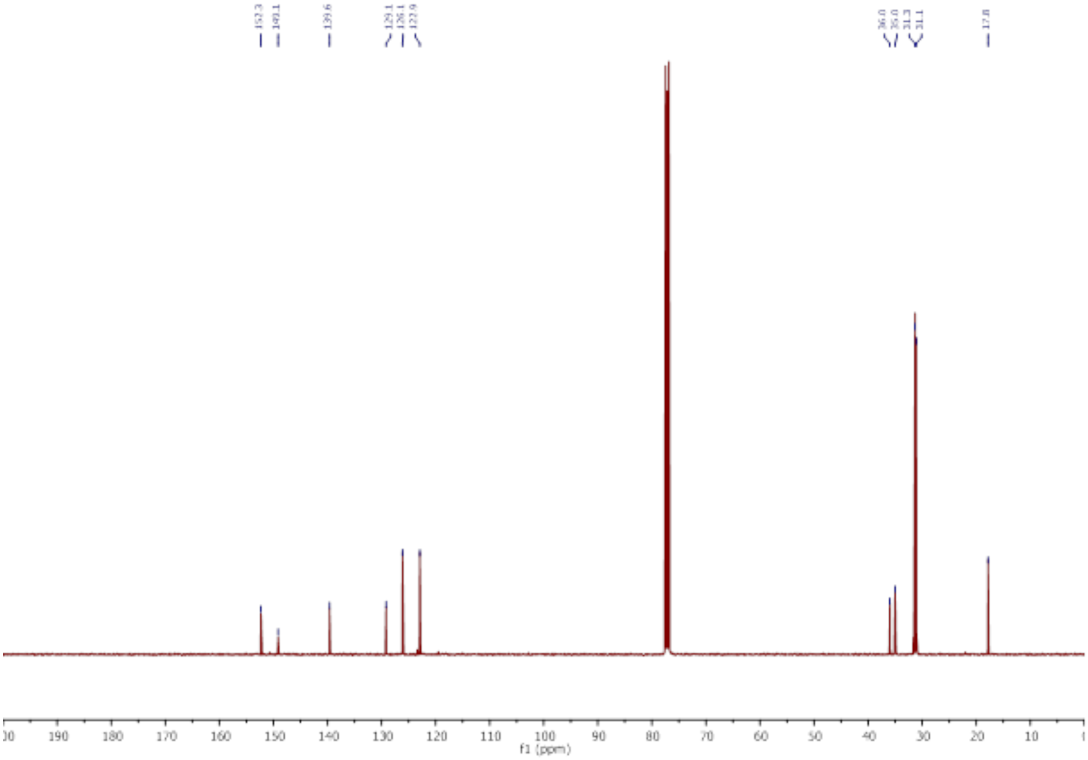

^{13}C NMR spectrum of 1,5-di-*tert*-butyl-3-methyl-2-nitrobenzene

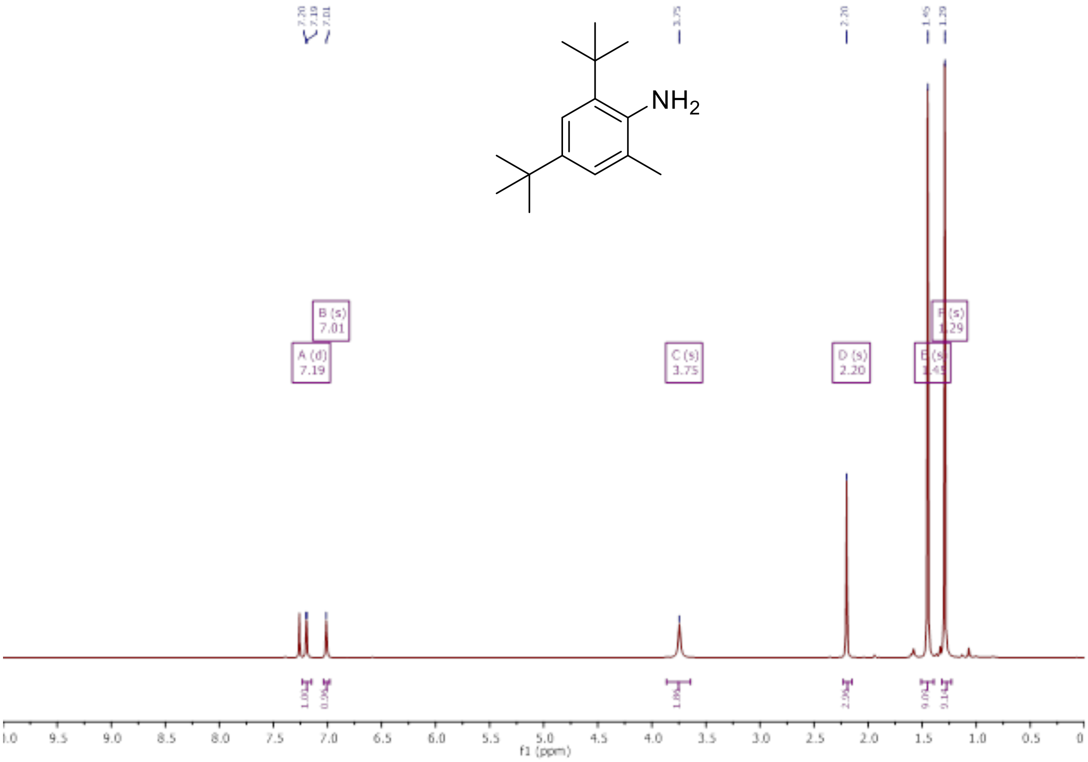

^{1}H NMR spectrum of 2,4-di-*tert*-butyl-6-methylaniline

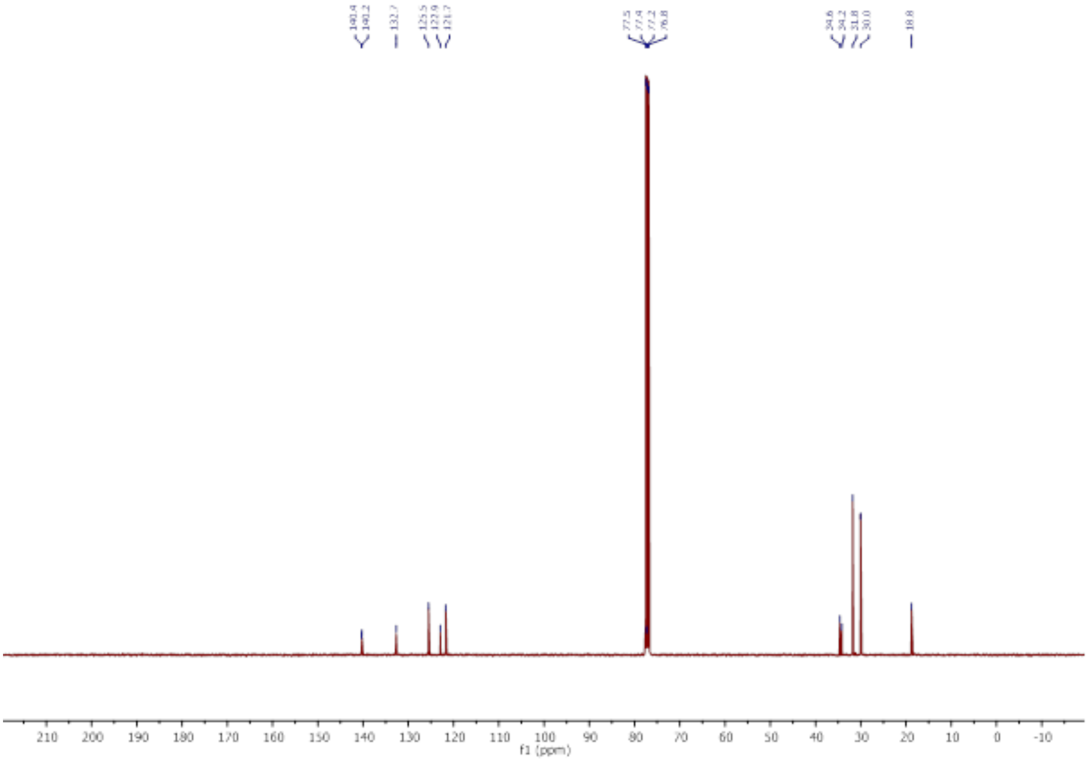

^{13}C NMR spectrum of 2,4-di-*tert*-butyl-6-methylaniline

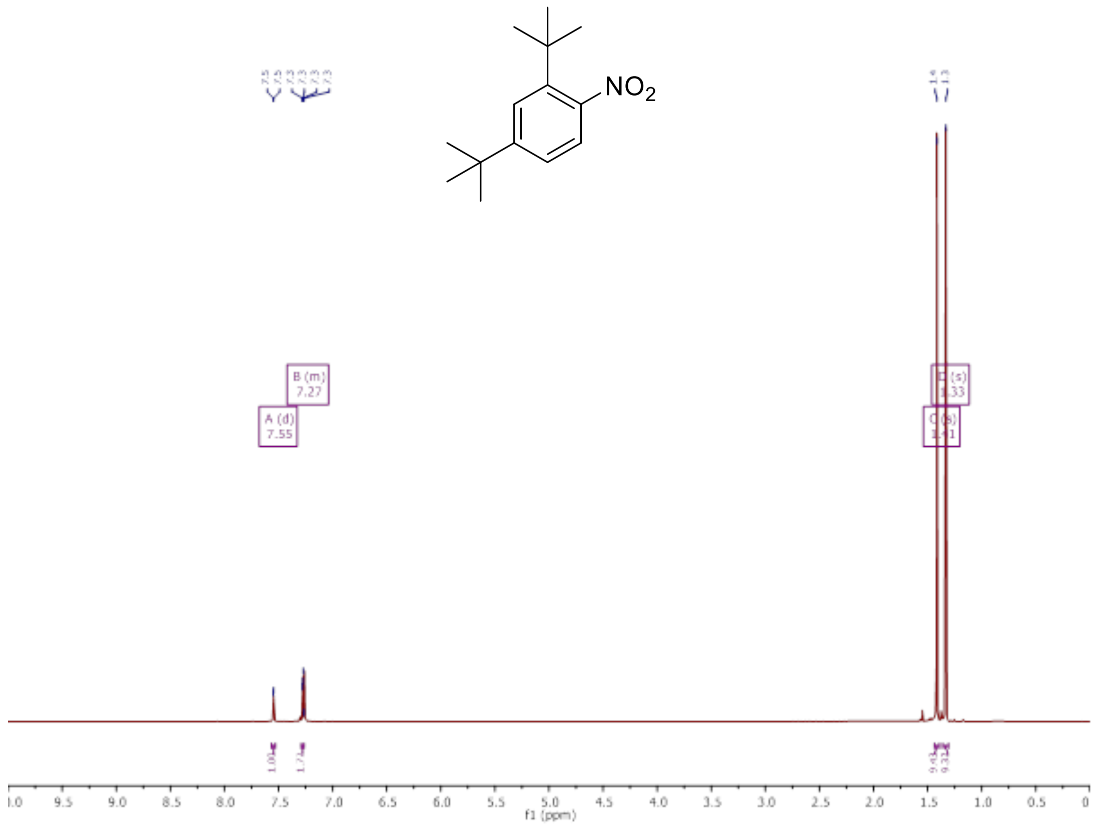

^{1}H NMR spectrum of 2,4-di-*tert*-butyl-1-nitrobenzene

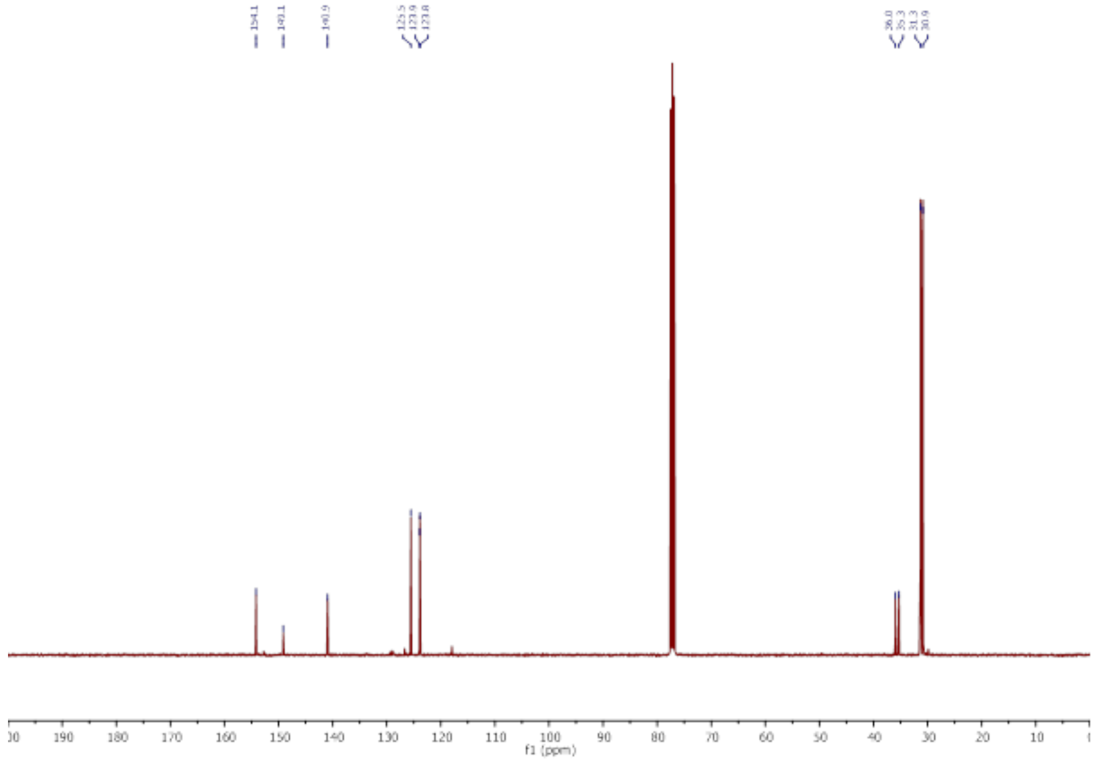

^{13}C NMR spectrum of 2,4-di-*tert*-butyl-1-nitrobenzene

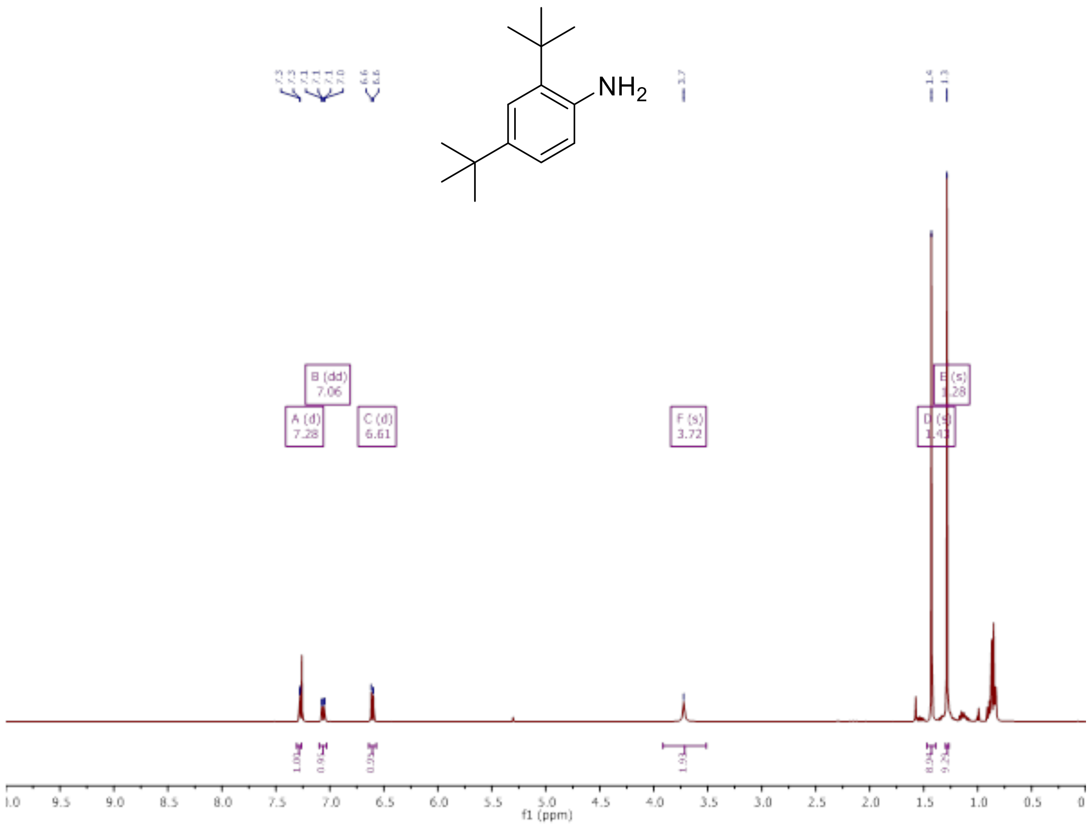

^{1}H NMR spectrum of 2,4-di-*tert*-butyl-aniline

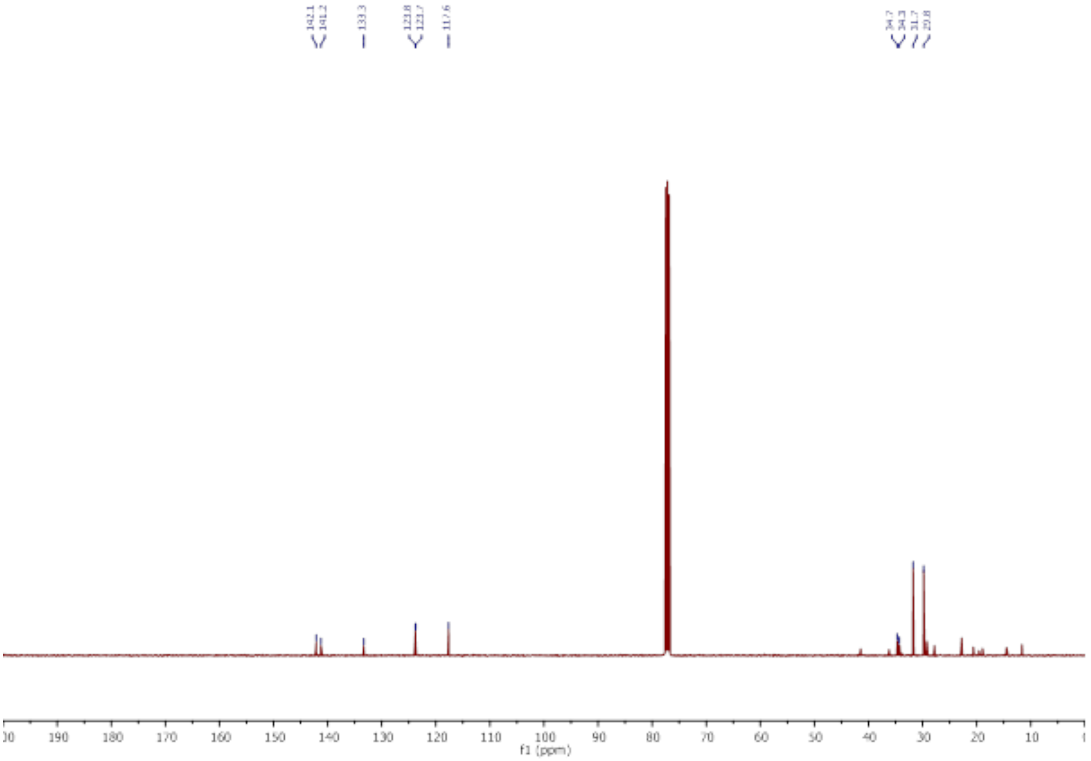

^{13}C NMR spectrum of 2,4-di-*tert*-butyl-aniline

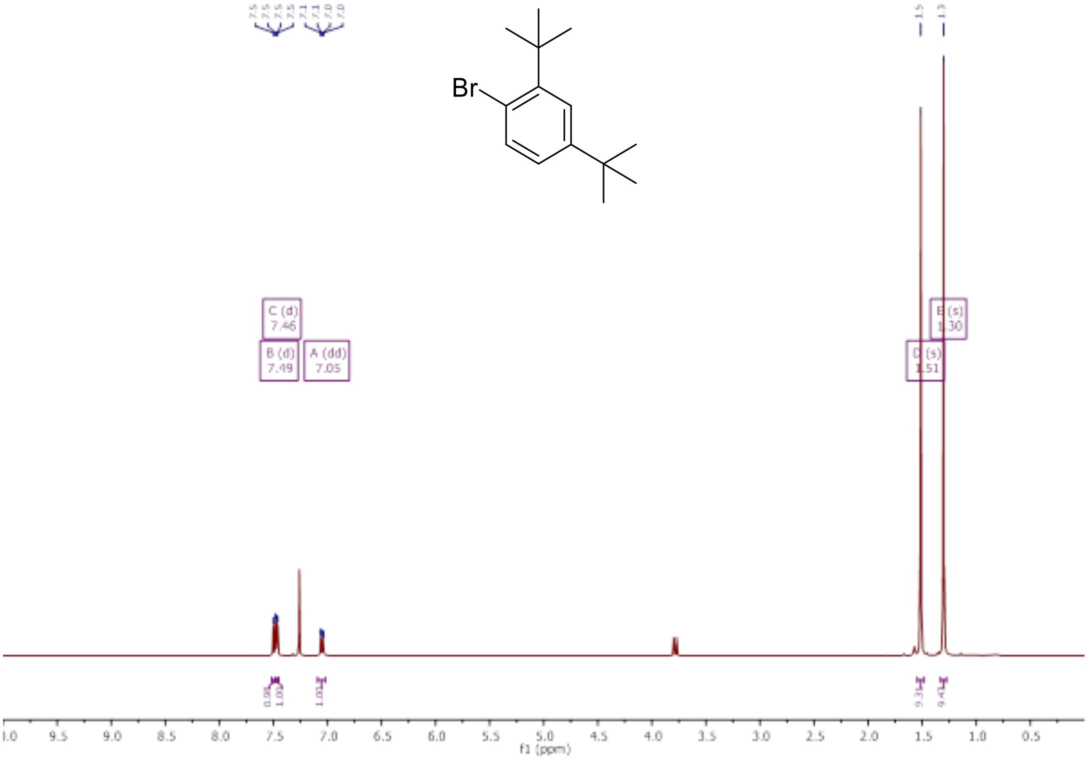

^{1}H NMR spectrum of 2,4-di-*tert*-butyl-1-bromobenzene

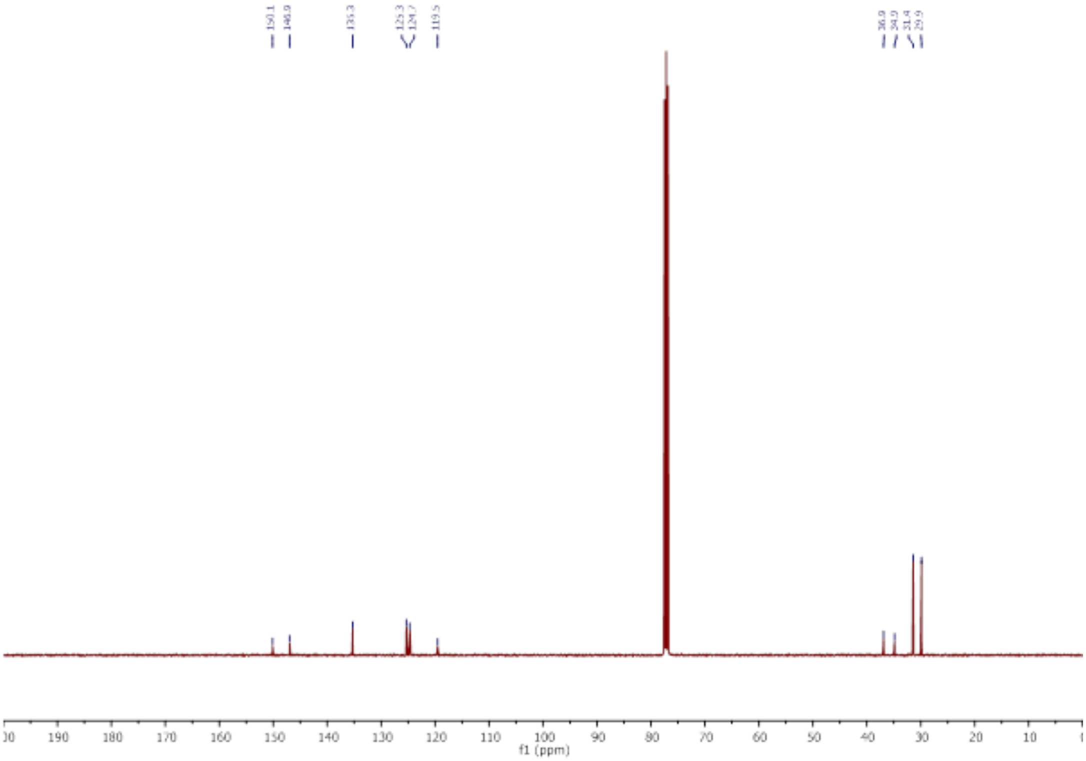

^{13}C NMR spectrum of 2,4-di-*tert*-butyl-1-bromobenzene

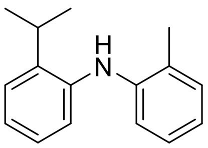

6a

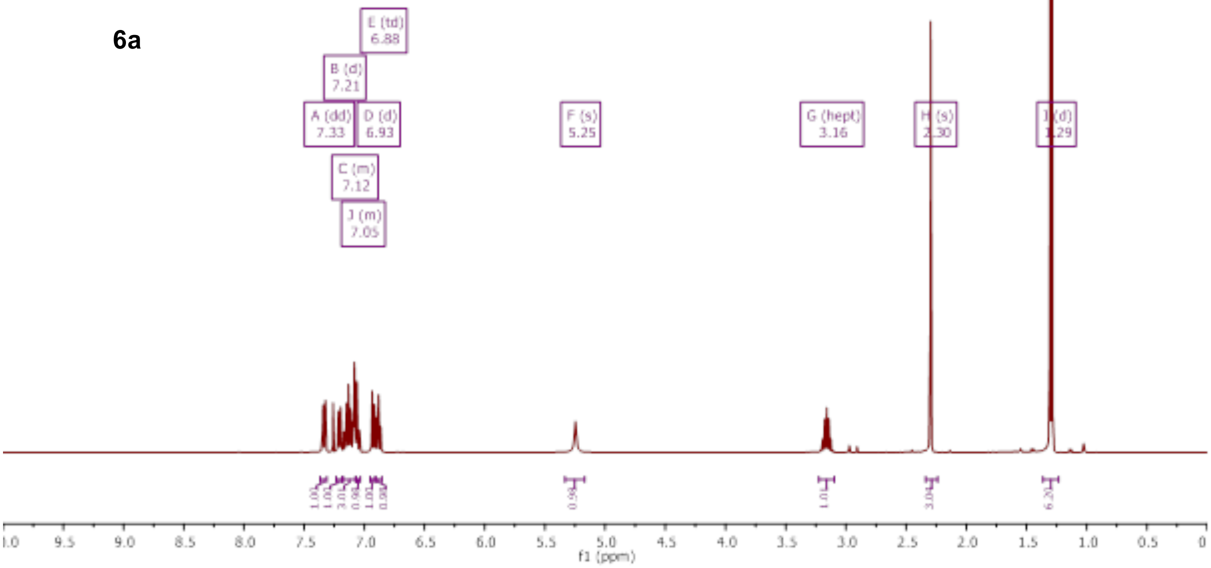

^{1}H NMR spectrum of **6a**

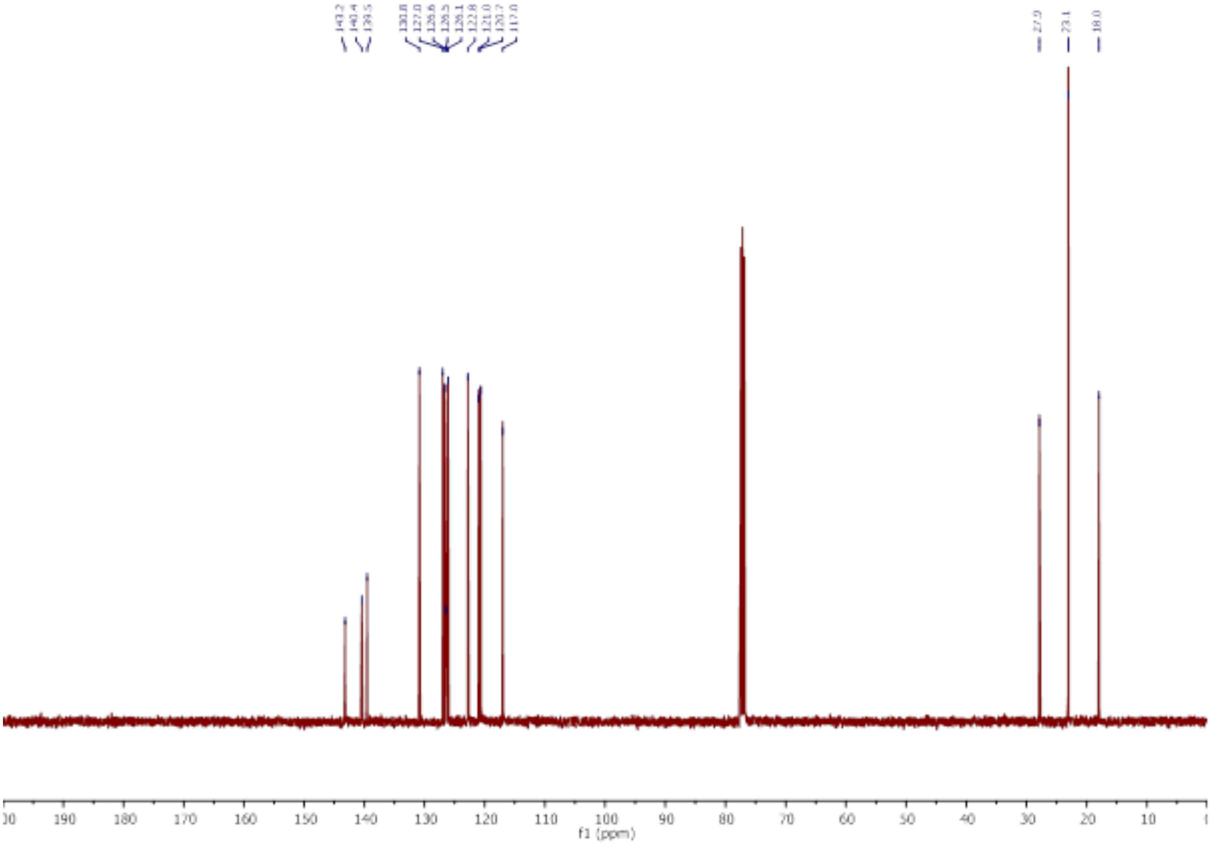

^{13}C NMR spectrum of **6a**

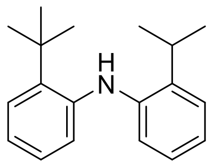

6b

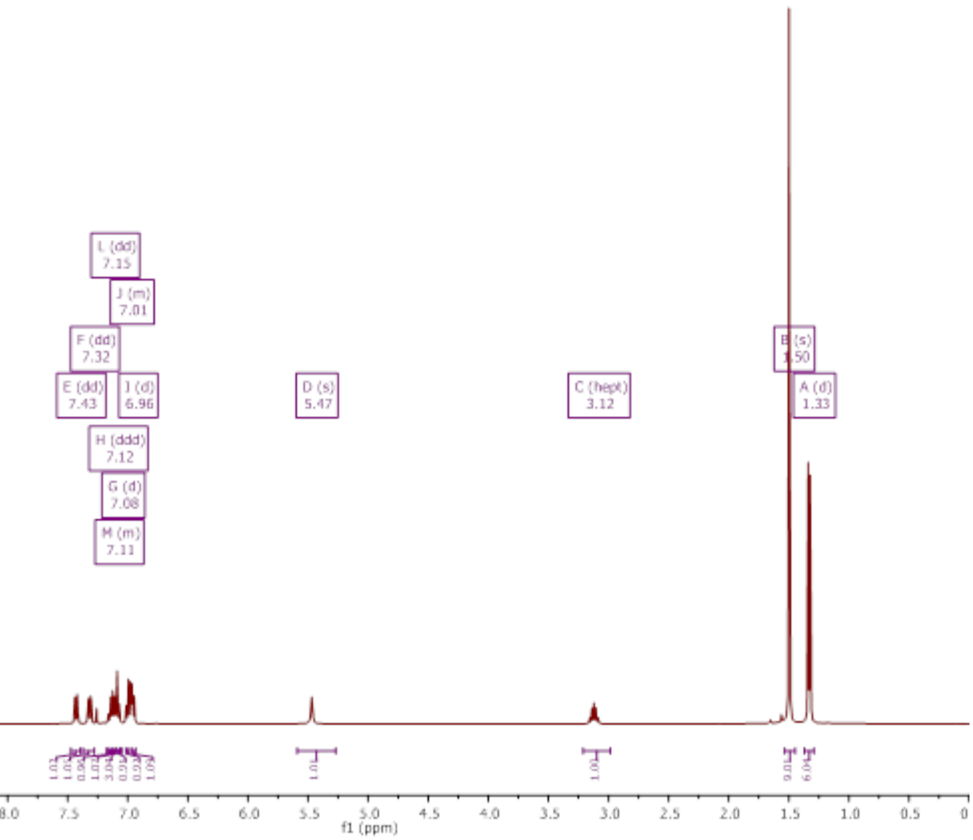

^{1}H NMR spectrum of **6b**

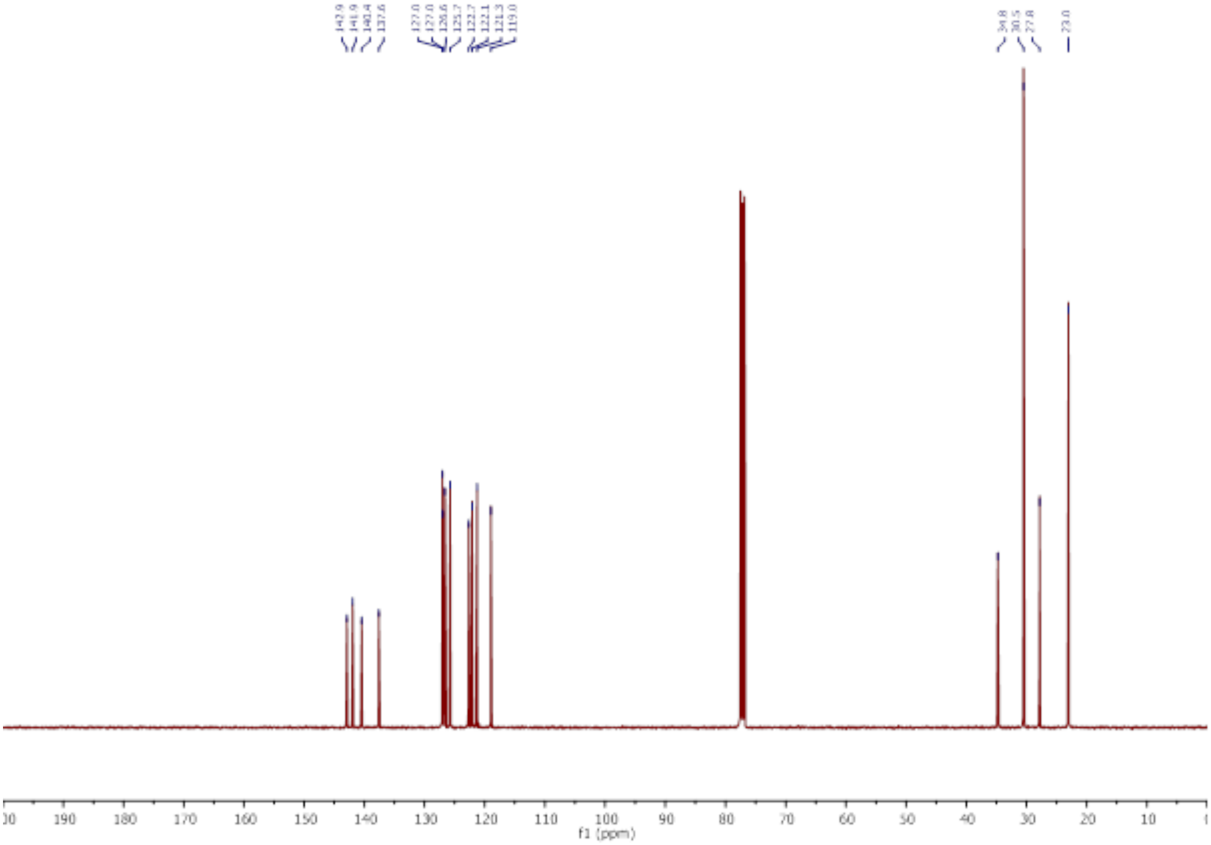

^{13}C NMR spectrum of **6b**

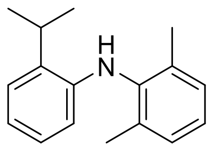

6c

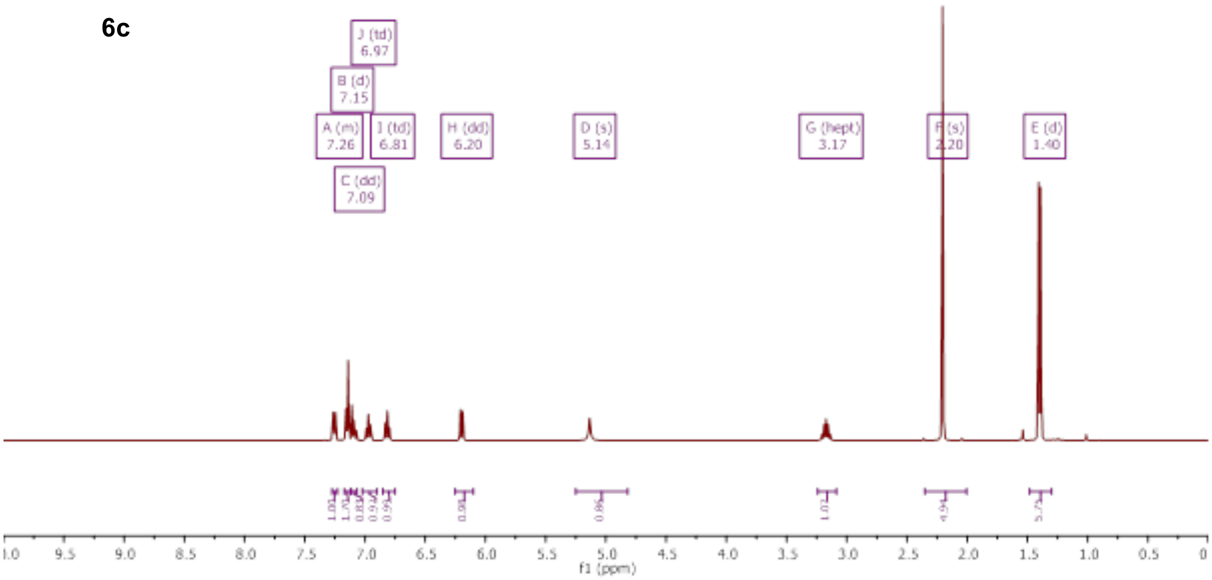

^{1}H NMR spectrum of **6c**

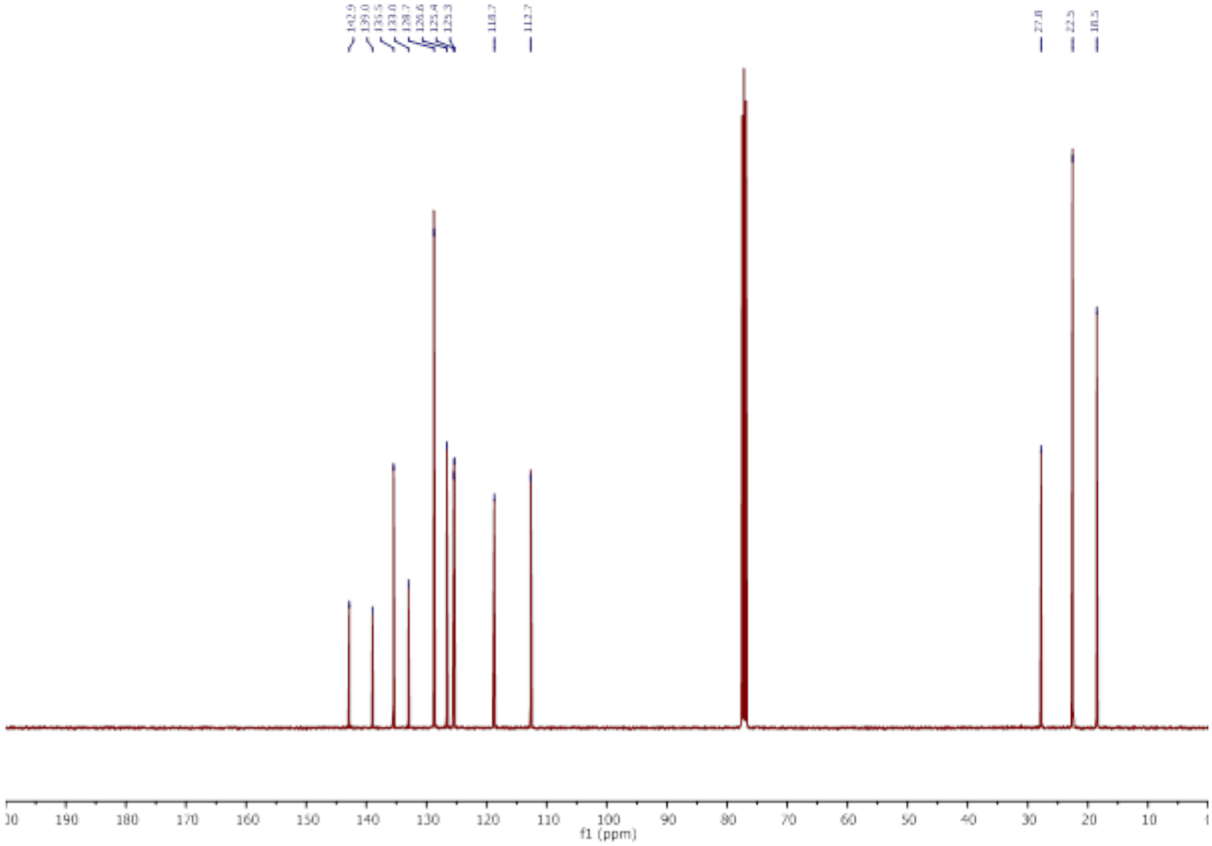

^{13}C NMR spectrum of **6c**

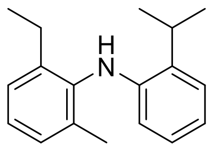

6d

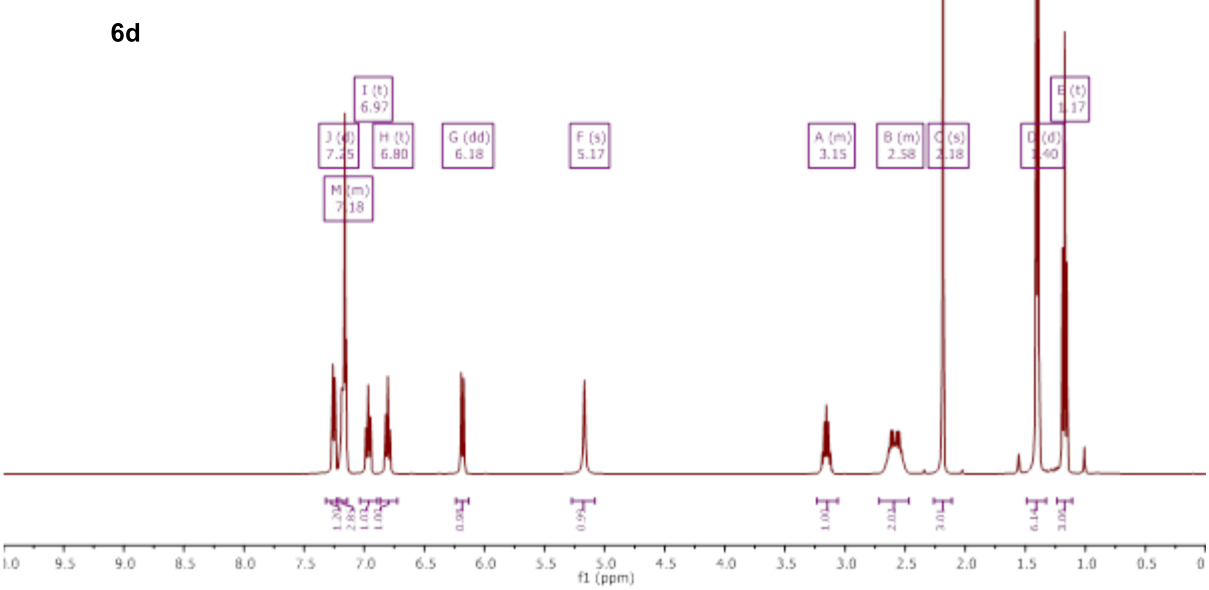

^{1}H NMR spectrum of **6d**

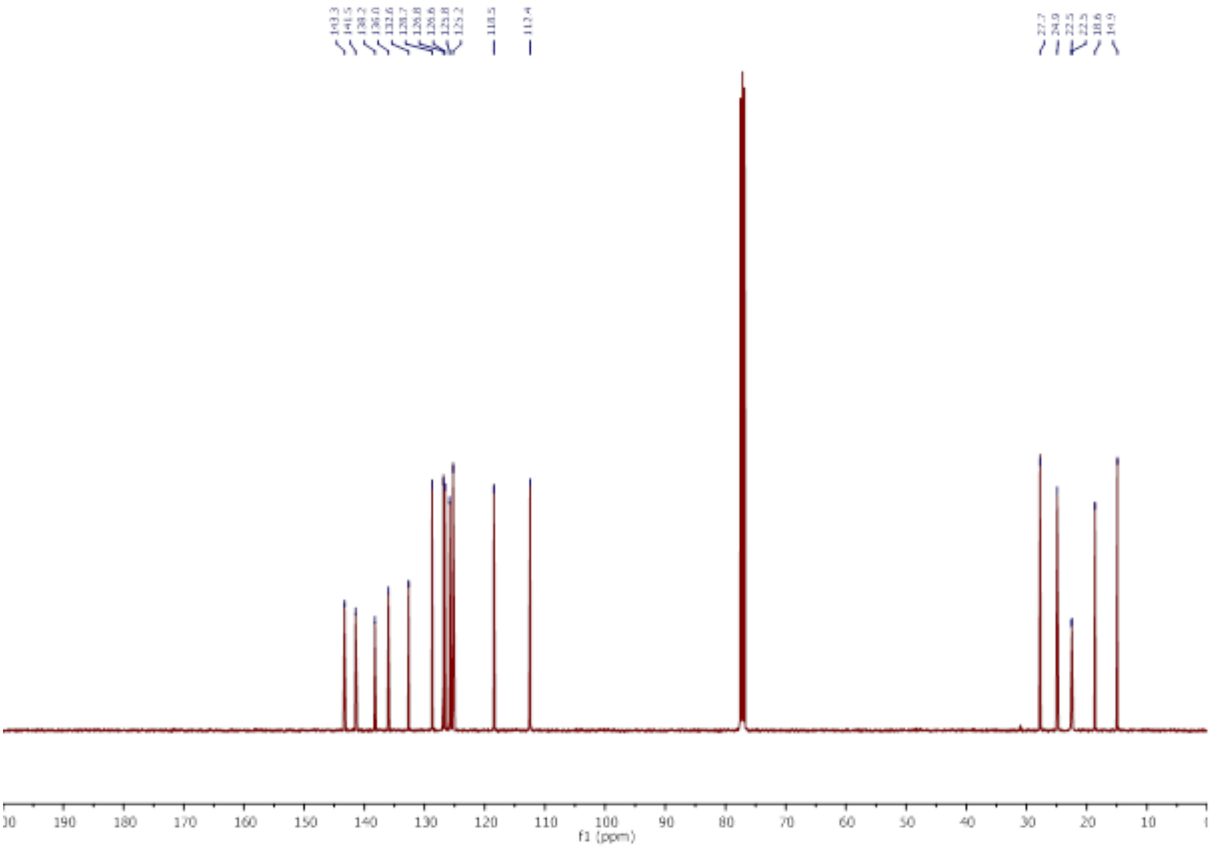

^{13}C NMR spectrum of **6d**

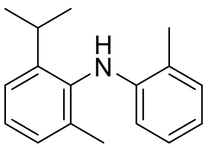

6e

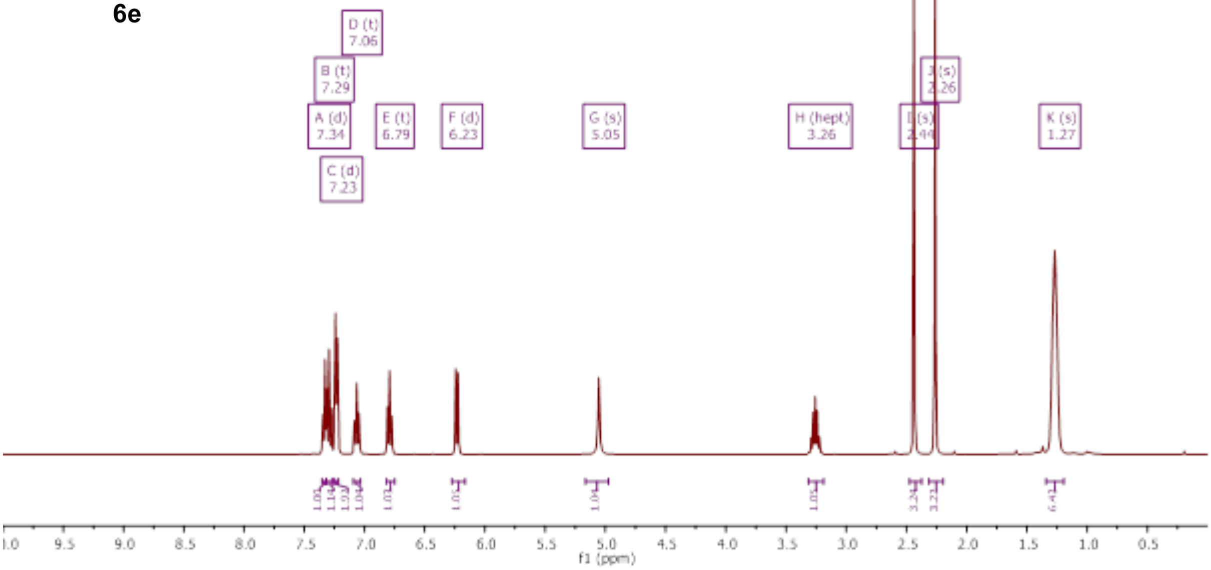

^{1}H NMR spectrum of **6e**

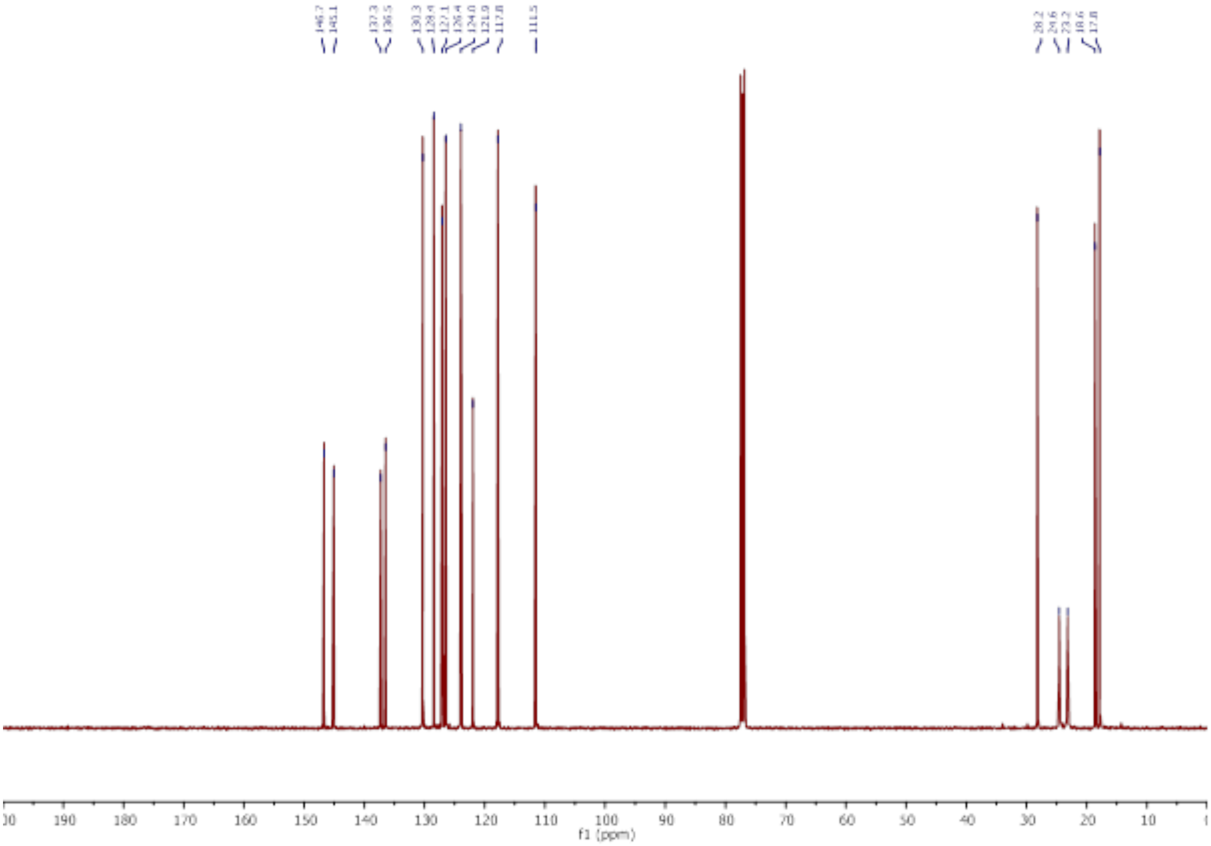

^{13}C NMR spectrum of **6e**

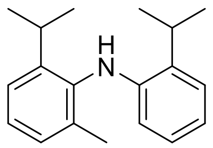

6f

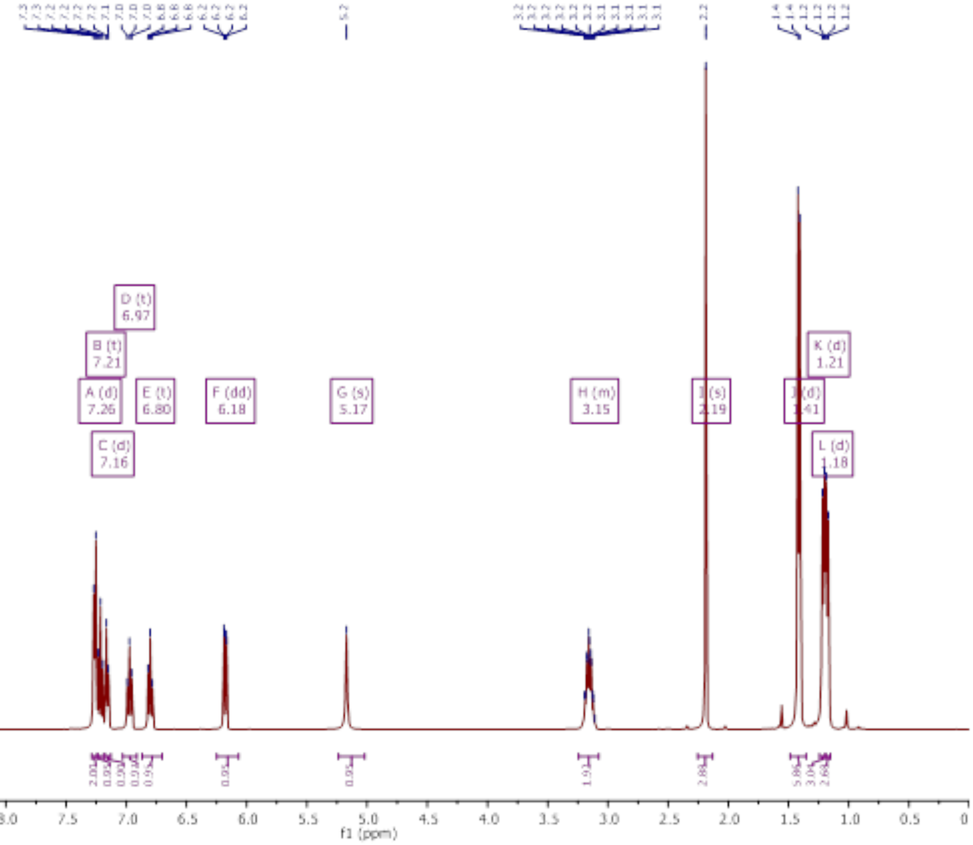

^{1}H NMR spectrum of **6f**

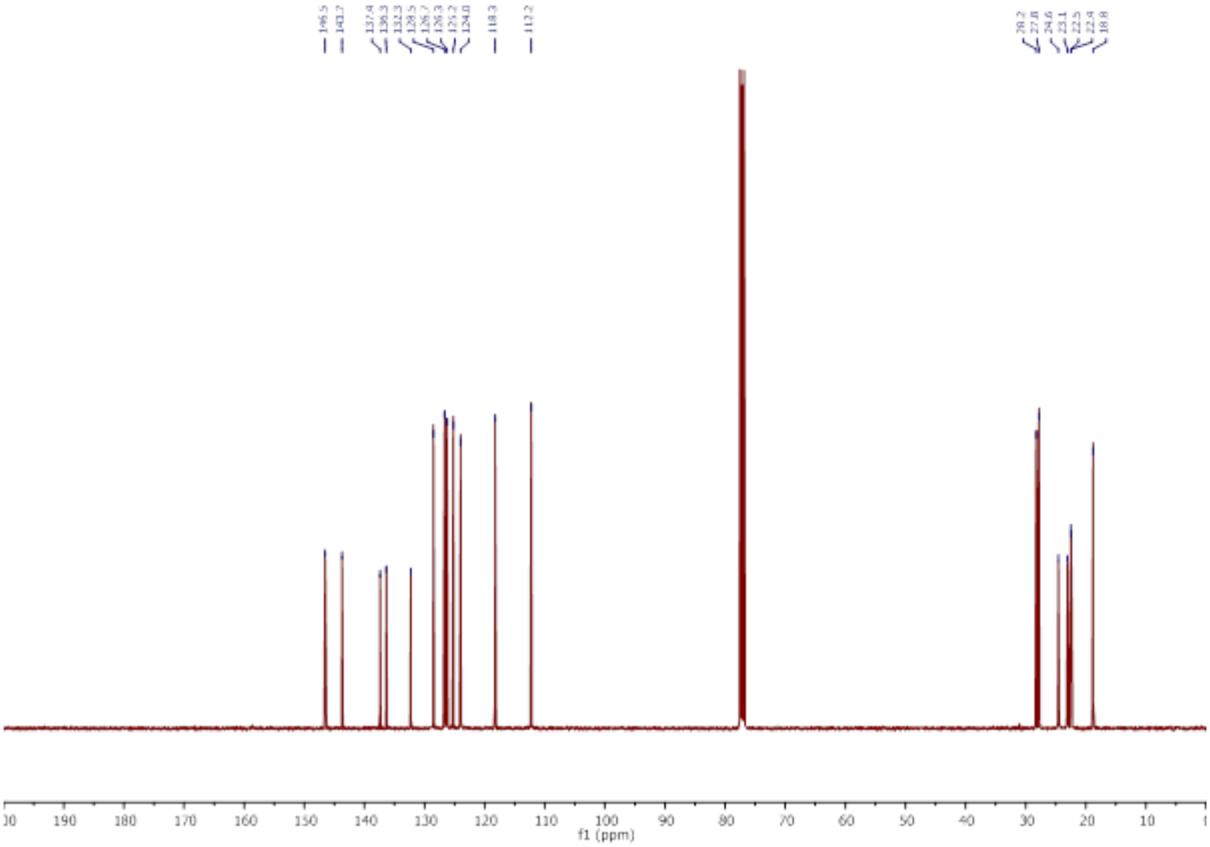

^{13}C NMR spectrum of **6f**

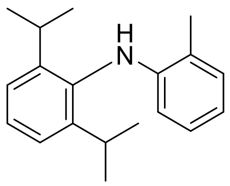

6g

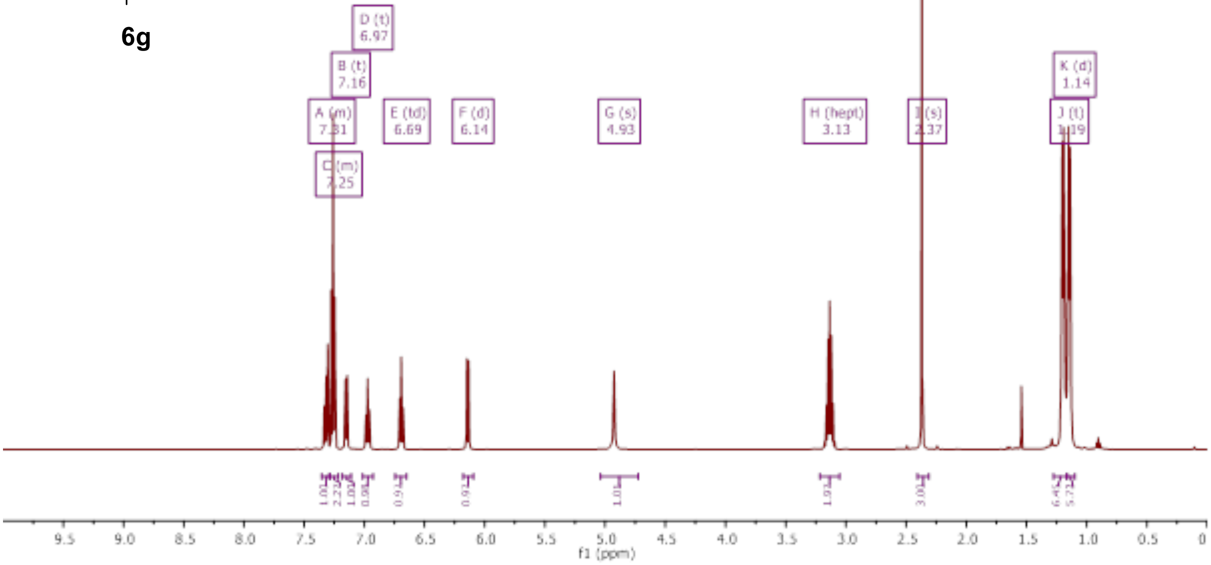

^{1}H NMR spectrum of **6g**

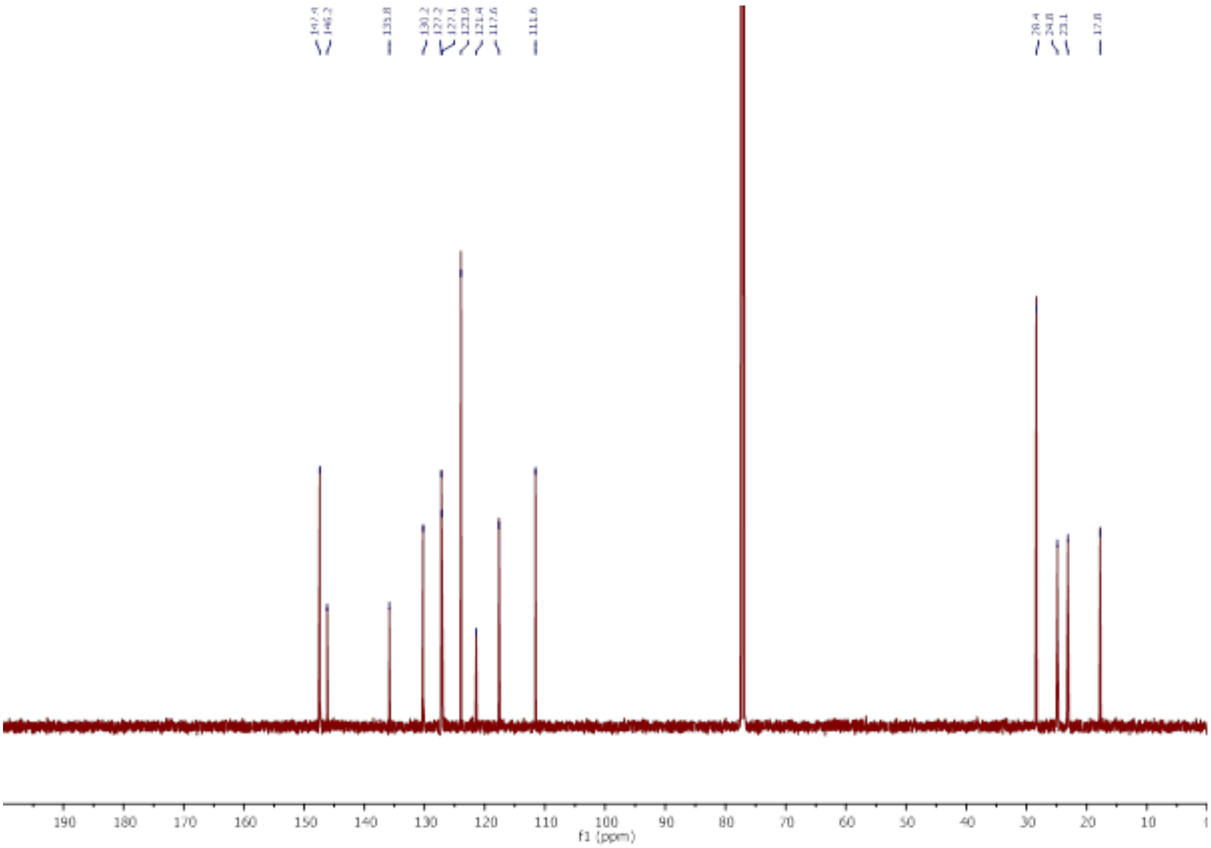

^{13}C NMR spectrum of **6g**

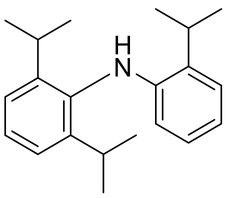

6h

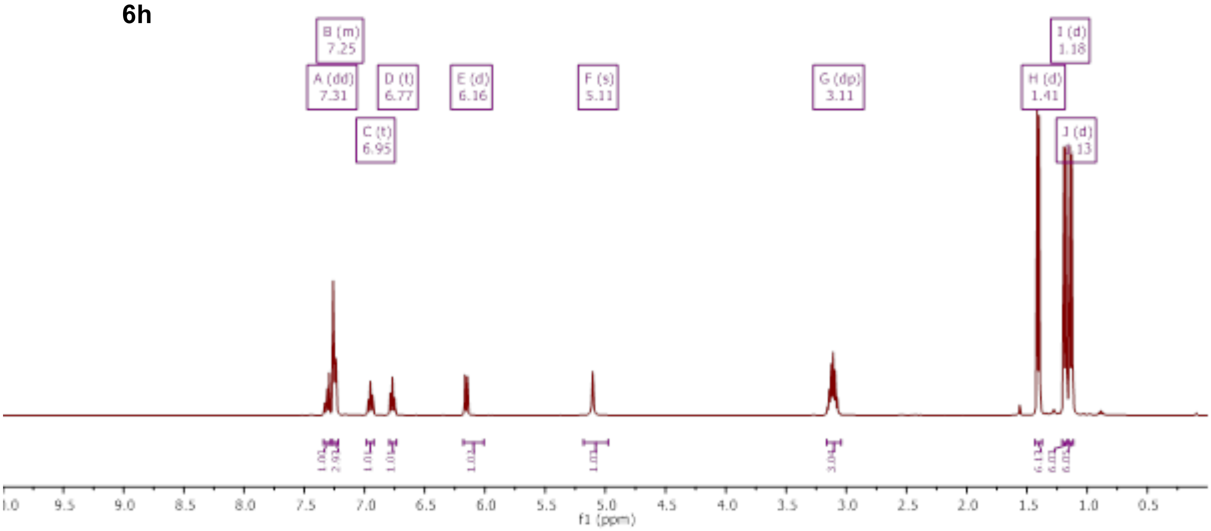

^{1}H NMR spectrum of **6h**

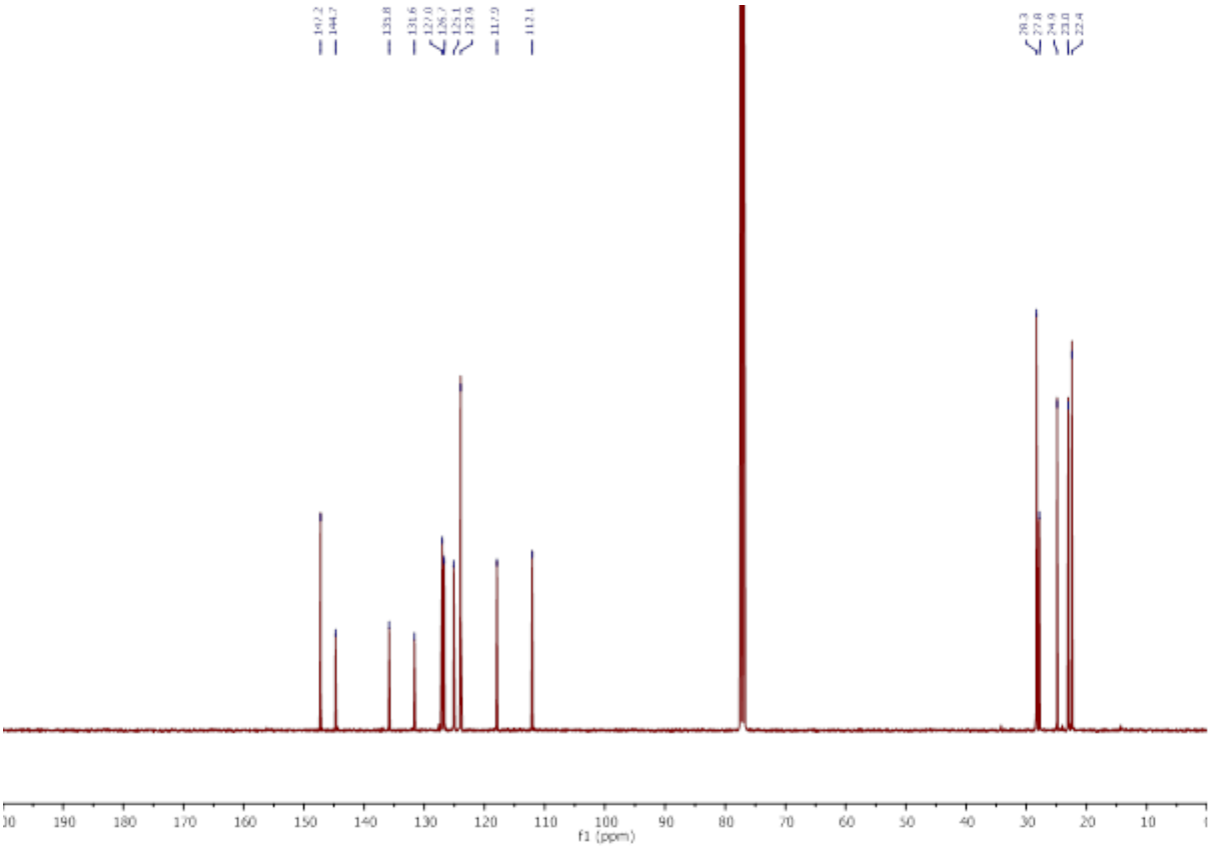

^{13}C NMR spectrum of **6h**

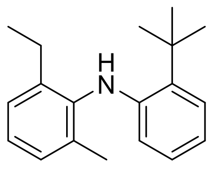

6i

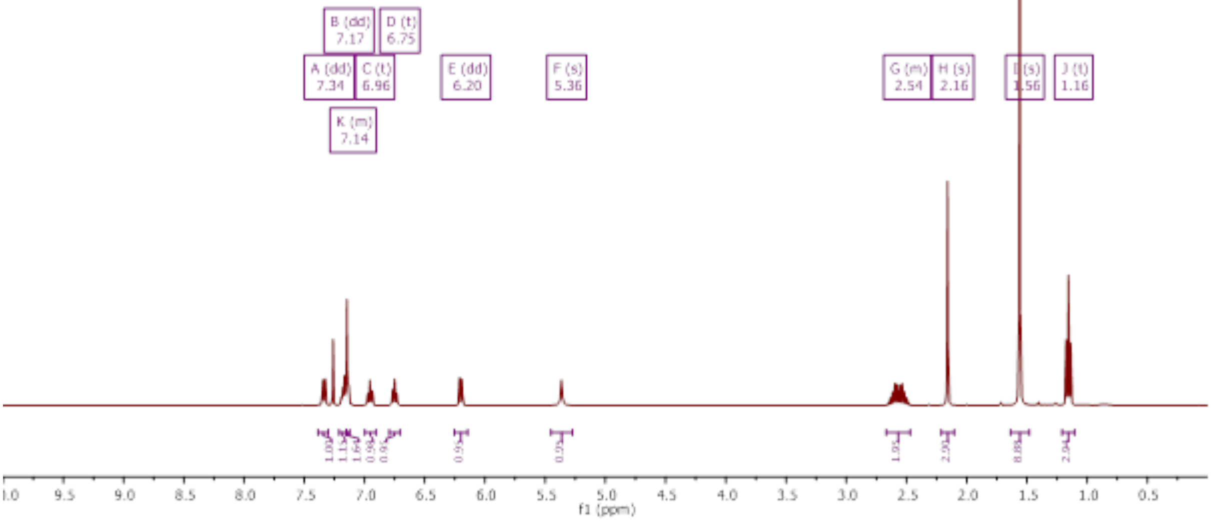

^{1}H NMR spectrum of **6i**

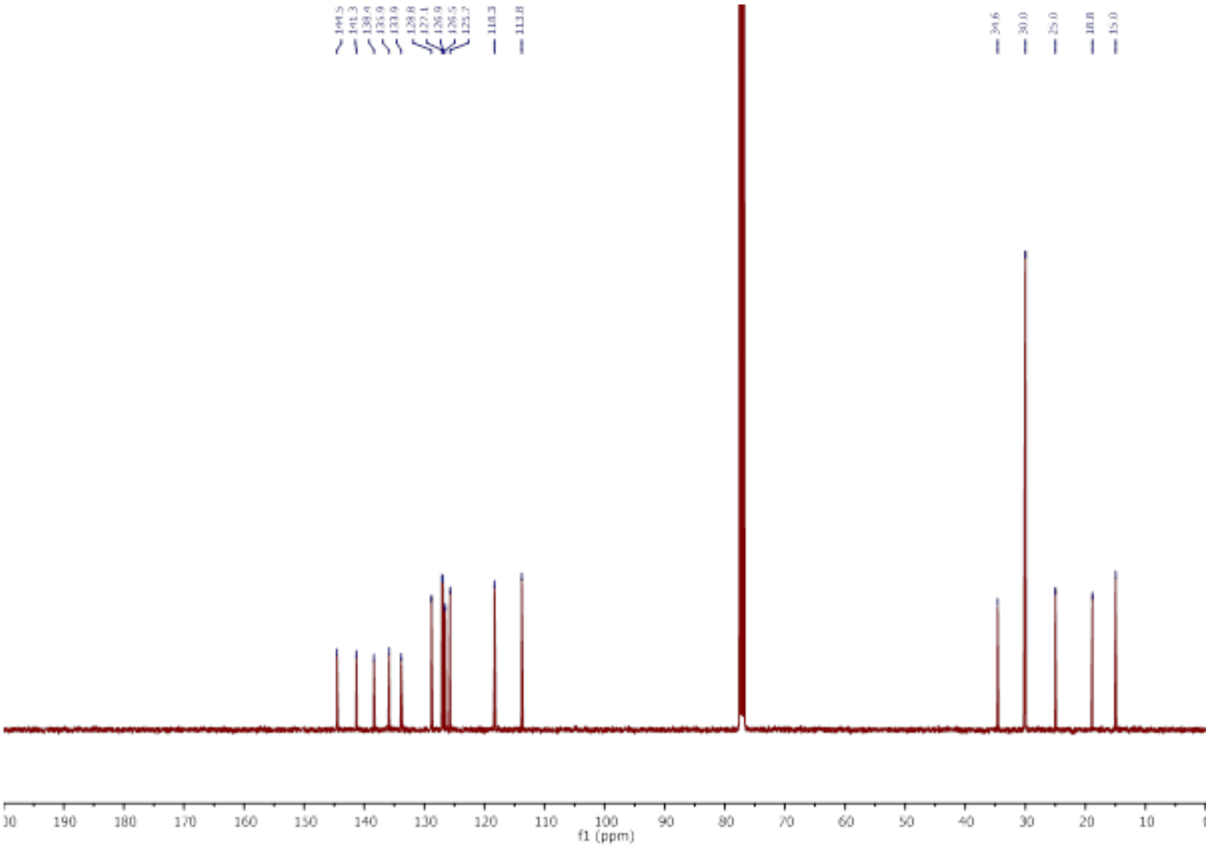

^{13}C NMR spectrum of **6i**

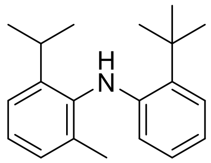

6j

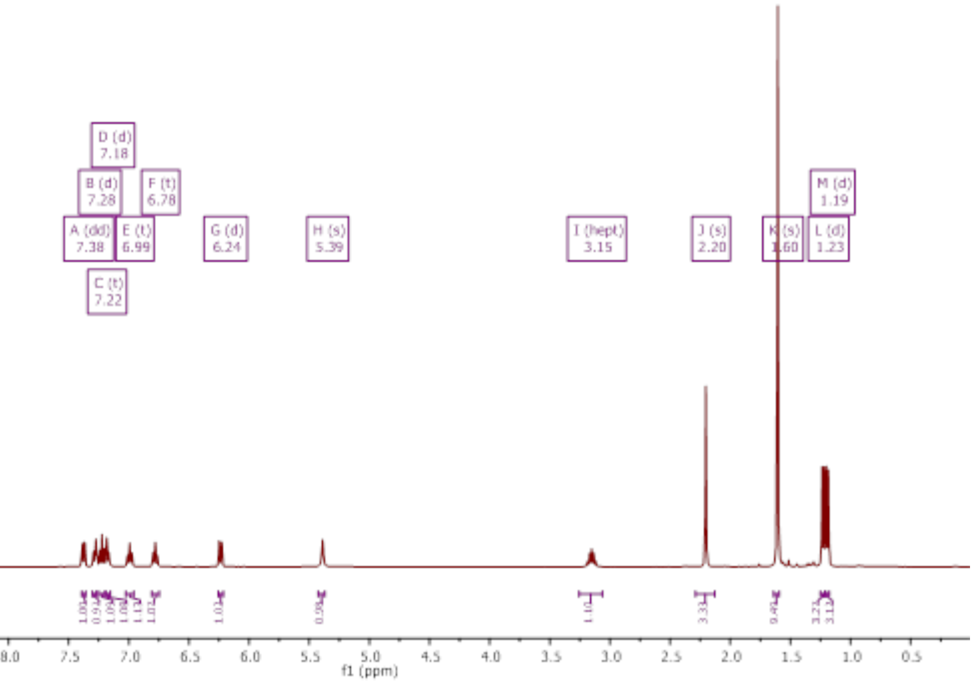

^{1}H NMR spectrum of **6j**

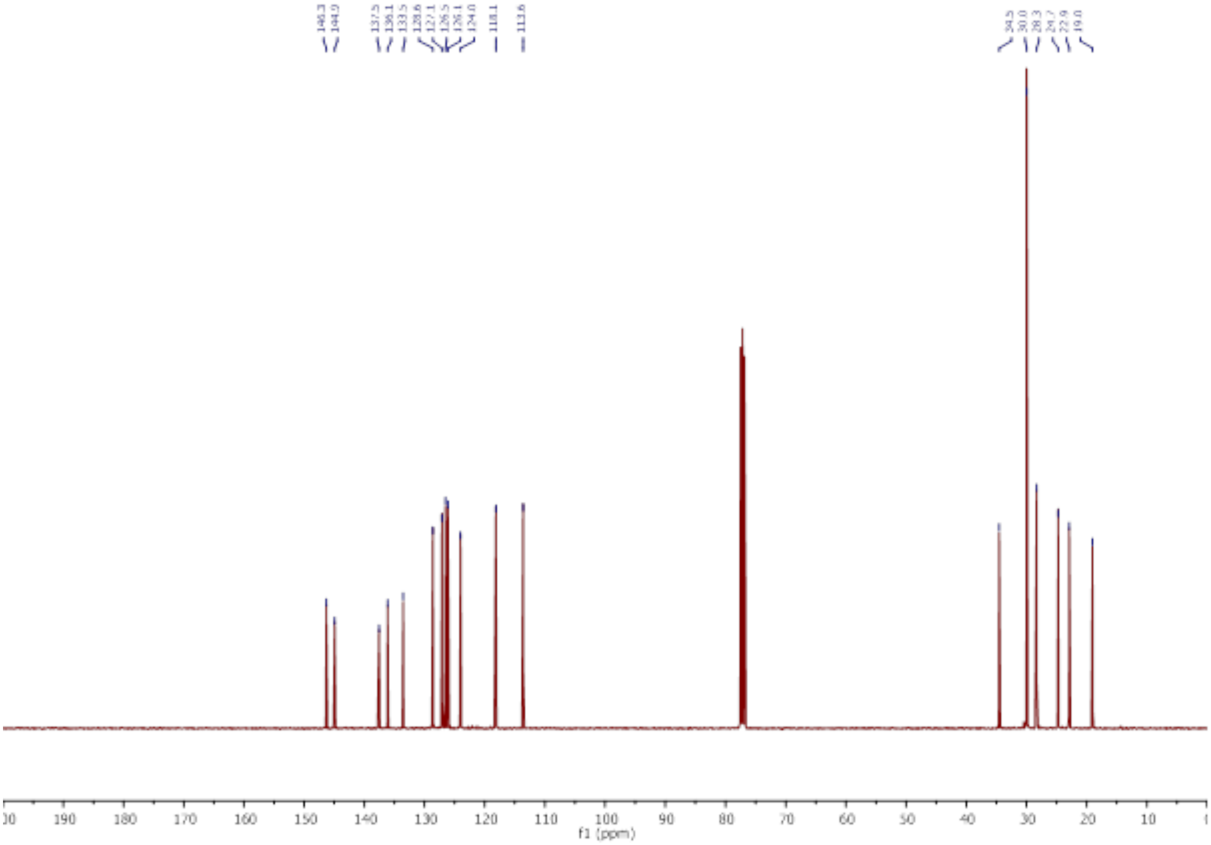

^{13}C NMR spectrum of **6j**

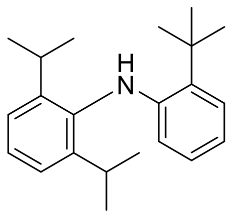

6k

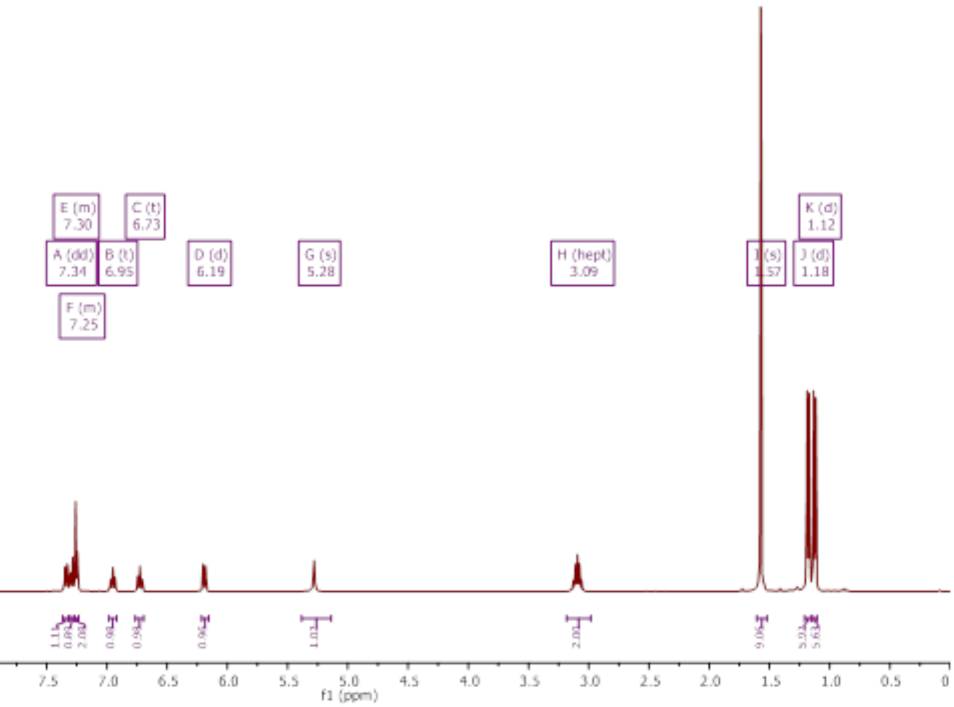

^{1}H NMR spectrum of **6k**

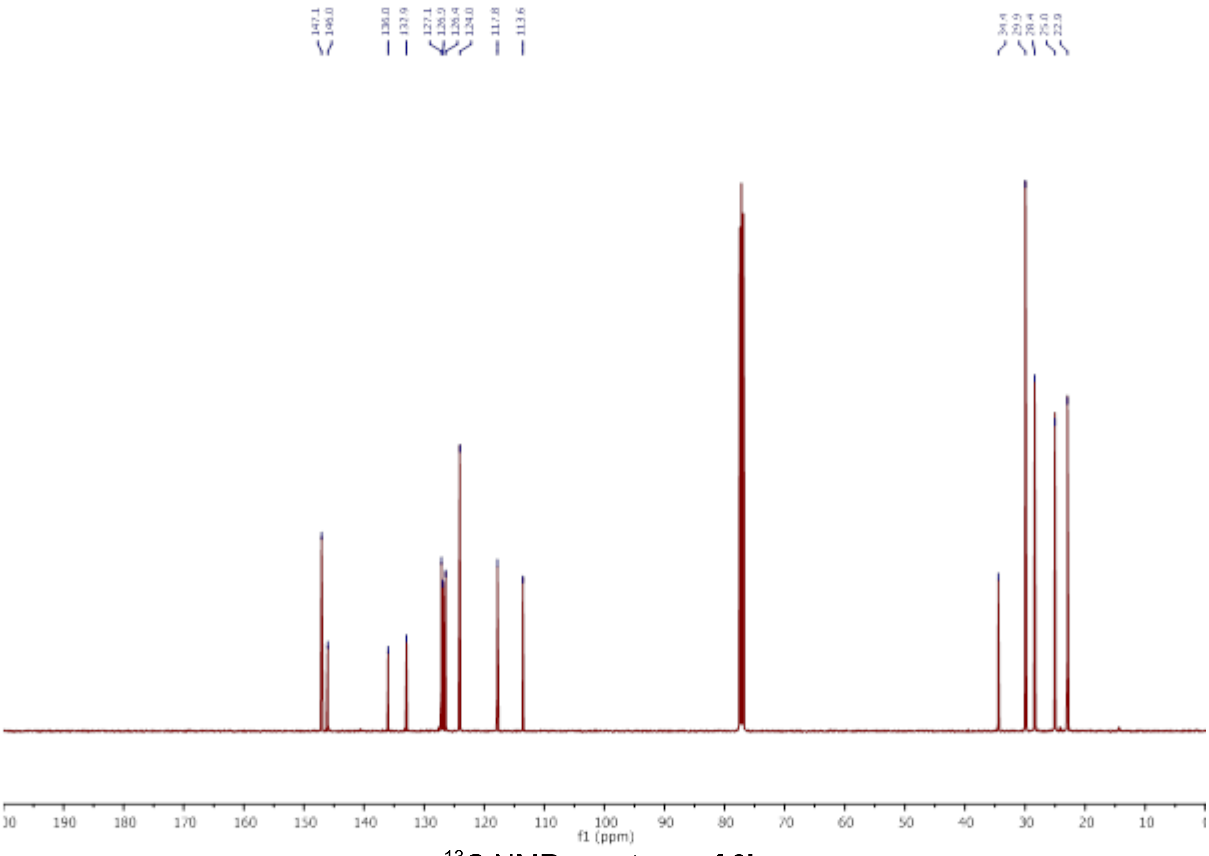

^{13}C NMR spectrum of **6k**

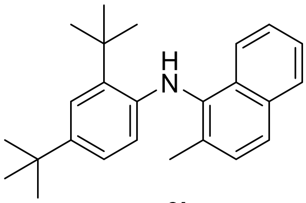

6l

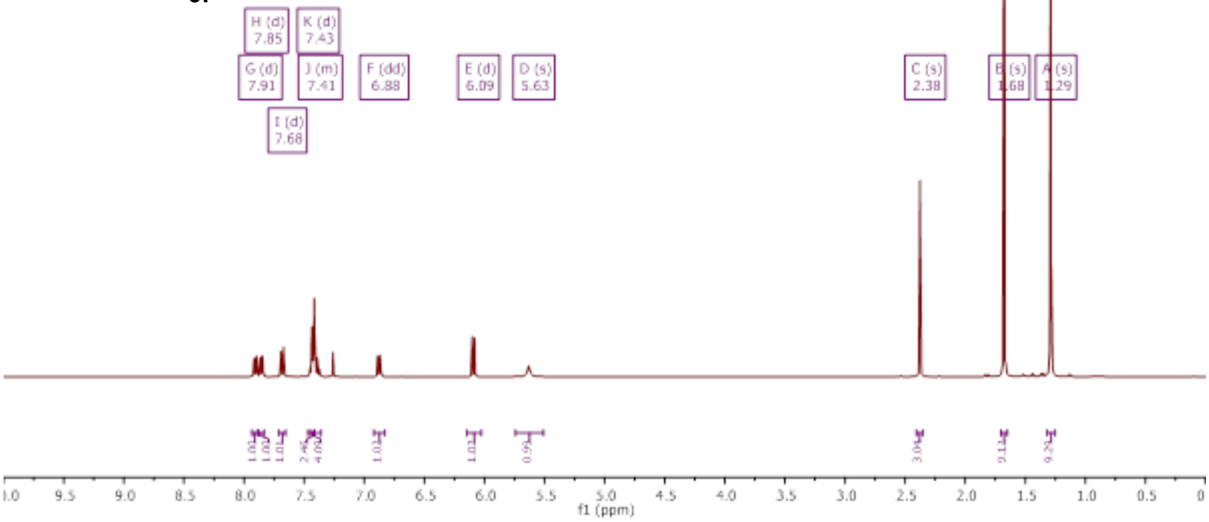

^{1}H NMR spectrum of **6l**

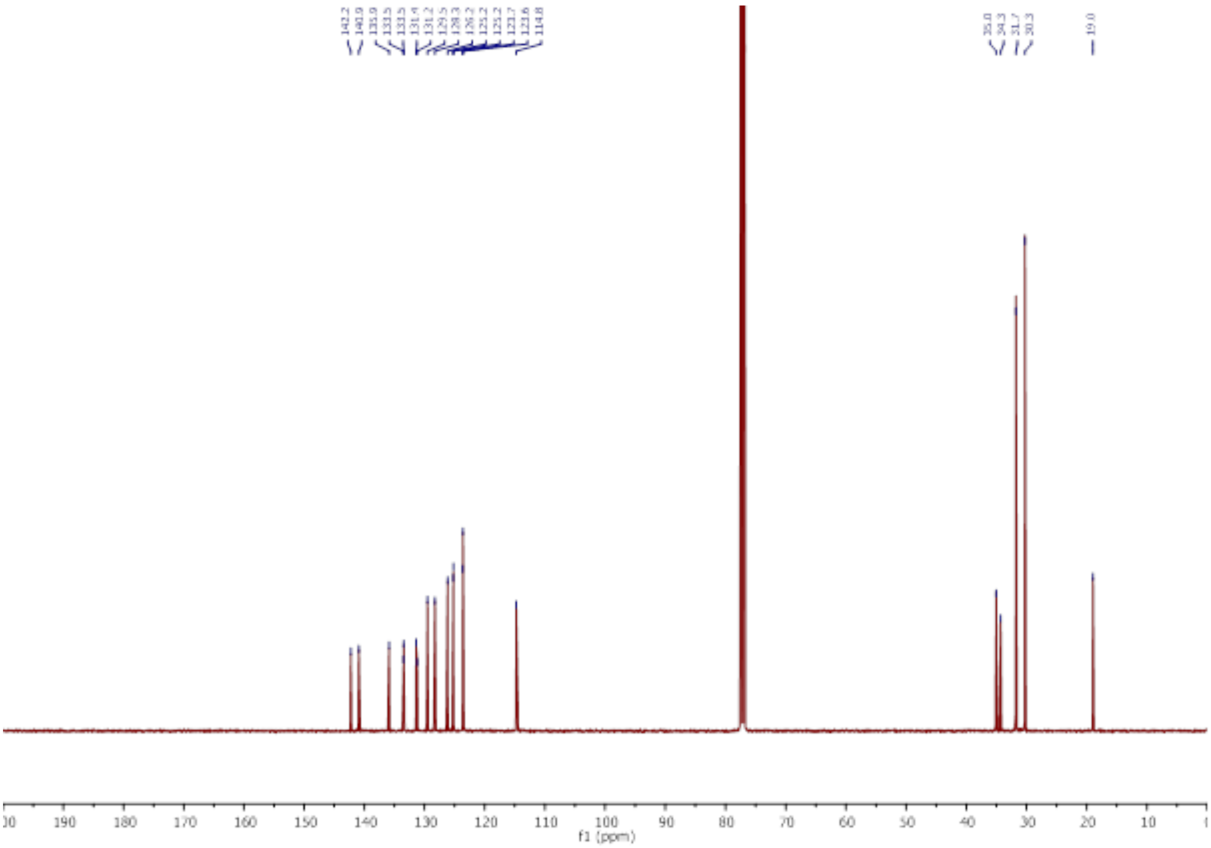

^{13}C NMR spectrum of **6l**

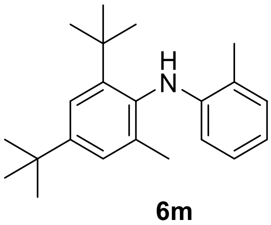

6m

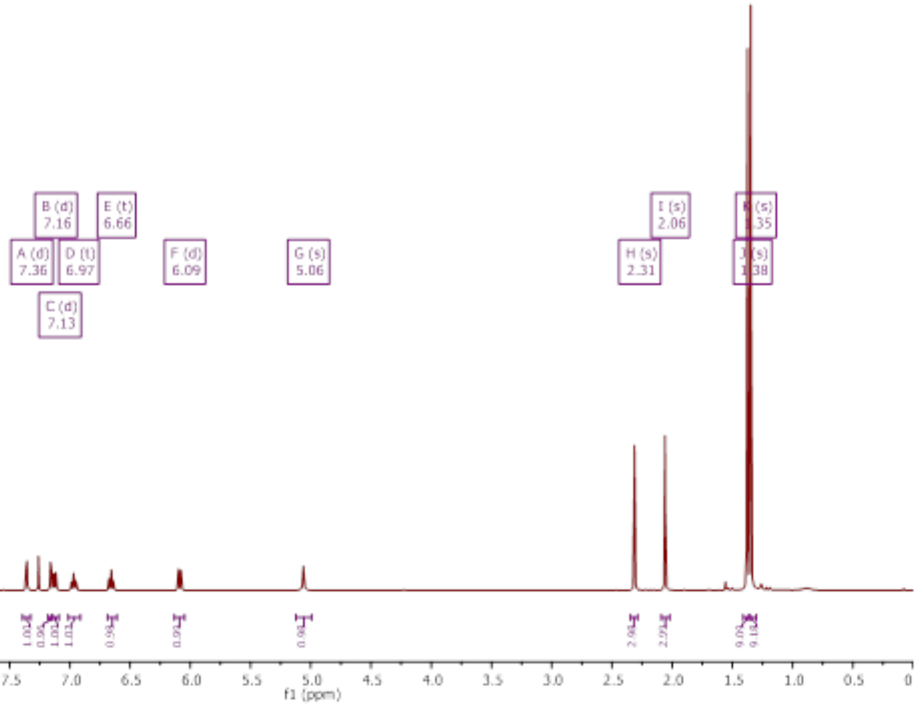

^{1}H NMR spectrum of **6m**

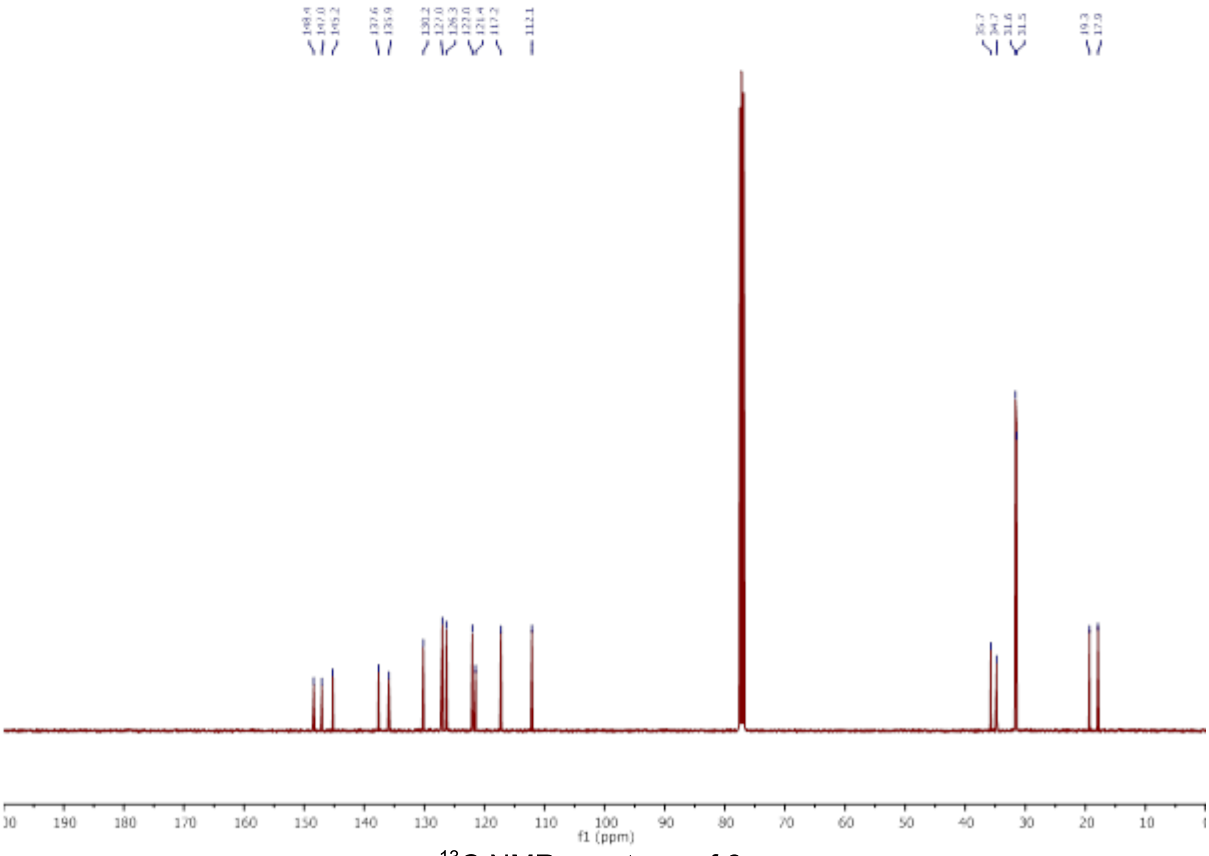

^{13}C NMR spectrum of **6m**

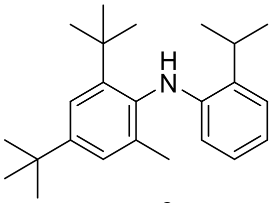

6n

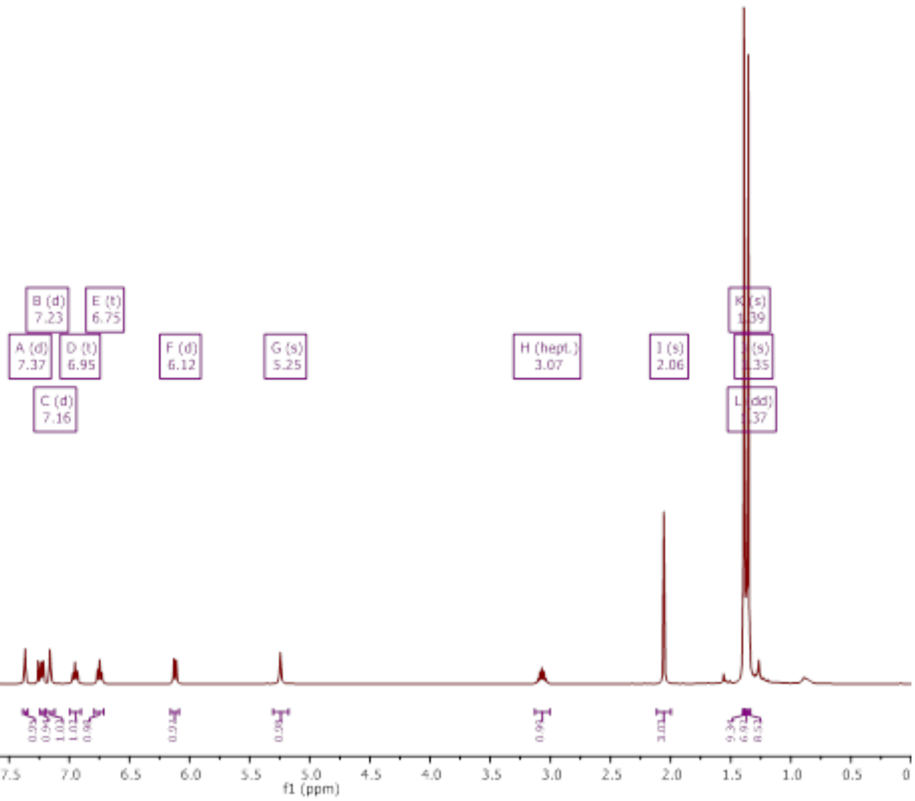

^{1}H NMR spectrum of **6n**

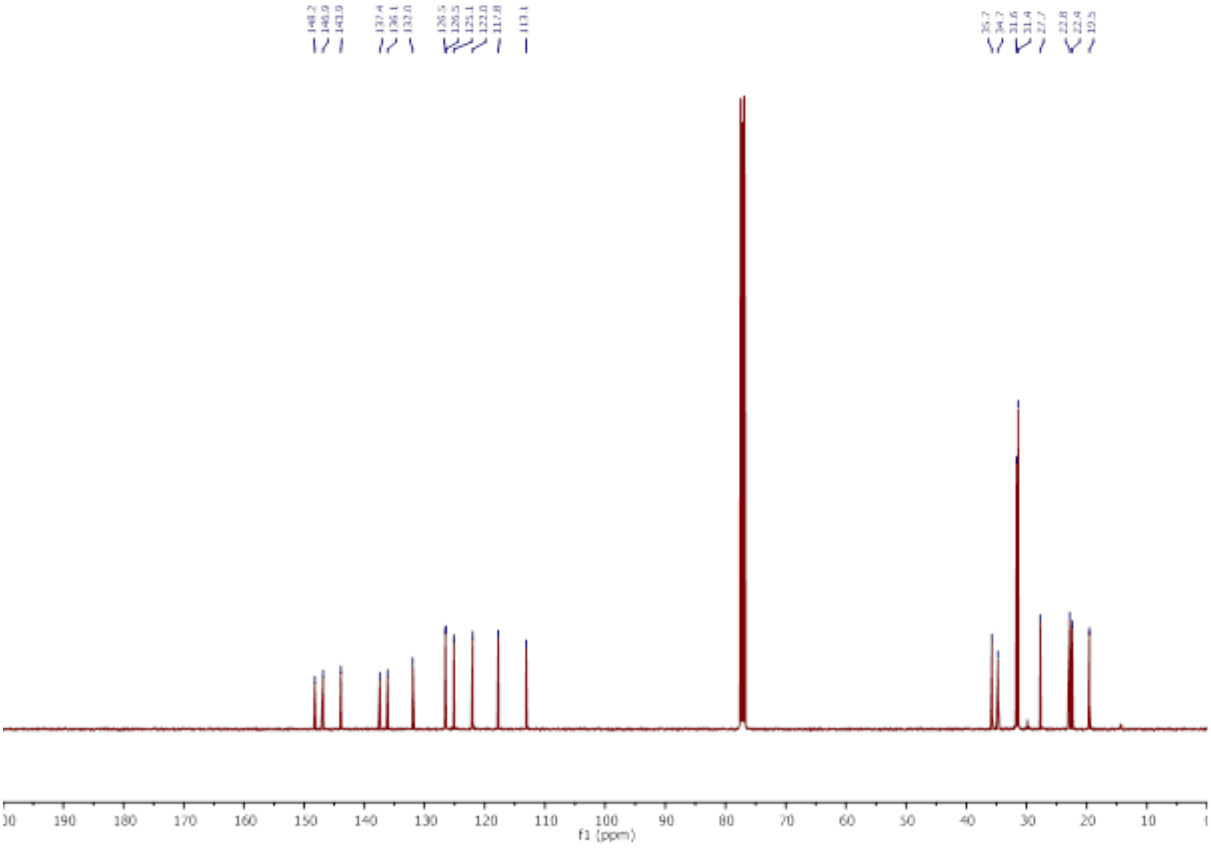

^{13}C NMR spectrum of **6n**

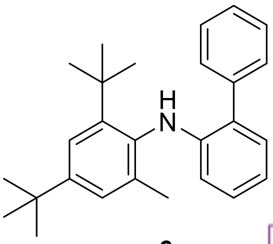

6o

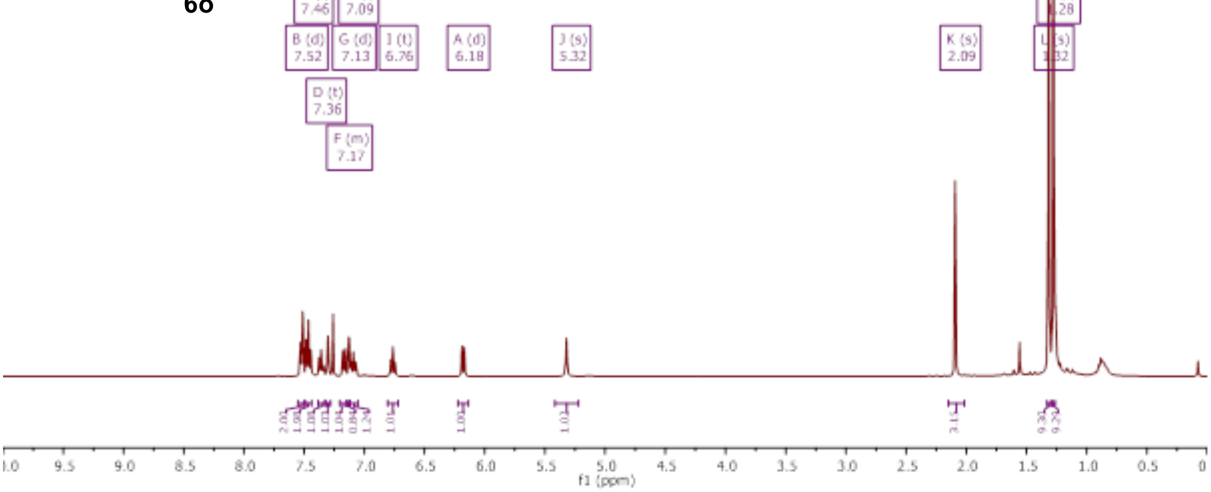

^{1}H NMR spectrum of **6o**

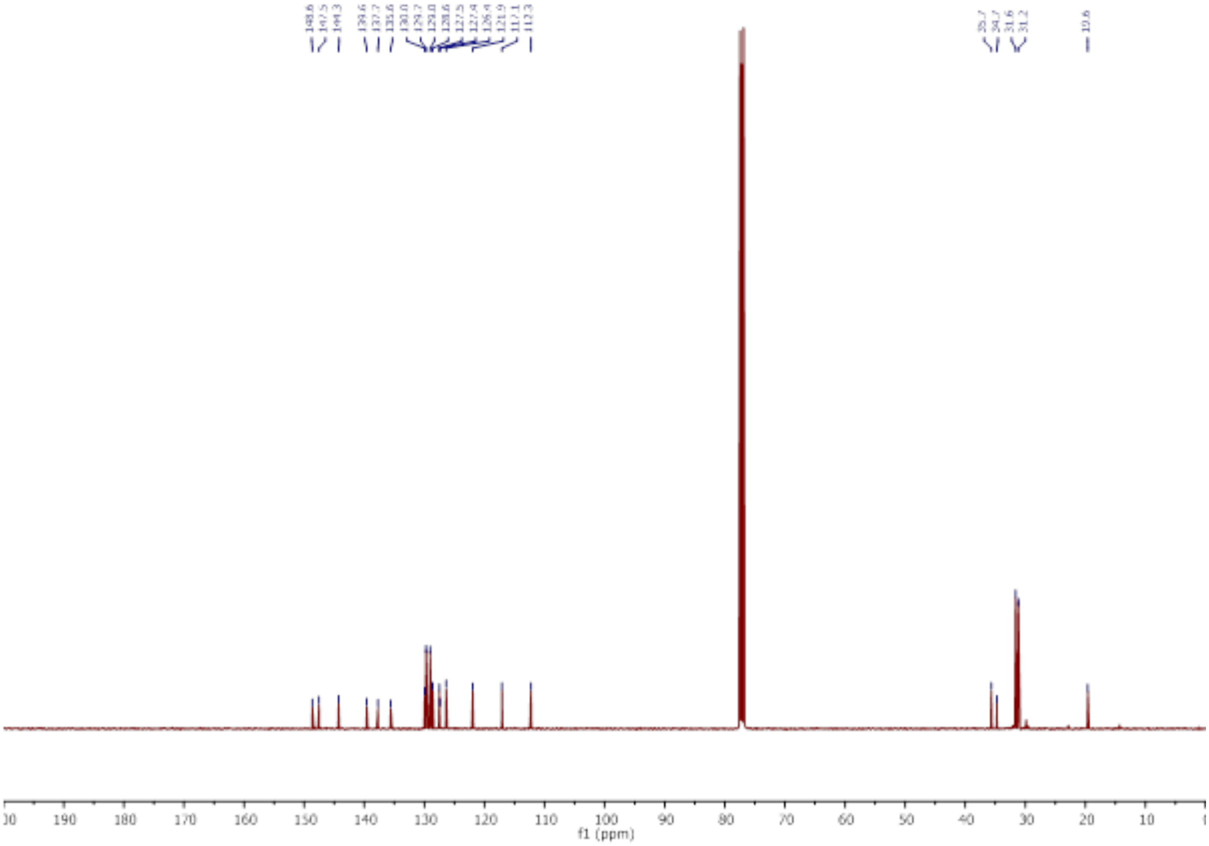

^{13}C NMR spectrum of **6o**

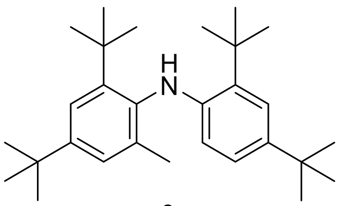

6p

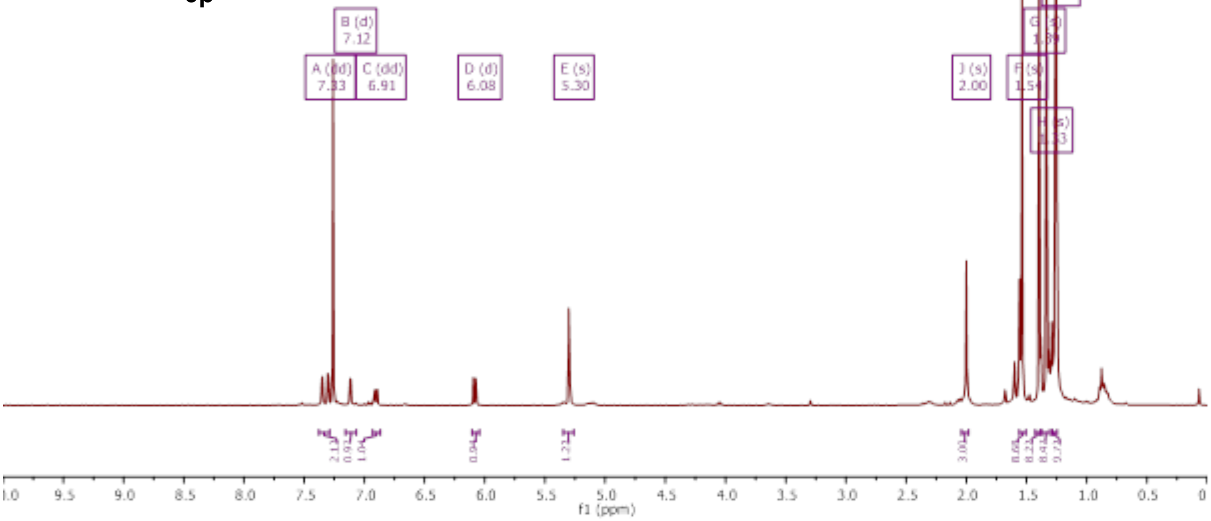

^{1}H NMR spectrum of **6p**

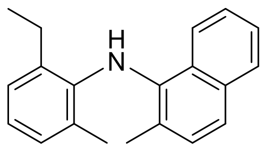

6q

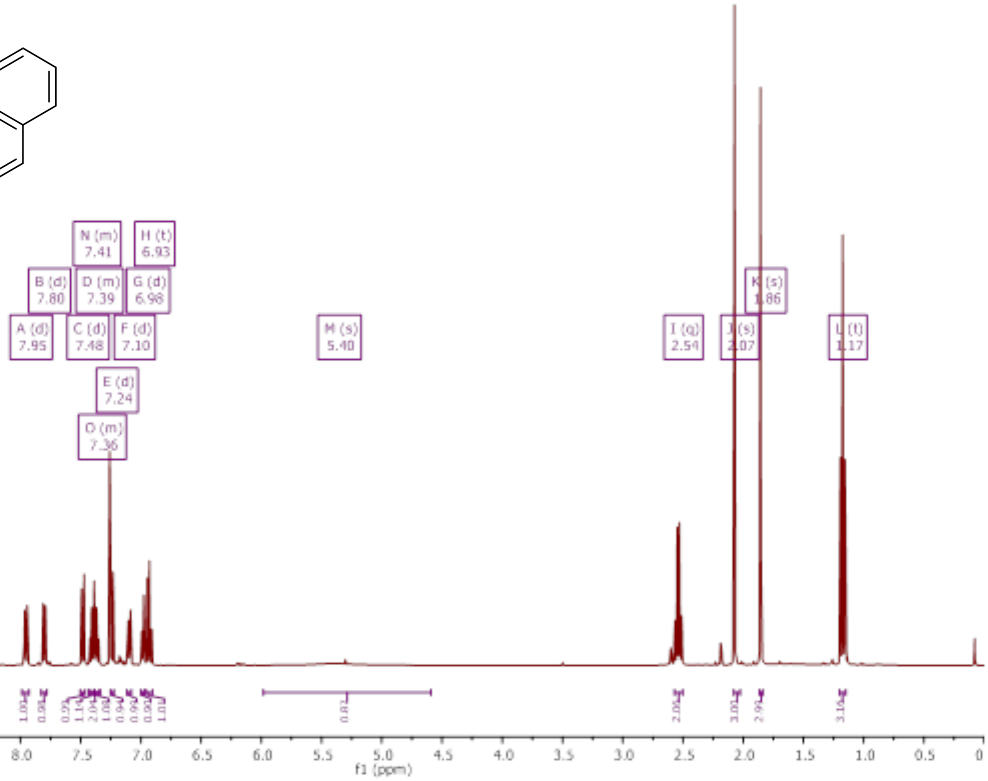

^{1}H NMR spectrum of **6q**

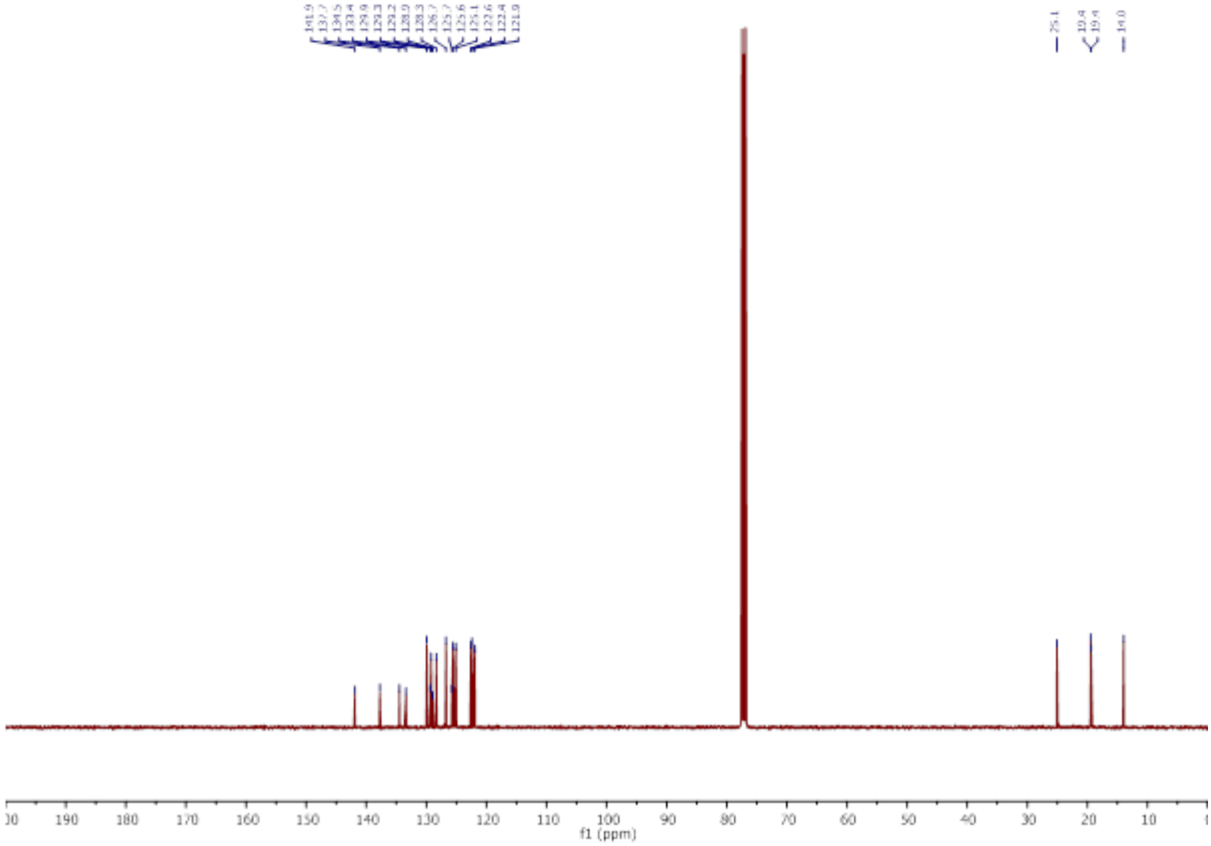

^{13}C NMR spectrum of **6q**

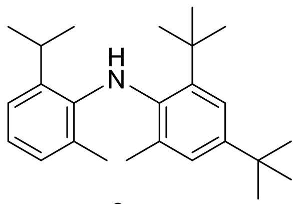

6r

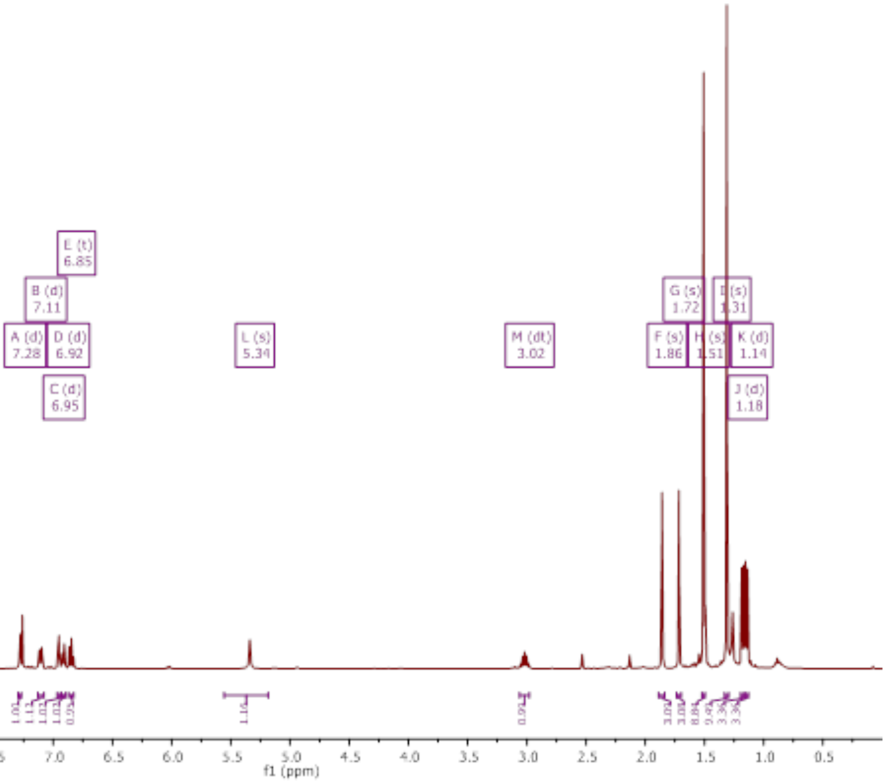

^{1}H NMR spectrum of **6r**

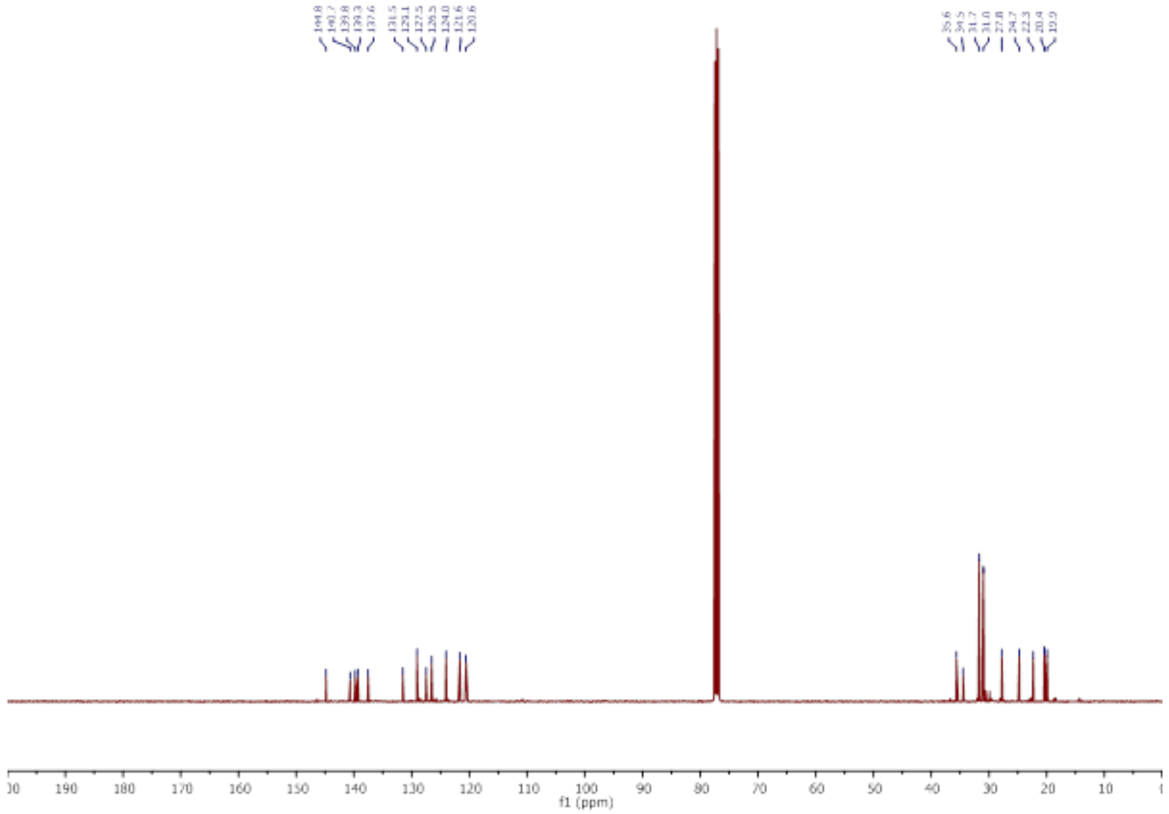

^{13}C NMR spectrum of **6r**

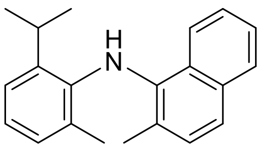

6s

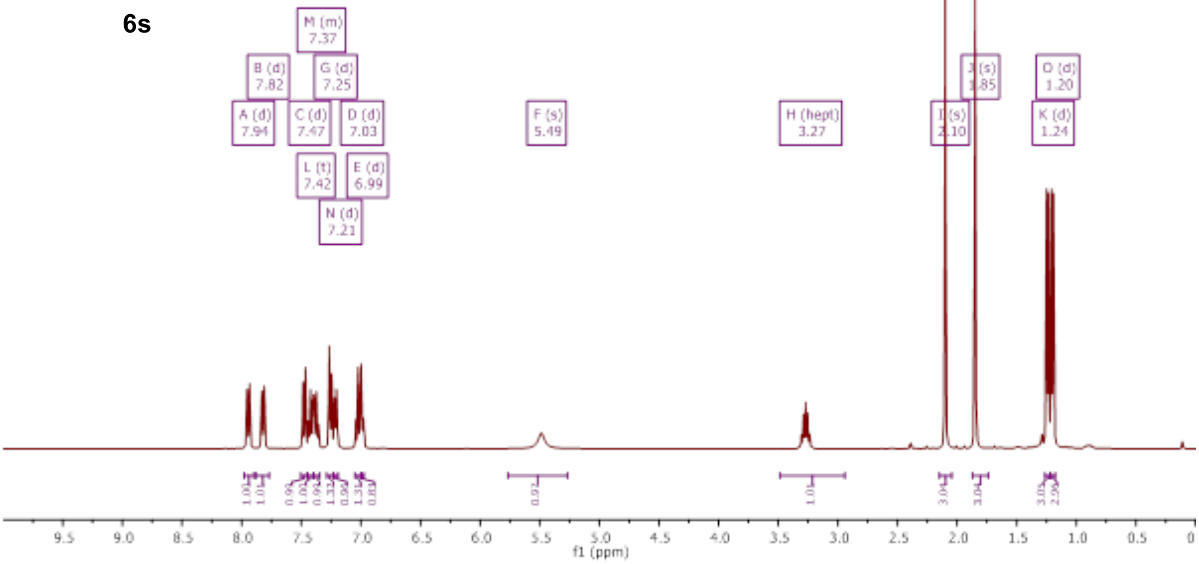

^{1}H NMR spectrum of **6s**

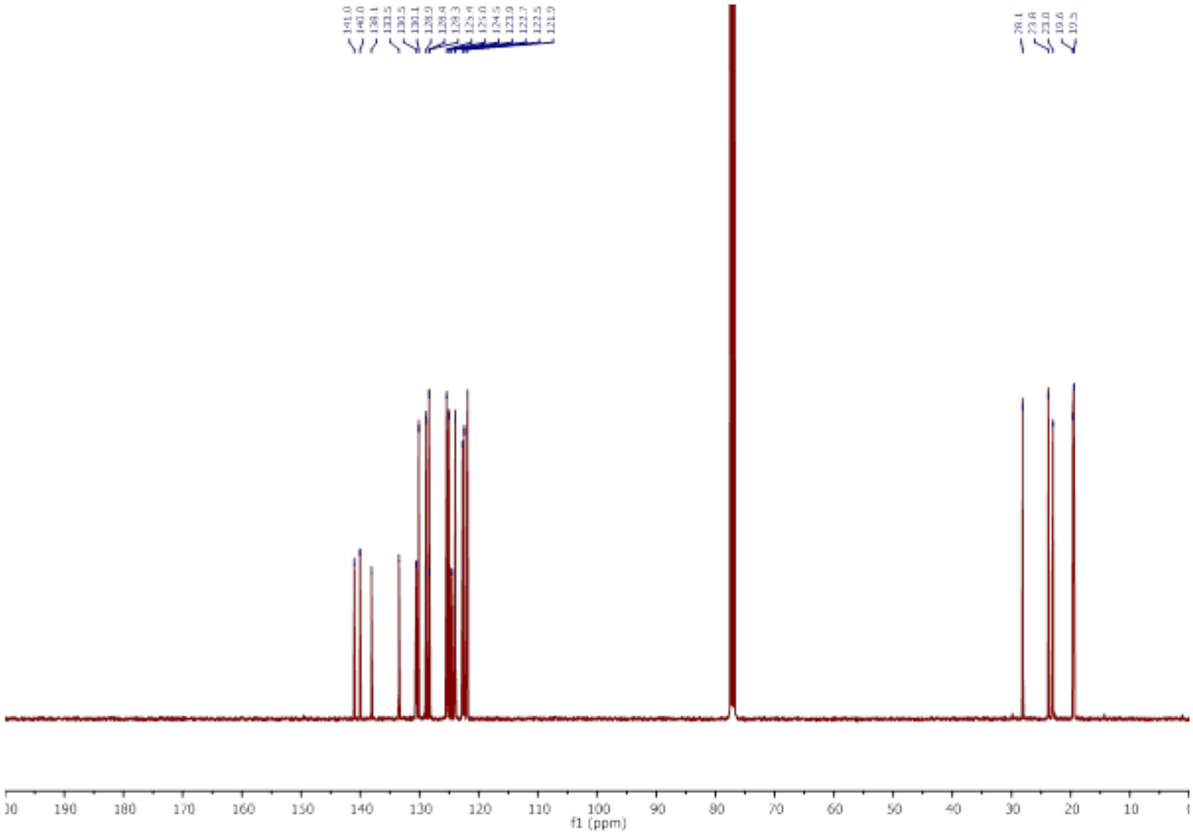

^{13}C NMR spectrum of **6s**

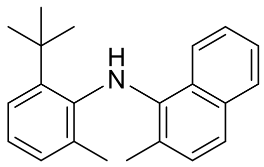

6t

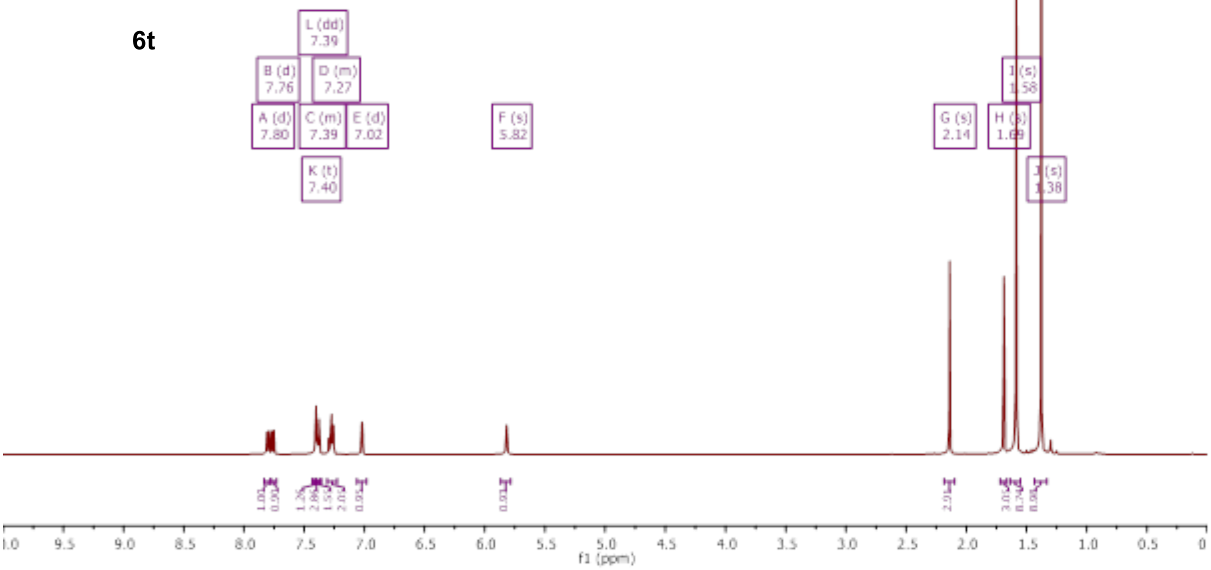

^{1}H NMR spectrum of **6t**

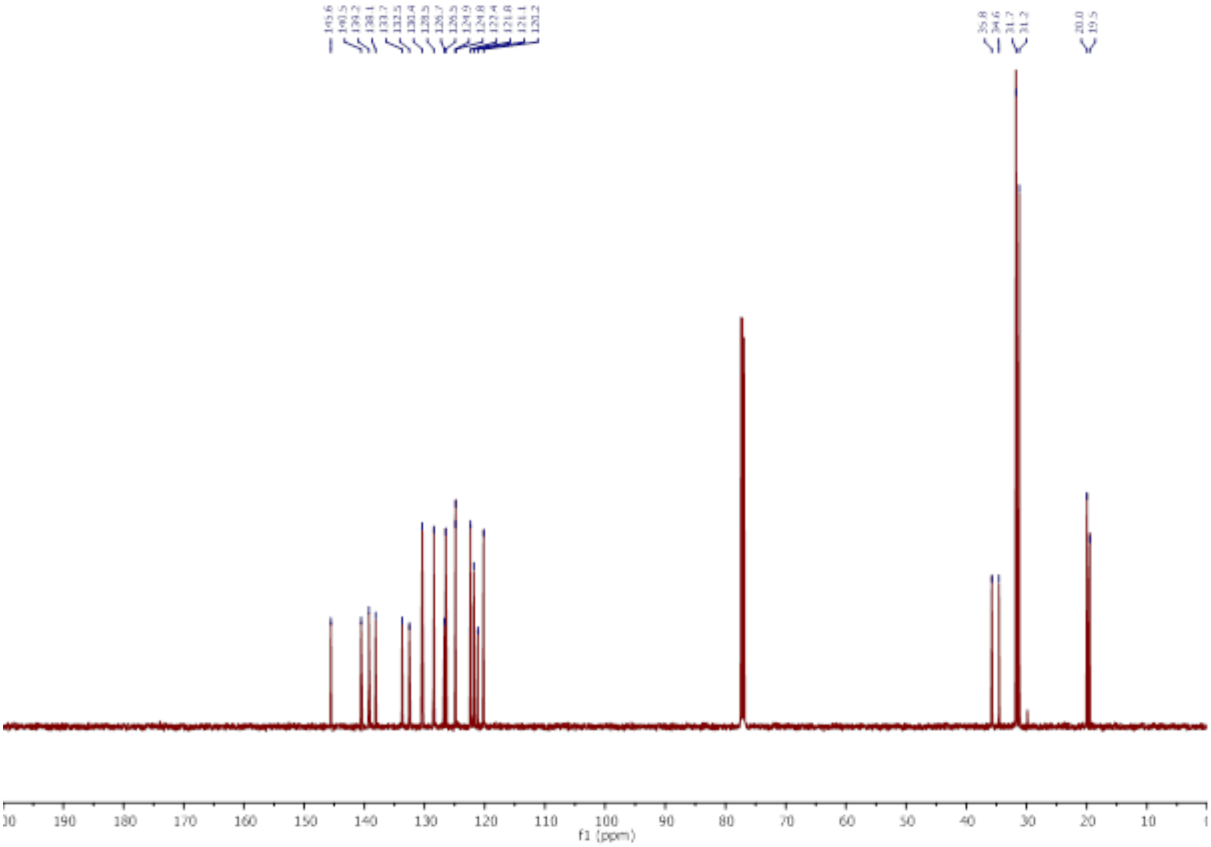

^{13}C NMR spectrum of **6t**

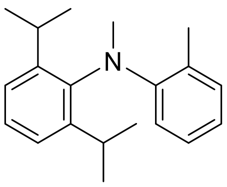

6g'

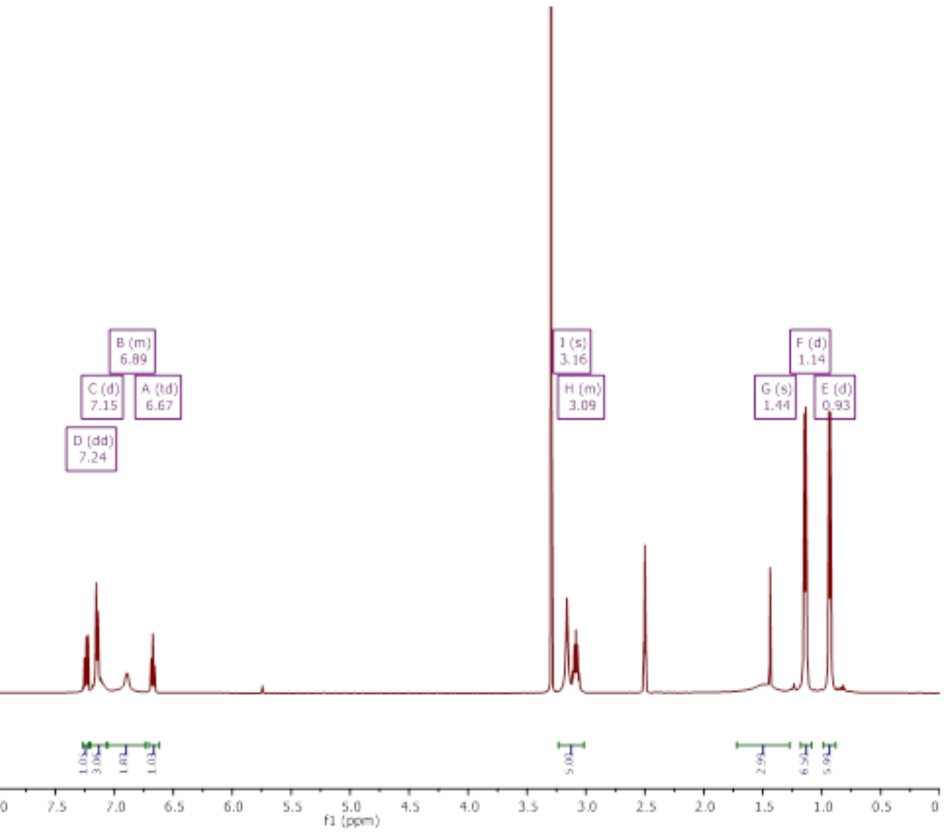

^{1}H NMR spectrum of **6g'**

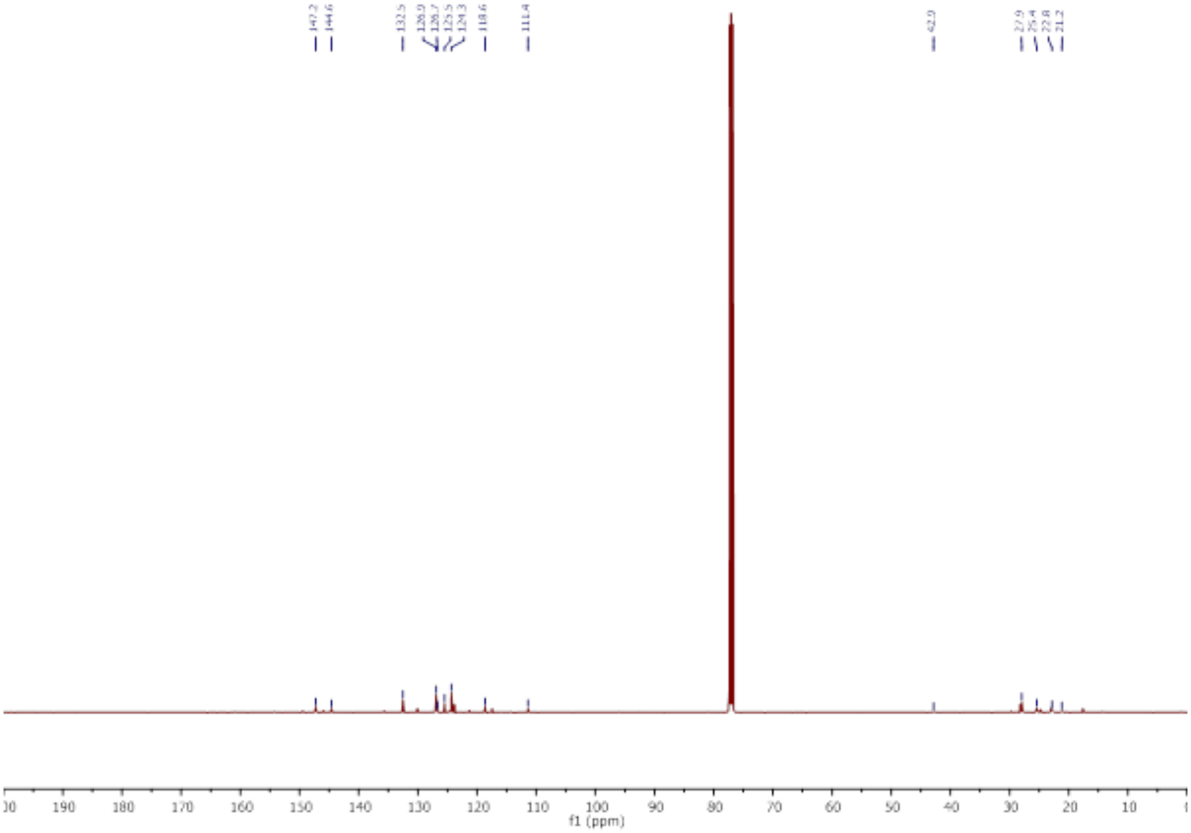

^{13}C NMR spectrum of **6g'**
